# Optimized Cas9‐Enriched Nanopore Sequencing and Analysis Workflow for Clinical Diagnosis of Repeat Expansion Disorders

**DOI:** 10.1002/advs.77475

**Published:** 2026-08-29

**Authors:** Seungbok Lee, Chanju Jung, Minjeong Kim, Narae Kim, Gue‐Ho Hwang, Soon‐Tae Lee, Kon Chu, Sang Kun Lee, Han‐Joon Kim, Jong‐Hee Chae, Sangsu Bae, Jangsup Moon

**Affiliations:** ^1^ Department of Genomic Medicine Seoul National University Hospital Seoul South Korea; ^2^ Department of Pediatrics Seoul National University College of Medicine Seoul National University Children's Hospital Seoul South Korea; ^3^ Department of Biomedical Sciences Seoul National University College of Medicine Seoul South Korea; ^4^ Department of Neurology Seoul National University College of Medicine and Seoul National University Hospital Seoul South Korea; ^5^ Department of Chemistry Hanyang University Seoul South Korea; ^6^ Cancer Research Institute and Genomic Medicine Institute Seoul National University College of Medicine Seoul South Korea; ^7^ Institute of Molecular Biology and Genetics Seoul National University Seoul South Korea

**Keywords:** Cas9 enrichment, methylation analysis, nanopore sequencing, repeat expansion, short tandem repeats, spinocerebellar ataxia

## Abstract

Short tandem repeat (STR) expansion is a major genetic mechanism underlying numerous neurogenetic disorders. However, traditional PCR amplification and short‐read next‐generation sequencing‐based methods often fail to detect large‐scale, complex expansions and to capture methylation information. Thus, this study aimed to modify an amplification‐free nanopore Cas9‐targeted sequencing (nCATS) platform to achieve uniform coverage across 56 currently defined STR loci using a single test with genomic DNA from patient‐derived blood cells and to develop a dedicated analysis algorithm, STRiker, capable of identifying internal motif contexts and *de novo* repeat structures. Ultimately, this study identified pathogenic repeat expansions in 12 of 37 patients (32.4%) with cerebellar ataxia who remained genetically undiagnosed despite extensive prior genetic testing, in *FGF14* (*n* = 4), *ATXN8OS*, *NOP56*, *RFC1* (*n* = 2 each), and *PRNP* and *NOTCH2NLC* (*n* = 1 each). Additionally, family‐based cascade screening revealed six relatives with repeat expansions in five families. These results demonstrate a broader diversity of pathogenic repeat structures, particularly in *FGF14*, and illustrate that CpG methylation can mitigate the pathogenic effects of repeat expansions. This nCATS–STRiker workflow offers a powerful strategy for improving the diagnosis of STR‐related neurogenetic diseases, such as cerebellar ataxia and other diseases.

## Graphical abstract

An optimized Cas9‐enriched nanopore sequencing workflow, combined with STRiker, enables simultaneous analysis of disease‐associated STR loci from patient blood. The nCATS–STRiker workflow detects repeat expansions, de novo repeat motifs, interruption patterns, and methylation in a single assay, improving the genetic diagnosis of previously undiagnosed cerebellar ataxia and providing intuitive visualization for clinical interpretation.

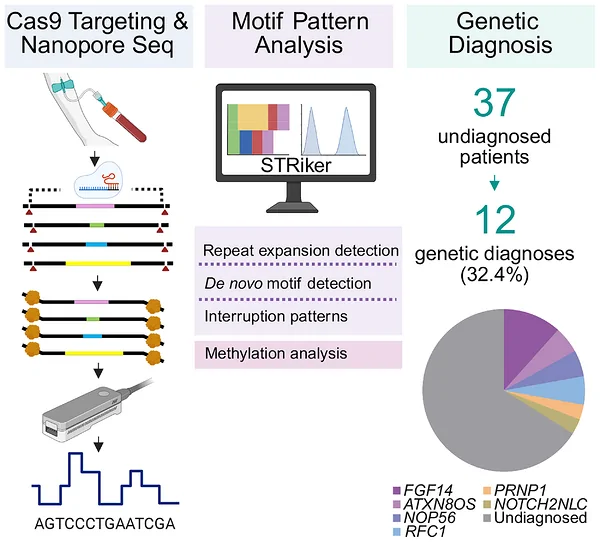

## 1 Introduction

Short tandem repeats (STRs) are repetitive sequences of short motifs, typically 2–7 base pairs in length. More than one million STR loci have been reported in the human genome [1], and STR expansions at specific sites can cause numerous genetic disorders [2]. The STR expansion loci associated with disease are continuously updated; as of the start of this study (i.e., late 2023), approximately 56 loci have been identified as causing Mendelian diseases, particularly neurological and neuromuscular [2]. For example, spinocerebellar ataxias (SCAs) are a group of genetically heterogeneous, progressive, and neurodegenerative diseases, many of which are caused by STR expansions at specific loci [3].

However, detecting and diagnosing disease‐causing STR expansions in patients remains challenging. Traditionally, fragment analysis methods based on PCR amplification and short‐read next‐generation sequencing (NGS) platforms have been used to diagnose STR‐related disorders. Although these methods are useful for detecting repetitive expansions, several important limitations remain. For example, conventional NGS methods, which typically generate read lengths of 150–300 bp, cannot accurately determine repeat structures when the pathogenic STR expansion (i.e., motif size × repeat count) exceeds this sequencing length limitation. Whole‐genome sequencing‐based approaches also fail to capture accurate repeat information, as the assembly of repetitive sequences remains highly challenging, leading to a loss of precise repeat‐count data. Furthermore, DNA methylation can be lost during PCR amplification.

To overcome these limitations, a PCR amplification‐free nanopore Cas9‐targeted sequencing (nCATS) method has been developed [4]. This nCATS approach uses CRISPR–Cas9 to enrich specific genomic regions for long‐read nanopore sequencing, enabling detection of small variants, STR expansions, and epigenetic modifications, such as methylation. Subsequent studies have demonstrated the utility of multiplexed nCATS for analyzing disease‐associated STR loci. For example, FLO‐SCAp targeted 16 SCA‐related loci using cost‐effective nanopore sequencing with a Flongle flow cell [5], and HMMSTR achieved genotyping across 60 loci in cell line‐derived samples [6]. Recently, Clin‐CATS demonstrated reliable enrichment across 17 ataxia‐associated loci in a clinical cohort of 513 blood‐derived samples [7], highlighting the potential of nCATS for characterizing repeat motif structures and interruption patterns. However, existing studies with multiplexed nCATS have focused on a limited number of STR loci in patient‐derived samples or have been restricted to evaluating larger STR panels primarily in cell line‐derived samples. Furthermore, existing analytical tools focus primarily on repeat‐length estimation and predefined motif analysis, limiting their ability to identify motif contexts and interruption patterns intuitively, while preserving atypical patient‐specific repeat structures.

Thus, this study aimed to optimize the nCATS method to enable a single test to simultaneously diagnose all known STR expansion loci. This study employed the following strategies: (i) we selected optimal guide RNAs (gRNAs) with the best performance at each gene based on in vitro cleavage (IVC) assays; (ii) we designed each gRNA to generate approximately 10 kb fragments to minimize the existing length bias in nanopore sequencing; (iii) we developed a dedicated program, STRiker, to detect *de novo* STR contexts and interruption patterns. Building on the optimized nCATS–STRiker workflow, we tested this framework in 37 patients with cerebellar ataxia who had undergone extensive genetic testing, including PCR‐based assays, whole‐exome sequencing, and whole‐genome sequencing, yet remained genetically undiagnosed. Subsequently, we newly diagnosed 12 of these 37 patients (32.4%); the process from DNA extraction to diagnosis was completed within 25 h. We further identified several *de novo* repeat contexts, particularly in *FGF14*, and methylation changes during transmission in *NOTCH2NLC*. Collectively, our optimized nCATS–STRiker workflow can provide a fast and precise strategy for enhancing the diagnosis of STR‐related neurogenetic diseases such as cerebellar ataxia.

## 2 Results

### 2.1 Optimization of gRNAs to Maximize IVC Efficiency

**FIGURE 1:**
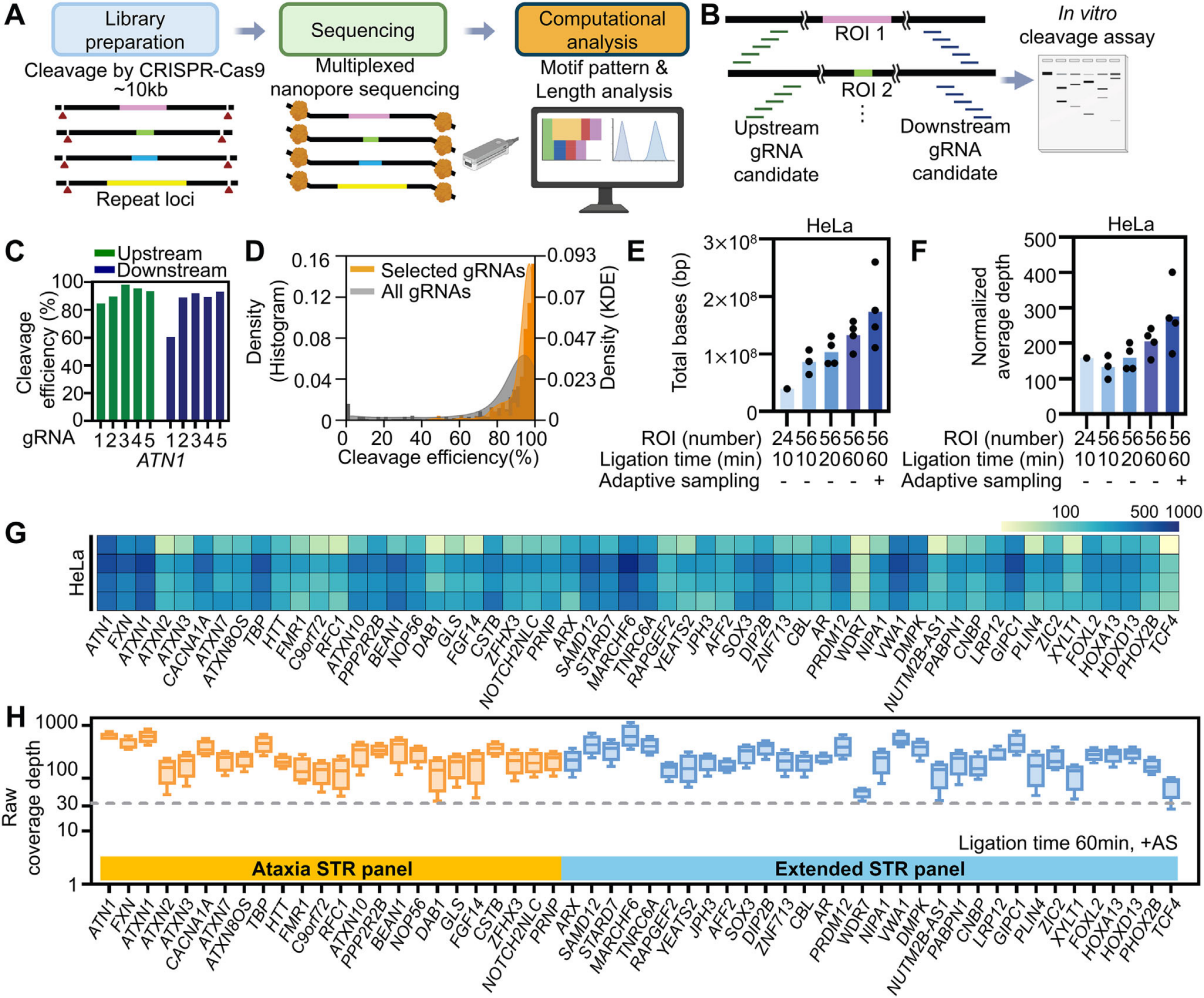
Optimization of the nanopore Cas9‐targeted sequencing (nCATS) workflow for multiplexed short tandem repeat (STR) analysis. (A) Overview of the nCATS pipeline. DNA fragments of approximately 10 kb surrounding STR regions were excised by CRISPR–Cas9 and sequenced on Oxford Nanopore. Computational analysis allowed visualization of repeat motif patterns and read‐length distributions. Created with BioRender.com. (B) Schematic of guide RNA (gRNA) design and in vitro cleavage (IVC) assay. Three or five gRNA candidates were tested on each side of the region of interest (ROI) to identify the most efficient pair. Created with BioRender.com. (C) IVC results for upstream and downstream gRNAs targeting the *ATN1* locus. Cleavage efficiency is shown for each guide (*n* = 1). (D) Histogram and kernel density estimate (KDE) of changes in cleavage efficiency between selected gRNAs (orange) and all tested gRNAs (gray); most selected gRNAs exhibited >90% cleavage efficiency. (E, F) Effect of adapter ligation time and adaptive sampling (AS) on sequencing yield and coverage. (G) Heatmap of the per‐locus sequencing coverage across 56 targeted STR loci in HeLa cells using ligation with AS for 60 min. (H) Raw coverage depth for each target locus is summarized across all sequencing runs in HeLa cells using ligation with AS for 60 min, indicating uniform read depth across all genes included in the ataxia and extended STR panels. The ataxia STR panel (orange) includes genes associated with cerebellar ataxia, and the extended STR panel (blue) includes additional STR‐related genes.

First, we conducted in vitro experiments to improve the nCATS method and evaluate all existing STR regions in a single test (Figure 1A). We grouped 56 STR regions into two panels for which associations with genetic disorders were either known or suggested (Figure S1): the ataxia STR panel included STR loci associated with cerebellar ataxia, whereas the extended STR panel encompassed all other loci implicated in STR‐related genetic disorders. Since nanopore sequencing reads shorter DNA fragments more abundantly [8, 9], we designed gRNAs to generate approximately 10 kb DNA fragments for nCATS, including the region of interest (ROI), to minimize length bias (Figure 1A). We conducted IVC assays with three or five gRNA candidates positioned upstream and downstream of each ROI (Figure 1B). These experiments used single‐guide RNA (sgRNA) formed by linking the dual RNA components of CRISPR RNA (crRNA) and trans‐activating crRNA (tracrRNA). Ultimately, we selected a pair of gRNAs that showed the best DNA cleavage activities upstream and downstream of each ROI (Figure 1C and Figure S2). The gRNAs chosen for the final panel exhibited higher cleavage efficiencies, with most exceeding 90%, compared with the other gRNA candidates (Figure 1D and Figure S3A).

Next, we compared the cleavage efficiency of Cas9 guided by sgRNA vs. tracrRNA:crRNA. The sgRNA construct has been widely used because of its simpler structure compared with tracrRNA:crRNA [10, 11, 12, 13, 14, 15]. However, we observed that Cas9/tracrRNA:crRNA achieved cleavage efficiency comparable to Cas9/sgRNA (Figure S3B). Since sgRNA is approximately 100 nucleotides long, whereas crRNA and tracrRNA are about 36 and 67 nucleotides, respectively, we determined that the Cas9/tracrRNA:crRNA complex would be advantageous in terms of cost and flexibility for target changes (Figure S3C). Additionally, we performed experiments to determine the optimal crRNA:tracrRNA combination ratio in vitro. Based on these results, we used 6 pmol tracrRNA and 3 pmol crRNA per ROI in all subsequent experiments (Figure S3D).

### 2.2 Optimization of the nCATS Protocol to Ensure Uniform Coverage of STR Regions

With the selected 56 gRNA pairs, we next evaluated the nCATS protocol to ensure adequate coverage depth and more uniform coverage across the STR target panel. Using genomic DNA (gDNA) extracted from HeLa cells, we tested the nCATS protocol with three adjustments: (i) modification of the dA‐tailing step into separate steps, (ii) extension of the adapter ligation time, and (iii) application of nanopore adaptive sampling (Figure S4). Typically, Cas9 cleavage and dA‐tailing were performed together. However, we speculated that any residual Cas9 following cleavage might interfere with the dA‐tailing reaction; therefore, we separated the process into two steps: Cas9 was first removed via bead purification, and then dA‐tailing was performed. While the manufacturer recommends a 10‐min ligation, extending this step beyond the standard protocol has become common practice to improve sequencing yield. Rather than adopting an arbitrary extended duration, we sought to empirically determine the optimal ligation time for our 56‐locus STR panel by directly comparing 10‐, 20‐, and 60‐min conditions (Figure 1E–H and Figure S5A). This quantitative comparison confirmed that both 20‐ and 60‐min conditions substantially outperformed the standard 10‐min condition, with 60 min yielding the highest overall sequencing output. Application of adaptive sampling reduced reads originating from non‐target regions and enriched the concentration of target regions across the entire panel, further improving the overall coverage depth across STR loci (Figure 1G,H and Figure S5B). Ultimately, we achieved a normalized average length coverage of 275 × across all 56 STR loci, indicating that our optimized nCATS method can be applied to diagnose all known STR‐related diseases (Figure 1F).

To evaluate coverage uniformity across all target loci, we created heatmaps to visualize per‐locus read depth. When we applied the optimized nCATS workflow to HeLa samples using the full panel comprising 56 STR regions (i.e., the comprehensive STR panel), the coverage was evenly distributed across most targets, as indicated by the relatively uniform color patterns across the rows, which represent loci per individual (Figure 1G). Quantitative analysis further showed that raw coverage depth was consistent across loci throughout the panel (Figure 1H). These results demonstrate uniform per‐locus coverage across all 56 targets in multiplexed STR analysis (Figure S5A).

### 2.3 Application of the Optimized nCATS Method for Patient‐Derived Blood Cells

Building on the optimized nCATS workflow, we next applied this modified method to gDNA from patient‐derived blood cells. Unlike gDNA from HeLa cells, which is abundantly available in the laboratory, gDNA from patient‐derived blood cells is often limited in quantity. Therefore, we attempted to use minimal amounts of gDNA from patient‐derived blood cells (i.e., 5 µg), in contrast to the previously applied 10 µg of gDNA from HeLa cells used to optimize the nCATS protocol. As part of the proof of concept, we tested 15 patient‐derived gDNA samples with 24 STR regions (i.e., ataxia STR panel) and seven samples with the full panel of 56 STR regions (i.e., comprehensive STR panel).

**FIGURE 2:**
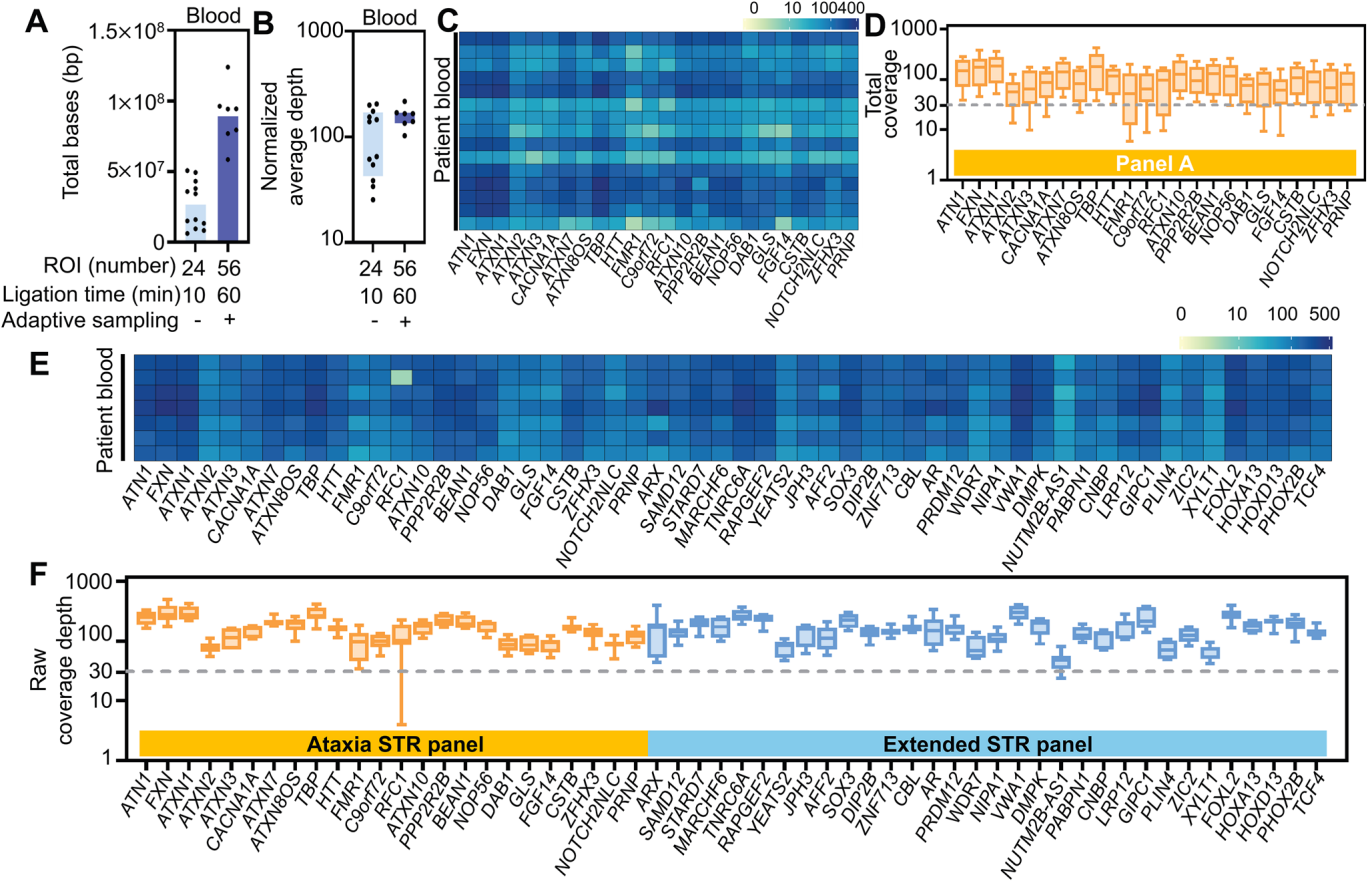
Application of the optimized nCATS workflow to patient blood samples. (A, B) Effect of ligation time (10 vs. 60 min) and adaptive sampling on total bases (A) and normalized sequencing depth (B). (C–F) Sequencing coverage across blood‐derived samples. Heatmaps show per‐locus sequencing depth, where color intensity represents read depth per locus and demonstrates consistent coverage distribution. Boxplots summarize total coverage per STR locus; a consistent distribution of coverage was observed. (C, D) Results from the 24‐locus panel (ataxia STR panel). (E, F) Results from the 56‐locus panel (comprehensive STR panel).

Consistent with the results from HeLa cells, sufficient sequencing yield was achieved in patient‐derived samples despite the increased number of ROIs (Figure 2A). Using patient‐derived gDNA samples, the length‐normalized average depth reached 108 × across 24 loci and 156 × across all 56 loci (Figure 2B). Although the total number of base reads obtained from patient‐derived gDNA samples was slightly lower than that yielded from HeLa cells, the sequencing depth was sufficient to diagnose the STR‐related genetic diseases.

To confirm whether the uniform coverage achieved in HeLa cells could be reproduced in clinical samples, heatmaps were again generated to visualize the per‐locus read depth in patient‐derived gDNA. When we treated samples with the ataxia STR panel, the coverage appeared evenly distributed across most targets, as indicated by the relatively uniform color patterns across rows (i.e., loci per individual) (Figure 2C,D). Similarly, when we treated samples with the comprehensive STR panel, the coverage appeared evenly distributed across most targets (Figure 2E,F). In addition, this workflow could be easily scaled up to a 62‐locus panel, demonstrating its scalability (Figure S6). Thus, these findings validate that the uniform coverage established during HeLa‐based optimization is stably reproducible in patient‐derived samples, supporting the robustness and clinical scalability of the optimized nCATS method and confirming its usability for comprehensive STR analysis.

### 2.4 STRiker‐Based Analysis of STR Expansion at 56 Loci

**FIGURE 3:**
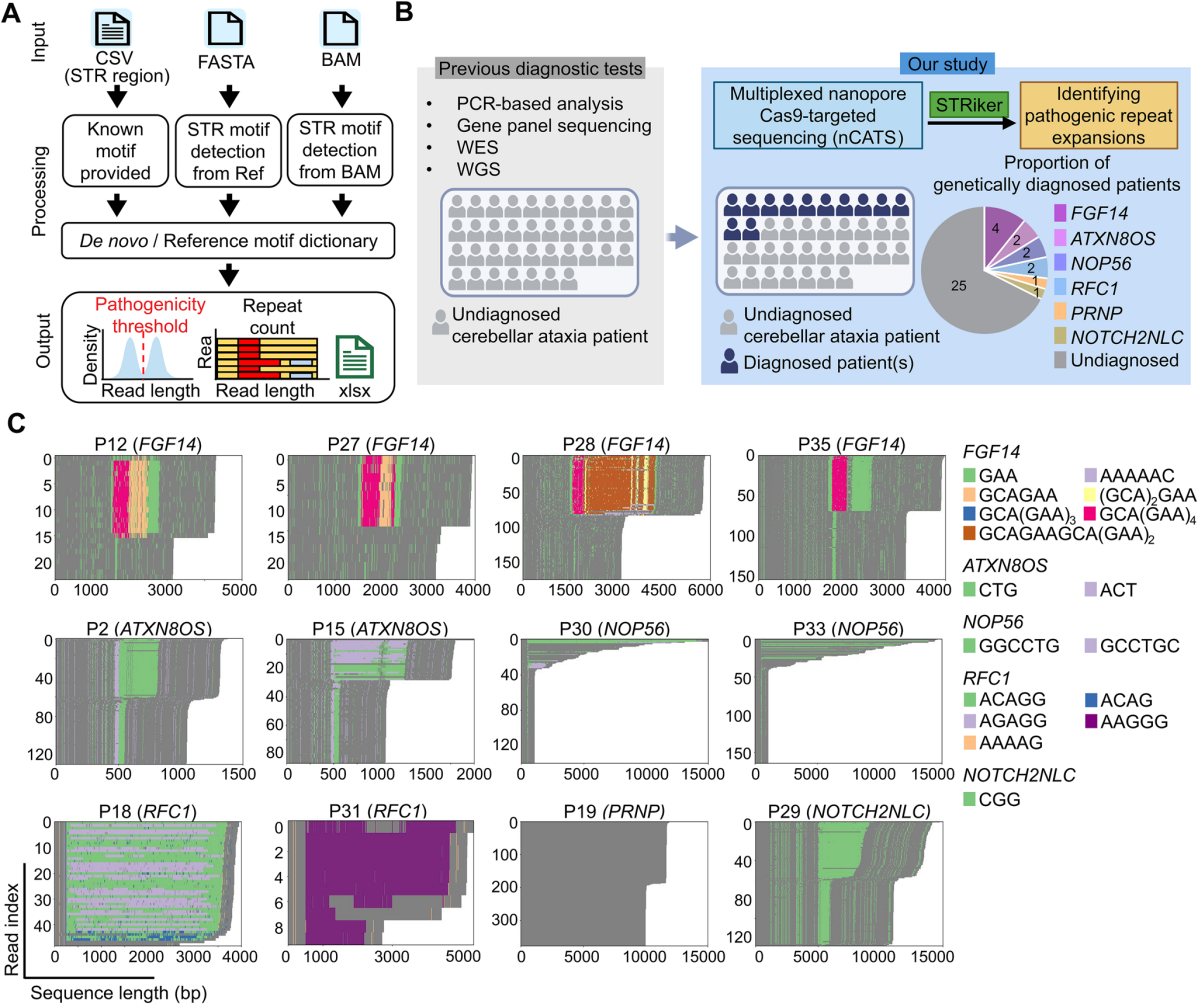
STRiker analysis pipeline and representative visualization outputs. (A) Overview of the STRiker pipeline developed for motif detection and repeat quantification from nCATS sequencing data. Using a BAM file and a target locus file as input, STRiker identified both known (reference) and novel (*de novo*) repeat motifs and generated output files that include a length distribution plot and a motif heatmap. (B) Diagnostic summary of 37 genetically undiagnosed patients with cerebellar ataxia. A total of 12 patients were newly diagnosed with pathogenic STR expansions, corresponding to a diagnostic yield of 32.4%. Created with BioRender.com. (C) Heatmap of repeat motifs in newly diagnosed patients identified through the STRiker pipeline. Different colors indicate different motifs within the same gene. *PRNP* repeat expansions comprise an octapeptide motif (PHGGGWGQ) of 24 bp and are, therefore, displayed without motif color differentiation. Ref, reference sequence used for motif detection; WGS, whole‐genome sequencing; WES, whole‐exome sequencing.

Notably, determining the sequence context and size of the STR regions remained challenging. Short‐read‐based programs, such as ExpansionHunter and GangSTR, rely on previously reported motifs [16, 17], whereas long‐read‐based tools, including Straglr and HMMSTR, enable more accurate detection of repeat expansions [6, 18]. Thus, this study developed (i) a dedicated analysis algorithm, named STRiker (https://github.com/BaeLab/STRiker), for detecting STR expansions from nanopore sequencing data, and (ii) a user‐friendly pipeline that identifies novel repeat motifs, detects interruption patterns, and automatically generates diagnostic reports for streamlined interpretation (Figure 3A). STRiker can identify both reference and *de novo* motifs directly from sequencing reads. In STRiker, any sequence unit observed in tandem in the reference or input reads ≥three times is automatically defined as a candidate STR motif. To account for rotational symmetry, motifs that could be transformed into each other through rotation were treated as a single motif. This approach enables the discovery of previously unreported motifs while maintaining consistency with known pathogenic variants.

The STRiker analysis pipeline integrates three input sources to generate comprehensive STR profiles (Figure 3A). The tool accepts STR region coordinates, along with optional known pathogenic motifs (CSV format), a reference genome (FASTA format), and aligned sequencing data (BAM format). STRiker detects motifs in both the reference genome and sequencing reads, producing a unified reference/*de novo* motif count report file. STRiker generates two primary outputs: (i) visualizations, including repeat‐count distributions that clearly delineate normal from pathogenic expansions and heatmaps showing motif patterns across individual reads; (ii) an Excel file (xlsx format) containing detailed motif composition data, including both reference/*de novo* motifs and their respective repeat counts. In the output file (PDF format), each gene‐specific figure includes a heatmap illustrating the distribution of repeat motifs across individual reads, with distinct colors for different motif types, and a read length density plot with Gaussian‐fitted peaks corresponding to normal and expanded alleles. The red vertical line indicates the pathogenic expansion threshold, allowing rapid visual distinction between normal and disease‐associated alleles.

### 2.5 Diagnostic Utility in Patients With Cerebellar Ataxia

Building on the experimental protocol and analysis algorithm, we applied the optimized nCATS–STRiker workflow to 37 genetically undiagnosed patients with cerebellar ataxia (Table S1). Since these patients exhibited clear clinical symptoms of cerebellar ataxia, we first applied the ataxia STR panel to 30 patients and then used the full panel for seven additional patients. Notably, most patients (35 of 37) had previously undergone extensive genetic testing, including targeted PCR‐based fragment analysis (89.2%), gene panel sequencing (75.7%), whole‐exome sequencing (62.2%), and whole‐genome sequencing (18.9%).

Consequently, we identified 12 patients with STR expansions in *FGF14* (four patients), *ATXN8OS*, *RFC1*, and *NOP56* (two patients each), as well as *NOTCH2NLC* and *PRNP* (one patient each). The overall diagnostic rate was 32.4% (12 of 37 patients) (Figure 3B,C and Table S2). To assess whether HMMSTR could also capture the structural complexity observed at these loci, we analyzed the genotypes of STR loci identified by STRiker in patient samples using HMMSTR (Table S3). Of the 13 pathogenic expansions identified by STRiker, HMMSTR failed to call pathogenic expansions in four cases. In particular, HMMSTR could not detect pathogenic expansions at the *ATXN8OS* or *RFC1* loci in Patient 15. Both loci exhibited clear expansions of *de novo* motifs (CTA/CTG at *ATXN8OS*; non‐canonical motifs at *RFC1*) that fell outside the predefined motif set in HMMSTR (Figure S7). Collectively, the optimized nCATS–STRiker workflow enabled completion of the long diagnostic odyssey for these patients, with an average time from symptom onset to genetic diagnosis of 8.9 years (range, 1–32 years; median, 5.5 years).

Representative examples of STRiker‐generated visualizations are presented for each genetically diagnosed patient, including per‐read sequence alignments that depict the structure and motif composition of the expanded alleles, as well as density plots summarizing overall read‐length distributions (Figure 3C and Figure S8). These visualizations enable intuitive examination of allele structures, repeat‐length distributions, and sequence interruptions, providing information that was previously difficult to visualize using conventional methods. Such visualization‐based reporting enables clinicians and researchers to readily recognize the configuration and interruption patterns of pathogenic alleles, thereby improving interpretability and facilitating clinical application of nCATS‐based STR analysis.

Following the genetic diagnosis of each patient, we performed cascade screening in six families to further test relatives of patients who had already been diagnosed by our improved nCATS method. Additional results showed that four affected relatives were also diagnosed (two with *FGF14* and one each with *ATXN8OS* and *NOP56*) (Figure S9). Moreover, we identified two carriers with repeat expansions in *FGF14* and *PRNP*, respectively, who were either asymptomatic (without clinical symptoms) or presymptomatic (currently symptom‐free but expected to develop symptoms later). One carrier with the *FGF14* expansion was the father of Patient 28. While both the patient and her sister exhibited dystonia and gait disturbance in early childhood, the carrier father remained neurologically asymptomatic even in his 50s. The presymptomatic carrier was the cousin of Patient 19, who possessed a *PRNP* octapeptide repeat expansion. This carrier is currently in his late 20s and has no significant symptoms. However, since his mother developed symptoms in her 30s and passed away in her 40s, our research team is striving to identify preventive management strategies for this presymptomatic carrier.

### 2.6 Identification of Novel Patterns in Disease‐Related STRs

**FIGURE 4:**
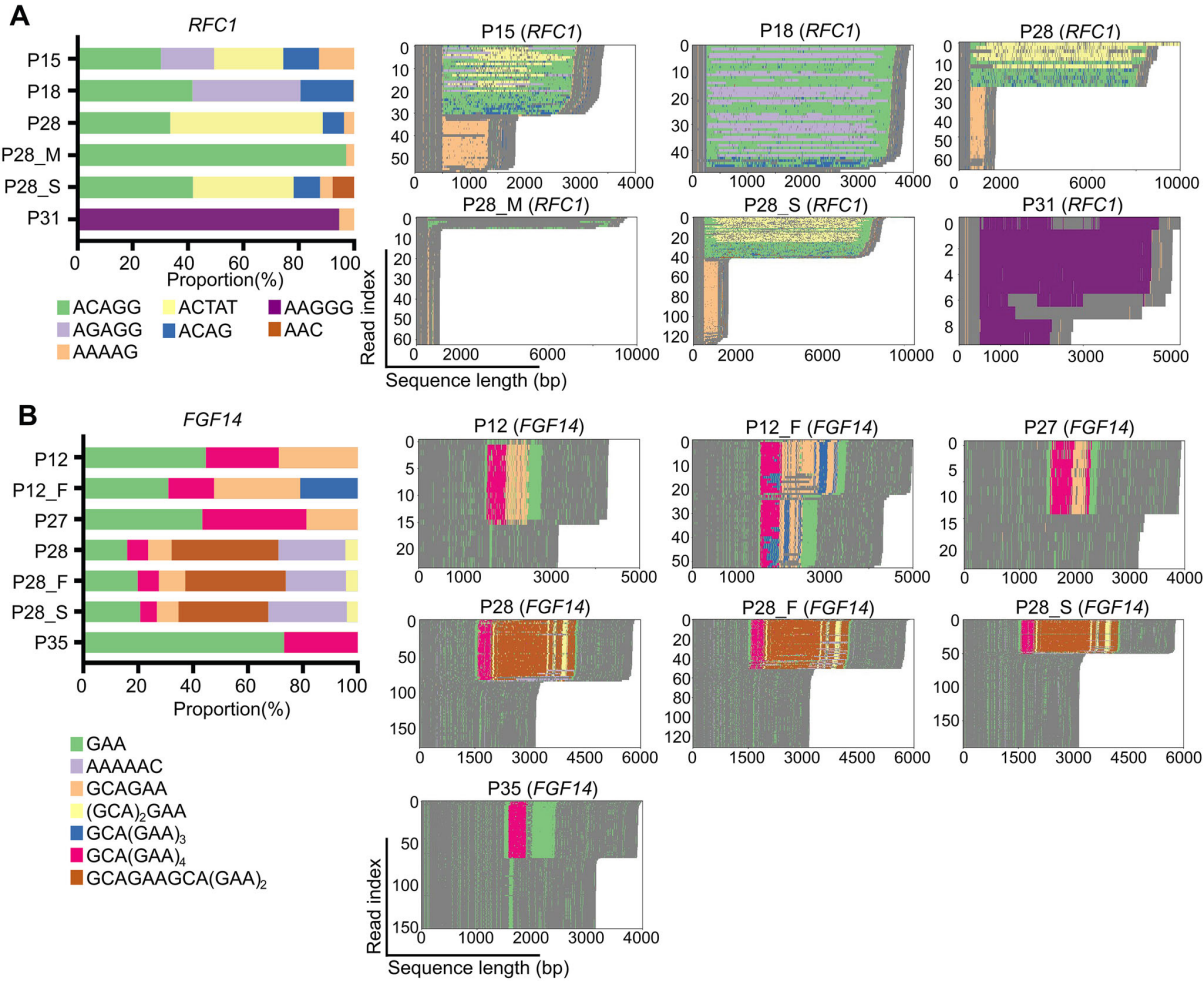
Analysis of STR motif composition and length distribution in *RFC1* and *FGF14* expansion carriers. (A, B) Stacked bar plots show the proportions of each repeat motif identified from nanopore sequencing reads, with distinct colors indicating different motif types. Heatmaps of the individual sequencing reads aligned to the repeat region visualize the arrangement and overall length of repeat units for each allele. (A) STRiker analysis of *RFC1* expansions revealed three distinct repeat motifs consistent with previously reported pathogenic configurations: ACAGG, AGAGG, and AAAAG. These results demonstrate that the STRiker pipeline reliably detects known pathogenic repeat structures. (B) *FGF14* expansions displayed variable combinations of GAA‐based motifs, including previously reported GAA, GAAGGA, and (GAA)_n_GCA configurations, as well as newly identified patterns, such as the AAAAAC expansion and a GCAGAAGCA(GAA)_2_ repeat motif. These findings illustrate that STRiker identified both canonical and novel repeat structures. Notably, multiplexed nCATS sequencing enabled the simultaneous detection of repeat expansions from different genes in a single diagnostic test, as in Patient 28, who carried heterozygous expansions in *RFC1* (carrier) and *FGF14* (pathogenic).

STRiker revealed novel STR patterns in specific ROIs from the sequencing data of 37 patients. Moreover, certain regions were observed to contain more than two distinct expansion motifs. For example, three distinct motifs (ACAGG, AGAGG, AAAAG) were identified for *RFC1* (Figure 4A), which aligns with previous reports [19, 20, 21]. Notably, repeat expansions with GAA and GAAGGA motifs have been reported for *FGF14*, as well as a (GAA)_n_GCA pattern [22, 23, 24, 25]. Furthermore, we identified additional novel expansions, including AAAAAC and a complex GCAGAAGCA(GAA)_2_ repeat motif in one of our pedigrees (Patient 28) (Figure 4B). Interestingly, the affected siblings with novel *FGF14* repeat motifs exhibited distinctive clinical features, including childhood onset, dystonia, spasticity, and T2 signal abnormalities in the globus pallidus, which are rarely observed in typical cerebellar ataxia (Table S2). These findings demonstrate a broader diversity of pathogenic repeat structures in *FGF14*‐related disorders than previously recognized. The presence of novel repeat motifs at other loci also warrants confirmation, emphasizing the need for further investigation into their potential clinical and biological significance.

### 2.7 Intergenerational Dynamics of Repeat Expansion and CpG Methylation

**FIGURE 5:**
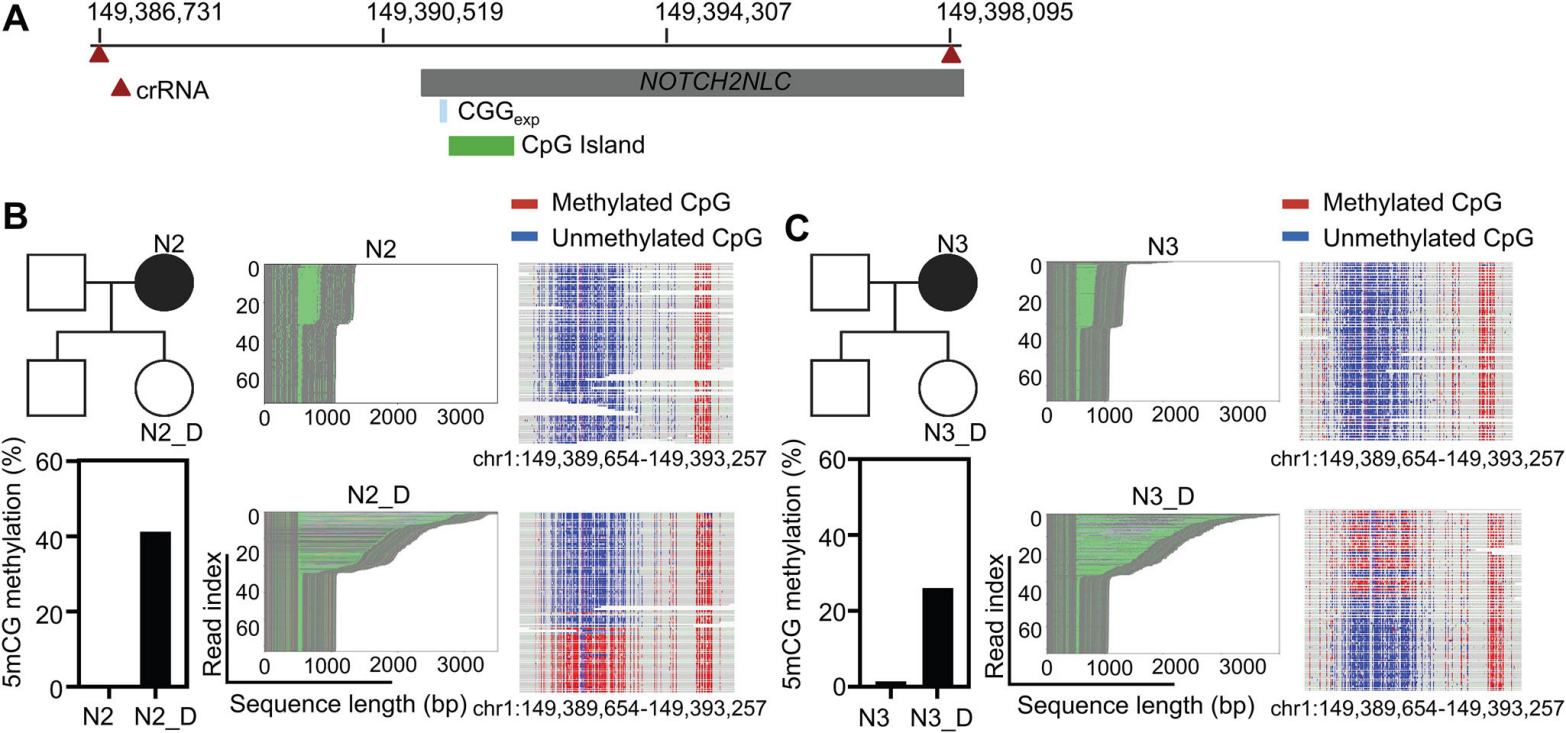
Intergenerational interplay between repeat expansions and CpG methylation at the *NOTCH2NLC* locus. (A) Schematic of the *NOTCH2NLC* locus with the expanded CGG repeat region (CGG_exp_), the upstream CpG island, and the targeted crRNA sites. CpG methylation states were obtained directly from native nCATS reads. Created with BioRender.com. (B, C) Two independent families with the expansion transmitted through maternal inheritance. In both cases, the mother (N) carried an unmethylated expanded allele and was affected, whereas the daughter (N_D) inherited a longer repeat with extensive methylation and remained asymptomatic. In each case, repeat structures are shown on the left, and CpG methylation states (methylated: red; unmethylated: blue) are shown on the right. Corresponding 5mCG methylation levels are summarized in the bar plots below each pedigree. Created with BioRender.com.

Furthermore, we investigated DNA methylation, which is crucial for determining whether a disease is actually occurring. The preservation of native DNA modifications by the optimized nCATS allowed us to extract CpG methylation information from the same reads. Although the multiplexed nCATS enables methylation profiling across all targeted loci, we specifically focused on the *NOTCH2NLC* locus, which is associated with neuronal intranuclear inclusion disease (NIID) (Figure 5A) [26, 27], given our previous findings on this disorder (Figure S10) [28]. We then analyzed methylation profiles of individuals in relation to repeat expansions at this locus.

Previous studies have described several cases of repeat contraction during paternal transmission, in which the fathers carried longer GGC repeat expansions but remained asymptomatic due to hypermethylation. However, the offspring of these fathers inherited alleles that were contracted relative to the paternal expansion yet still exhibited pathologically expanded traits and developed NIID—likely as a result of demethylation and loss of epigenetic suppression [26, 27, 28, 29, 30]. Notably, our previous study also identified an NIID family with the same pattern, in which both children were affected, whereas the father, who had hyperexpansion, remained asymptomatic (Figure S10). However, we observed a distinct pattern in our cohort, with expansion occurring during maternal transmission. In two unrelated families, the mothers carried expanded alleles without CpG methylation and were affected, whereas the offspring inherited even longer expansions but remained asymptomatic until the last follow‐up, at which the offspring were aged in their 40–50s; extensive methylation was also observed across the repeat‐flanking region (Figure 5B,C).

Together, these findings demonstrate a close association between repeat expansion length and methylation state, which may be important for predicting disease manifestation. In particular, these findings also suggest that repeat contraction or expansion across generations may vary depending on whether transmission is paternal or maternal.

### 2.8 Comparison of STRiker and Existing Tools Across Disease‐Associated STR Loci

To compare STRiker with RepeatHMM, Straglr, and HMMSTR, we analyzed Oxford Nanopore Technologies (ONT) reads from Patient 28 across 22 disease‐associated STR loci (Table S4). DeepRepeat [31] and STRique [32] were excluded because of the storage and runtime demands of signal‐level data [6]. Repeat‐count estimates were largely concordant, although discrepancies at some gene loci reflect the motif‐fitting design of STRiker. In *RFC1*, several motifs with abnormally high sigma values were detected due to heterogeneous repeat configurations; meanwhile, in *FGF14*, quantification of a single motif was impossible due to a complex structure containing GAAGCA.

A direct comparison of motif units showed that STRiker consistently identified a small number of consistent motifs (Table S5), whereas Straglr frequently split loci into several short and inconsistent units. For example, *ATXN2* was split into about 28 different units, *RFC1* into 26, and *C9orf72* into more than 20. Fragments as short as 2 bp (e.g., “GA” and “TA” in *RFC1*, which STRiker recovered as canonical ACAGG units instead) were also included. In *FGF14*, STRiker resolved the composite GAAGCA‐containing architecture, including GAA, GAAGCA, GAAGCAGCA, and GCAGAAGCA(GAA)_2_, whereas Straglr reported only “GAA.” These results indicate that while the total number of iterations may be numerically similar, only STRiker maintains an interpretable decomposition of the iteration structure.

We further benchmarked the performance of four tools using the general‐purpose datasets, CHM13 and NA12878. In the haploid CHM13 dataset (439 loci; Table S6) [33], all four tools showed overall agreement, as confirmed by pairwise concordance (Figure S11 and Table S7), a leave‐one‐out consensus comparison (Table S8), and Bland–Altman analysis (Figure S12 and Table S9). However, the concordance was substantially lower in the NA12878 dataset (Table S10) [34]: even two established sequence‐based callers, Straglr and HMMSTR, diverged by ≥twofold at approximately one‐quarter of evaluable loci (e.g., *XYLT1*: 8.5 for Straglr, but 104 for HMMSTR), and RepeatHMM failed to resolve half of the loci (27/54) despite sufficient sequence coverage.

**FIGURE 6:**
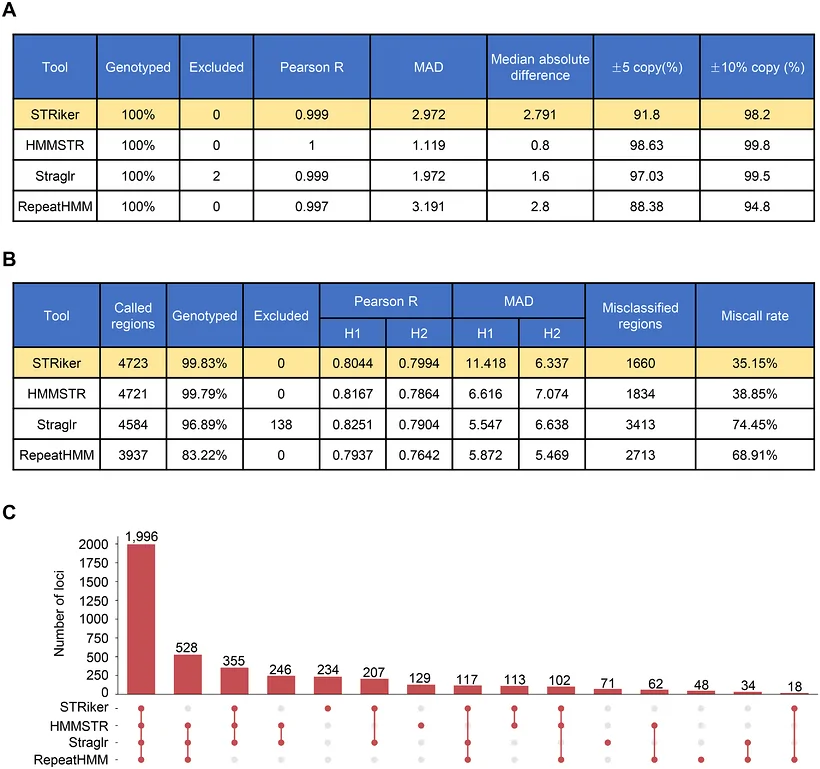
Benchmarking of STRiker against HMMSTR, Straglr, and RepeatHMM on CHM13 and NA12878. (A) Genotyping accuracy statistics for STRiker, HMMSTR, Straglr, and RepeatHMM against repeat counts reported for 439 CHM13 loci by Fang et al. [31], including the percentage of queried regions with non‐null genotypes returned and the proportion of calls within ± 5 copies and within ± 10% of the reported value. (B) Genotyping accuracy statistics for the same four tools against a haplotype‐resolved heterozygous NA12878 benchmark set (*n* = 4,731 loci). Pearson correlation and mean absolute difference are reported separately for the smaller (H1) and larger (H2) alleles. Miscall rate was counted over the intersection of loci genotyped by all four tools and expressed as a percentage of total calls for each tool. (C) UpSet plot of loci genotyped correctly by each tool from the heterozygous NA12878 benchmark set in (B), defined as loci where both the H1 and H2 calls fell within a data‐derived tolerance of ± 8 copies of the truth. Bars indicate the number of loci in each intersection. Filled circles below each bar denote which tool(s) contributed to that intersection, with connecting lines linking co‐occurring tools.

To further analyze this divergence across tools, we performed a comprehensive, genome‐wide, haplotype‐resolved benchmark of all four tools against CHM13 and NA12878, following the same benchmarking design used to validate HMMSTR [6]. For CHM13, we computed the Pearson correlation, mean and median absolute differences, and the proportions of calls within ± 5 copies and ± 10% of the truth for each tool (Figure 6A). All four tools showed high correlation with the truth (R > 0.99 for STRiker, Straglr, and RepeatHMM; R ≈ 1.00 for HMMSTR). However, the mean absolute difference for STRiker (2.97 copies) was 1.8 copies higher than that observed for HMMSTR (1.12), and its median absolute difference (2.79) was comparable to those of RepeatHMM (the highest among the four tools), making the accuracy for STRiker the second‐lowest of the four. Despite this, 91.8% of STRiker calls fell within ± 5 copies of the truth and 98.2% within ± 10%, indicating a modest, largely consistent underestimation across most loci rather than a small number of large failures.

Since NA12878 includes both homozygous and heterozygous loci, we constructed a haplotype‐resolved ground truth by independently mapping short flanking sequences onto each phased HGSVC assembly haplotype and determining copy number with Tandem Repeats Finder, yielding 13 860 chromosome 1 homozygous and 4731 heterozygous benchmark loci. On the homozygous set, STRiker, HMMSTR, and Straglr each genotyped >94% of loci with comparable correlation to the truth (R = 0.81–0.88), whereas RepeatHMM genotyped only 54.1% (Figure S13). On the heterozygous set, all four tools showed broadly similar correlation (R = 0.76–0.83). However, the estimate of the smaller allele (H1) by STRiker had a higher mean absolute difference (11.4 copies) than the other three tools (5.5–6.6 copies), whereas its estimate of the larger allele (H2) did not (6.3 copies; Figure 6B). Since STRiker does not natively separate alleles, we approximated H1 and H2 as the minimum and maximum per‐read repeat count at each locus. We separately evaluated the zygosity call for each tool (for STRiker, the number of KDE‐detected peaks in the per‐read repeat‐count distribution at each locus) against the true zygosity of all 18,591 NA12878 loci (Figure 6B). On the heterozygous set, STRiker had the lowest miscall rate of the four tools (35.2% vs. 38.9% for HMMSTR, 74.5% for Straglr, and 68.9% for RepeatHMM), meaning it was the least likely to collapse a true heterozygous locus into a single, homozygous‐looking call, an error that affected the majority of true heterozygous loci for Straglr and RepeatHMM. This came at the cost of the highest homozygous miscall rate among the four tools (28.4%, vs. 2.2% for Straglr, 7.0% for HMMSTR, and 19.7% for RepeatHMM), because the native peak‐finding for STRiker, unlike the clustering procedures used by HMMSTR and Straglr, does not include a model‐selection step to prevent spurious splitting of the read distribution of a truly homozygous locus into two peaks. UpSet plot of loci genotyped correctly within a data‐derived tolerance of the truth showed that STRiker uniquely recovered 341 homozygous and 234 heterozygous loci that all three other tools missed or called incorrectly (Figure 6C and Figure S14). Together, these results suggest that the genotyping performance of STRiker is complementary to, rather than better than, existing tools, with a specific limitation in distinguishing homozygous from heterozygous loci.

In addition to genotyping accuracy, STRiker demonstrated favorable computational performance relative to HMMSTR, the most resource‐intensive tool in this comparison. On CHM13, STRiker completed genotyping 7.4‐fold faster (1154 s vs. 8511 s) and used 6.1‐fold less peak memory (2.5 GB vs. 15.4 GB) with 16 threads; on the 54‐locus NA12878 panel, STRiker ran 9.4‐fold faster (23.8 vs. 222.9 s) and used approximately 47‐fold less memory (330 MB vs. 15.6 GB) (Tables S11 and S12). RepeatHMM had the lowest single‐thread resource usage but does not support multithreading and failed to genotype approximately half of the NA12878 loci; Straglr had the shortest overall wall clock time but has been reported to reliably phase heterozygous alleles only when they differ in size by more than 100 bp.

### 2.9 Summary of the Optimized nCATS–STRiker Workflow

**FIGURE 7:**
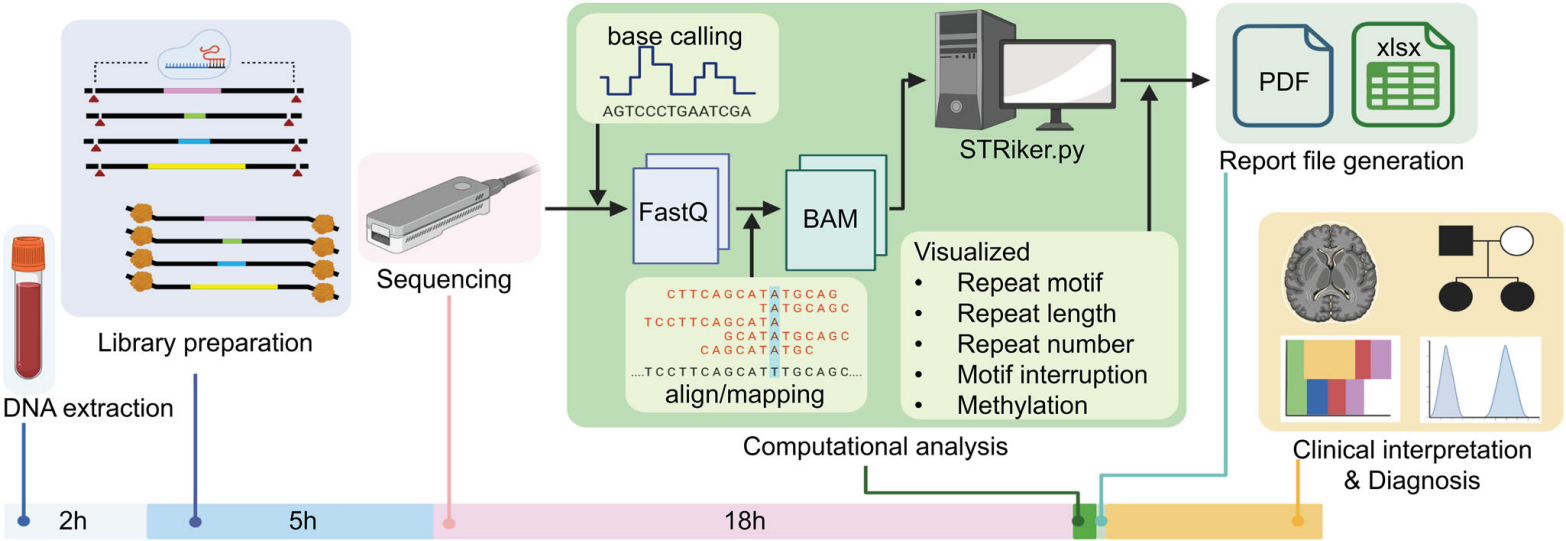
Schematic overview of the nCATS‐based diagnostic workflow for STR expansion disorders.Sample processing and sequencing workflow. Genomic DNA from patient blood samples was processed using nCATS for 56 STR‐associated loci. After multiplexed nanopore sequencing, raw data were base‐called and aligned, and STRiker was used to analyze repeat structures and lengths. The pipeline generates a visualized report (PDF, Excel) to support clinical interpretation. The total turnaround time from DNA extraction to data analysis was approximately 25 h (∼2 h for DNA extraction, 5 h for library preparation, 18 h for sequencing), with subsequent computational analysis completed within several minutes. Created with BioRender.com.

Our optimized nCATS method integrates streamlined library preparation, multiplexed Cas9‐targeted nanopore sequencing, and STRiker analysis to enable rapid and comprehensive detection of STR expansions (Figure 7). The total time from patient‐derived gDNA extraction to sequencing completion was approximately 25 h (∼2 h for DNA extraction, 5 h for library preparation, and 18 h for sequencing). Subsequent computational analysis with STRiker was completed in several minutes. This represents a marked reduction in diagnostic time compared with conventional workflows, in which PCR‐based fragment analysis and NGS panel testing are performed sequentially or in parallel, often requiring two to six months from sample submission to final reporting in a clinical setting. In contrast, our approach enables complete STR analysis within one to 2 days, substantially improving diagnostic efficiency.

## 3 Discussion

This study utilized the nCATS method, which is based on an amplification‐free, long‐read nanopore sequencing platform, to provide a comprehensive analysis of STR expansions. Accordingly, we selected gRNAs with high in vitro activity, adjusted cleavage lengths to approximately 10 kb to mitigate the length bias of nanopore sequencing, and employed adaptive sampling and methylation analysis. We also developed a dedicated algorithm, STRiker, to identify unbiased repeat motifs. Ultimately, we performed a simultaneous assessment of all currently defined STR loci (i.e., 56 sites) using a single test, with the gDNA extracted from patient peripheral blood. Compared with existing nCATS implementations, the 56‐locus panel used in this study is among the largest panels directly applied to patient‐derived peripheral blood in a clinical cohort. This study contributes to bridging the gap between panel scope and clinical applicability in existing implementations.

In addition, STRiker analyzed both reference and *de novo* motifs within repeat tracts and provided intuitive per‐read visualizations of repetitive sequence structures. Through STRiker, we identified complex *FGF14* repeat architectures, including the GCAGAAGCA(GAA)_2_ composite motif. Benchmarking the results against other tools, such as Straglr and HMMSTR, demonstrated that STRiker achieved comparable genotyping accuracy. Notably, STRiker uniquely recovered the largest number of correctly genotyped loci that were missed or miscalled by all three other tools (341 of 13 860 homozygous and 234 of 4731 heterozygous loci) and achieved the lowest heterozygous miscall rate among the four tools (35.2%, vs. 38.9%–74.5% for HMMSTR, Straglr, and RepeatHMM), indicating that STRiker can resolve a meaningful subset of loci that existing tools fail to capture. Consistent with these data, no single tool outperformed the others across every metric evaluated. Rather than competing primarily on raw genotyping accuracy, STRiker was designed to decompose repeat tracts into their constituent motifs and provide per‐read visualizations of repeat expansion patterns, capabilities not provided by repeat‐count‐focused tools, including HMMSTR and Straglr. For applications that also require precise allele separation and a single representative genotype per allele, we recommend using STRiker in conjunction with such tools. Importantly, some pathogenic expansions identified in this study were detected through *de novo* motif analysis and would have been missed by approaches relying solely on predefined motif models. This highlights the importance of performing reference and *de novo* motif analyses simultaneously.

Cerebellar ataxia comprises a group of disorders caused by mutations in various genes, genetic variants, and different inheritance patterns. While some patients can be diagnosed through short‐read NGS technologies, a substantial proportion remains undiagnosed, especially in adult‐onset cases. Using the optimized nCATS method, we newly identified pathogenic repeat expansions in 12 patients (32.4%) among 37 with cerebellar ataxia who remained genetically undiagnosed despite extensive prior genetic testing. Notably, *FGF14* expansions were identified in over 10% of patients (4 of 37), suggesting that this locus may be underrecognized in clinical settings. Repeat expansions in *ATXN8OS*, *NOP56*, and *RFC1* were also identified in multiple individuals, highlighting that loci often missed by conventional approaches can now be more reliably screened using long‐read sequencing. Furthermore, the *PRNP* octapeptide repeat expansion identified in our cohort represents the first reported case in an Asian population. Nonetheless, further studies are warranted to determine the proportion of undiagnosed cases attributable to each of these loci.

In addition, cascade screening of diagnosed families identified family members carrying pathogenic expanded alleles in five families, including two individuals who were asymptomatic or presymptomatic carriers. Such family‐based testing enables early diagnosis in at‐risk relatives and supports informed reproductive planning, including the use of preimplantation genetic testing, to allow the birth of unaffected offspring. Our observations further demonstrate that CpG methylation can mitigate the pathogenic effects of repeat expansions, suggesting that epigenetic protection may operate during both contraction and expansion events across generations. In particular, although previous studies reported that pathogenic alleles in the *NOTCH2NLC* gene can arise through contraction following paternal transmission, our study identified the opposite pattern in two unrelated pedigrees, in which maternally inherited alleles underwent expansion, and the STR region became hypermethylated in the offspring. Meanwhile, whether STR changes differ depending on maternal or paternal inheritance remains to be investigated; however, these findings highlight the importance of simultaneously profiling repeat length and methylation status to accurately assess disease risk in STR‐associated disorders, even in familial cases.

To further assess the pathogenic relevance of the novel *FGF14* repeat motifs, AAAAAC and GCAGAAGCA(GAA)_2_, identified in this study, we screened our long‐read sequencing database comprising approximately 80 PacBio and 70 nanopore samples harboring the *FGF14* repeat region. Overall, neither motif was detected in these 150 control samples, indicating that these variants are rare in our study population. This observation is consistent with a recent Japanese nanopore‐based study, which reported that the AAAAAC motif was found in one patient with cerebellar ataxia, but not in 59 healthy controls [35]. Similarly, although the exact GCAGAAGCA(GAA)_2_ motif has not been previously reported, several structurally related mixed GCA/GAA motifs, including GAAGCA, (GAA)_3_GCA, (GAA)_4_GCA, and GAA(GCA)_2_, have been described in patients with cerebellar ataxia [36]; these motifs were also observed in our cohort. Collectively, these population‐based and literature‐based comparisons provide additional, albeit indirect evidence of an association between these novel recurring motifs and disease.

However, our study has several potential limitations. First, despite the indirect evidence, functional validation (e.g., in vitro or in vivo modeling) of the pathogenicity of the novel repeat motifs identified in this study was not performed. As more patients are diagnosed using our platform, we anticipate that the clinical relevance of these novel motifs will become clearer. Recent reports have described that some individuals with *FGF14* expansions exhibit alternative phenotypes, such as Parkinson's disease [37], raising the possibility that distinct repeat motifs may contribute to phenotypic variability. Supporting this, patients in our cohort who harbored novel *FGF14* repeat motifs also exhibited atypical clinical presentations. Second, in asymptomatic individuals carrying hypermethylated expanded alleles, although we hypothesized that methylation‐mediated suppression occurs, age‐dependent penetrance cannot be ruled out. As our diagnostic platform enables the identification of previously undiagnosed repeat expansion disorders, accumulating additional cases will help improve our understanding of these mechanisms. Third, our benchmarking also revealed specific limitations in the genotyping algorithm within STRiker. Moreover, the native zygosity calling in STRiker, which relies on kernel density estimation (KDE) of the per‐read repeat‐count distribution without a model selection step such as the Bayesian information criterion (BIC), most frequently misclassified truly homozygous loci as heterozygous among the four tools benchmarked (miscall rate 28.4%), reflecting a tendency to over‐split unimodal read distributions into spurious peaks. Moreover, incorporating a BIC‐based model‐selection step into this peak‐finding procedure represents a concrete direction for improving zygosity calling in future versions of STRiker. In addition, because STRiker does not natively separate alleles, we approximated the smaller and larger allele using the minimum and maximum per‐read repeat count for this benchmark, and this approximation yielded a notably higher mean absolute difference for the smaller allele (H1: 11.4 copies) than the other three tools (5.5–6.6 copies). Despite these limitations, we believe that this approach can contribute to elucidating the genotypic and phenotypic diversity of repeat expansion disorders, similar to the role that whole‐exome sequencing has played in advancing our understanding of many rare genetic diseases.

Collectively, our results demonstrate the clinical utility of long‐read, amplification‐free STR sequencing in the diagnosis of neurogenetic disorders. The optimized nCATS platform, featuring an intuitive visualization tool, provides a robust and scalable solution for the accurate detection and interpretation of repeat expansions. As new disease‐associated STR loci are continually discovered and curated, our platform will be actively updated to incorporate these loci, ensuring that diagnostic coverage remains comprehensive and current. Indeed, the ability of this platform to simultaneously screen multiple STR loci holds promise for both improving diagnostic outcomes and deepening our understanding of disease pathogenesis in cerebellar ataxia and other repeat‐associated diseases. In addition to improving diagnoses, recent advances in genome editing strategies, including prime editing, may eventually enable direct correction of pathogenic repeat expansions, further emphasizing the value of accurately resolving repeat structures and motif contexts, as demonstrated previously [38].

## 4 Materials and Methods

### 4.1 Study Design

This study was a single‐center, retrospective observational study that also included methodological development of the nCATS platform and a dedicated analysis pipeline, STRiker, for comprehensive detection of disease‐associated STR expansions. The work comprised three components: (i) optimization of the nCATS experimental workflow, including guide RNA selection and library preparation conditions; (ii) development of the STRiker analysis pipeline for automated repeat‐length estimation, motif identification, and generation of user‐friendly visual reports; (iii) application of the finalized nCATS–STRiker workflow to a diagnostic cohort of 37 patients with cerebellar ataxia, followed by cascade screening of available family members to characterize inheritance patterns and intergenerational changes in repeat length and CpG methylation.

The clinical component of the study used retrospectively collected gDNA and clinical information from patients and relatives who met the eligibility criteria outlined in the “Study participants” section. The optimized nCATS–STRiker workflow was applied to all available samples using either an ataxia or a comprehensive STR panel. Results were interpreted with reference to established pathogenic repeat‐size thresholds, methylation profiles, and individual clinical phenotypes. The sample size was determined by the availability of eligible samples during the study period and was considered sufficient for methodological optimization and estimating diagnostic yield.

The primary endpoint was the diagnostic yield of the nCATS–STRiker workflow, defined as the proportion of previously undiagnosed patients with cerebellar ataxia in whom a pathogenic or likely pathogenic STR expansion was identified. Secondary objectives included evaluating on‐target sequencing performance (total on‐target bases, length‐normalized coverage depth, and per‐locus coverage uniformity), characterizing known and novel STR motifs and interruption patterns across 56 loci, assessing intergenerational dynamics of repeat length and CpG methylation, with a particular focus on *NOTCH2NLC*, and benchmarking STRiker runtime performance on nanopore datasets.

### 4.2 Study Participants

Participants were retrospectively enrolled from Seoul National University Hospital (SNUH) based on the following eligibility criteria: (1) Korean patients who visited the Department of Neurology at SNUH; (2) patients suspected of having cerebellar ataxia, with evidence of cerebellar atrophy on brain imaging or neurological symptoms indicative of cerebellar dysfunction, such as ataxia, dysarthria, and dysmetria; (3) patients who remained genetically undiagnosed despite prior testing. Only patients meeting all these criteria were selected for further evaluation. A comprehensive retrospective review of medical records was conducted, and for patients with a family history, samples from affected family members were also collected when available. The study protocol was approved by the Institutional Review Board of Seoul National University Hospital (2004‐042‐1116 & 2402‐149‐1518) and was conducted in accordance with relevant guidelines and regulations.

### 4.3 Cell Line Culture Conditions

HeLa (ATCC, CLL‐2) cells were cultured in Dulbecco's modified Eagle medium (DMEM) (Welgene, KR; cat. LM001‐05) supplemented with 10% fetal bovine serum (FBS) (Welgene; cat. PK004) and 1% antibiotics (Welgene; cat. LS203‐1) in a humidified incubator at 37°C and 5% CO_2_.

### 4.4 Design and Optimization of gRNA for nCATS in Disease‐Related STR Regions

We selected genes associated with STR‐related disorders and designed gRNAs to target specific ROIs for nCATS. Our previous research demonstrated that, during nanopore sequencing, shorter DNA fragments are preferentially sequenced compared to longer fragments [8, 9]. To address this, we designed gRNAs approximately 5000 bp upstream and downstream of each side of the repeat regions to generate DNA fragments of approximately 10 kb. This design ensures that long DNA fragments containing repeat expansions are sequenced without bias against other genomic regions, enabling equitable representation of the 56 target genes.

To optimize multiplexed gene sequencing using nCATS, we designed gRNAs using Cas‐Designer, which identifies potential off‐target sites by searching for genomic sequences that differ by up to two nucleotides [39, 40, 41]. We prioritized gRNAs that presented a single perfect on‐target site and no predicted off‐targets with mismatches of one or two bases, thereby ensuring high target specificity across nearly all target loci. We concluded that cleavage efficiency is the critical factor and finalized one pair of gRNAs for each ROI. We added a second pair of gRNAs to enhance coverage for five genes (*ATXN8OS*, *TBP*, *DAB1*, *NUTM2B‐AS1*, and *DIP2B*) that had insufficient sequencing depth.

### 4.5 Synthesis of gRNAs by In Vitro Transcription

CRISPR RGEN tools (Cas‐Designer) were used to design the gRNA sequences [39, 40]. A list of oligos for the target sequences is provided in Tables S13 and S14. The gRNA template oligos were ordered from Macrogen and Cosmogenetech. The gRNAs were synthesized by in vitro transcription using T7 RNA polymerase (NEB) and template oligonucleotides. The gRNA product was then purified using the RNeasy mini kit (Qiagen) and quantified using a NanoDrop.

### 4.6 In Vitro Cleavage Assay

*Streptococcus pyogenes* Cas9 (SpCas9) nuclease was obtained from Enzynomics (Daejeon, Korea). To generate Cas9 RNP complexes, SpCas9 and gRNA were mixed at a 3:5 ratio and incubated at room temperature for 30 min. For the IVC assay, target regions encompassing the gRNA binding sites were first amplified from HeLa gDNA by PCR using the primers listed in Table S15. The PCR products were gel‐purified and incubated with Cas9 RNP complexes at 37°C for 10 min. The reaction mixtures were loaded onto 1% Tris–borate–EDTA (TBE) agarose gels, and gel images were captured using a Gel Doc system. Band intensities were quantified in ImageJ, corrected for fragment length, and used to calculate cleavage efficiency. Band intensities were normalized by dividing each value by the corresponding product length.

$$\begin{array}{c} \frac{\begin{array}{c} \mathrm{cut}\;\mathrm{band}\;\left(1\right)\;\mathrm{intensity}\;\left(\mathrm{normalized}\right)\\ +\;\mathrm{cut}\;\mathrm{band}\;\left(2\right)\;\mathrm{intensity}\;\left(\mathrm{normalized}\right) \end{array}}{\begin{array}{c} \mathrm{uncut}\;\mathrm{band}\;\;\mathrm{intensity}\;\left(\mathrm{normalized}\right)+\mathrm{cut}\;\mathrm{band}\;\left(1\right)\;\mathrm{intensity}\;\\ \times \;\left(\mathrm{normalized}\right)+\mathrm{cut}\;\mathrm{band}\;\left(2\right)\;\mathrm{intensity}\;\left(\mathrm{normalized}\right)\; \end{array}}\times 100 \end{array}$$

### 4.7 Library Preparation and Cas9‐Mediated Nanopore Long‐Read Sequencing

Genomic DNA was extracted from whole blood samples using the Qiagen DNeasy blood and tissue kit (Qiagen; cat. 69504). The crRNAs and tracrRNAs were ordered from Integrated DNA Technologies (IDT; Coralville, Iowa, USA), and the Cas9 nuclease was purchased from Enzynomics and IDT. We prepared sequencing libraries from 10 µg of gDNA using the SQK‐LSK114 kit (Oxford Nanopore Technologies, UK) and a Cas9‐mediated targeted enrichment protocol [42]. To improve consistency and depth of on‐target sequencing coverage, we modified the standard ONT protocol by separating the Cas9 cleavage and dA‐tailing steps. While the original protocol performs both steps simultaneously, we removed the Cas9 protein via bead purification after cleavage, then performed dA‐tailing using the dA‐tailing module (NEB, E6053L) (Figure S4). Prepared libraries were loaded onto flow cells (R10.4.1) and sequenced on the MinION platform (Oxford Nanopore Technologies).

### 4.8 Coverage Analysis

Coverage depth for each ROI was calculated from the aligned BAM files as the number of mapped bases divided by the target length (bp). To evaluate the overall sequencing performance, we computed two metrics: (i) total on‐target bases, calculated by summing the product of coverage depth and ROI length across all targets using the following formula:

$$Total\;base=\sum_{i=1}^{N}\left(coverage\;depth_{i}\times ROI\;length_{i}\right)$$

where *N* denotes the number of ROIs included in the analysis. This represents the total number of aligned bases within the targeted regions and reflects the on‐target sequencing yield per condition. (ii) To account for differences in ROI size when comparing average sequencing depth across conditions, the length‐normalized average coverage depth was calculated as follows:

$$\begin{array}{ccc} & & Length-normalized\;average\;coverage\;depth\\ & & \;=\frac{\sum_{i=1}^{N}\left(coverage\;depth_{i}\times ROI\;length_{i}\right)}{\sum_{i=1}^{N}ROI\;length_{i}} \end{array}$$

This approach minimizes length‐dependent bias and reports the mean sequencing depth per target after normalization for ROI size. In contrast, for per‐locus comparisons, raw coverage depth values, without length normalization, were used to visualize coverage distributions across individual STR loci.

### 4.9 Preparation of Browser Extensible Data (BED) Files for Adaptive Sampling

To facilitate adaptive sampling, a gene panel was designed, and its corresponding BED file was created (Table S16). Given the potential variability in cleavage points at the terminal regions of the library DNA, which could affect alignment during adaptive sampling, the BED file was configured to extend 100 bp upstream and downstream of the cleavage points. This adjustment ensures that even if the cleavage points deviate slightly from the expected range, the corresponding library fragments will not be prematurely excluded during the adaptive sampling process, enabling accurate classification.

### 4.10 Analysis of nCATS Data and Visualization

Sequencing was performed using MinION R10.4.1 flow cells, and the resulting POD5 files were base‐called using the Dorado software (version 0.6.2) (https://github.com/nanoporetech/dorado). Subsequently, reads were aligned to the GRCh38 reference genome using minimap2 (version 2.28‐r1209) (https://github.com/lh3/minimap2) [43]. BAM files were then sorted and indexed using Samtools (version 1.18) (https://www.htslib.org/) [44].

Motif and methylation state analyses were conducted on reads within the ROIs using a known table of repeat expansion information (Table S17). It has been well‐established that the phenotype of diseases caused by repeat expansion can be influenced by the presence of non‐motif sequences between consecutive motifs, a phenomenon referred to as the “interruption effect” [45].

To address this, both the highest consecutive repeat count and the total repeat count were investigated. Due to the relatively high error rate of nanopore sequencing and the variation in repeat counts within samples, confirming that the observed interruption effect was not a false positive was essential. Thus, visualization techniques were employed to distinguish motifs from non‐motif sequences, thereby verifying the occurrence of interruptions.

### 4.11 STRiker Algorithm for STR Analysis

This study developed STRiker (https://github.com/BaeLab/STRiker), a dedicated algorithm and computational pipeline for comprehensive STR analysis of sequencing data. The algorithm accepts aligned reads (BAM format), a reference genome (FASTA), and STR region coordinates with known pathogenic motifs (CSV).

#### 4.11.1 Motif Discovery

STRiker employs dual motif discovery strategies. First, reference sequences with flanking regions were extracted and then scanned using a sliding window (3–30 bp) to identify tandem repeats that occurred ≥three times consecutively. Second, *de novo* motif discovery was performed directly from sequencing reads overlapping each STR region. To handle circular permutations inherent in tandem repeats, all motifs were initially canonicalized to their lexicographically smallest rotation. However, if a discovered motif matched any rotation of a known pathogenic motif, this motif was reassigned to the known motif form to maintain clinical relevance. For example, if CAG was the known pathogenic motif and AGC was discovered, then AGC was reported as CAG rather than AGC.

#### 4.11.2 Pattern Decomposition

Sequences were decomposed using a greedy algorithm that matches the longest motif at each position, considering all rotational variants. When multiple rotational variants are possible, preference was given to known pathogenic motifs. Consecutive identical motifs were grouped to simplify patterns. For example, a sequence containing CAGCAGCAGCAACAGCAG was represented as ((CAG, 3), (CAA, 1), (CAG, 2)), explicitly capturing interruption patterns that may modulate disease severity.

#### 4.11.3 Repeat Quantification

For each STR locus, repeat counts were calculated by summing occurrences of the primary pathogenic motif and its rotational equivalents across all reads. Only reads that fully spanned both boundaries of the STR region were included in the analysis to ensure accurate repeat counts. Kernel density estimation was used to visualize repeat‐length distributions, enabling detection of expanded alleles and mosaicism. Pathogenic expansions were identified by comparing observed repeat counts with established clinical thresholds.

#### 4.11.4 Quality Control

Quality metrics included coverage depth (minimum threshold of 30 × for confident variant calling), motif concordance between the reference and *de novo* discoveries, and pattern complexity assessment. The pipeline outputs were as follows: (1) an Excel file comparing reference and *de novo* motifs with the associated occurrence frequencies, (2) a PDF report with pattern visualizations and repeat distributions, and (3) coverage statistics for each analyzed locus.

Performance evaluation was conducted on a workstation equipped with an Intel Core i7‐13700K processor, 32 GB of RAM, and running Ubuntu 20.04. STRiker was executed using 4 CPU cores with multiprocessing enabled, with the following parameters: minimum motif length of 3 bp, maximum motif length of 30 bp, minimum consecutive motif count of 10, and minimum motif coverage of 5 × (Table S18). Processing time was measured using the Unix time command across three independent runs for each dataset, and real time (wall‐clock time) was recorded as the primary performance metric (Table S19).

### 4.12 Accuracy Benchmarking Against CHM13

To benchmark STRiker against existing long‐read STR genotyping tools (HMMSTR, Straglr, RepeatHMM, we first evaluated performance on the CHM13 cell line using publicly available ONT long‐read data (rel8, Guppy 5.0.7 basecalled, ∼120x coverage), downloaded from the T2T consortium repository (https://s3‐us‐west‐2.amazonaws.com/human‐pangenomics/index.html?prefix = T2T/CHM13/nanopore/rel8‐guppy‐5.0.7/) and aligned to the CHM13v2.0 reference. Following HMMSTR (Van Deynze et al., Nucleic Acids Research, 2025), we used the repeat counts reported in Table S1 of Fang et al. (2022) as ground truth for the same 439 loci. STRiker reports the copy‐number status of each motif within a repeat expansion rather than reducing a locus to a single repeat unit; for this benchmark, however, we followed the same convention used for the other tools and expressed the called expansion size as (aligned length increase) / (motif size), enabling a direct, motif‐length‐normalized comparison across tools.

### 4.13 Accuracy Benchmarking Against NA12878

Since NA12878 contains a mixture of homozygous and heterozygous loci, we first needed a haplotype‐resolved ground truth to separate the two. Following the described HMMSTR Methods approach, for each candidate locus we independently mapped 2 kb flanking sequences on each side (hg38 coordinates) to each phased HGSVC assembly haplotype using mappy (v2.31, preset = ‘asm5’), retaining only flank pairs that mapped uniquely (MAPQ 60) and in the same orientation as the assembly contig. The sequence between the resolved flank coordinates on each haplotype was then extracted and passed to Tandem Repeats Finder (TRF) to determine copy number, using parameters Match = 2, Mismatch = 7, Indel = 7, PM = 80, PI = 5, Minscore = 5, MaxPeriod = 500.

Of the 189 234 loci with both haplotypes resolved, 167 323 were homozygous, and 4731 were heterozygous under this definition. For computational tractability, we restricted the homozygous benchmark to chromosome 1 (13 860 loci), yielding a combined benchmark set of 18 591 NA12878 loci (13 860 homozygous and 4731 heterozygous).

Unlike the CHM13 benchmark set, which consists of long, high‐coverage loci, the NA12878 chr1 homozygous set consists of considerably shorter loci (median width 20 bp) with shorter motifs (33.3% ≤3 bp). Accordingly, we directly quantified the number of motif occurrences in each read rather than using the length‐based estimator applied to CHM13. We separately evaluated value‐calling accuracy and zygosity‐calling accuracy for STRiker, as these metrics measure different capabilities. For accuracy (Pearson R, MAD), because STRiker does not natively separate alleles, we assigned the minimum and maximum of the two per‐read motif‐count estimates to H1 and H2, respectively, for loci known to be heterozygous, and the mean per‐read count to both H1 and H2 for loci known to be homozygous. H1/H2 comparison sets for all tools followed the convention in the Methods for HMMSTR: for a truth‐homozygous locus, a tool reporting one allele contributes that value to both H1 and H2, while a tool reporting two alleles contributes the value closer to the truth to H2 and the farther value to H1; for a truth‐heterozygous locus, the two calls for each tool are sorted so H1 is the smaller and H2 is the larger. To determine which loci each tool genotyped correctly, a locus was scored as correct if both its H1 and H2 calls fell within a tolerance of the truth; this tolerance (± 4 copies for the homozygous set, ± 8 copies for the heterozygous set) was derived independently for each set from the empirical distribution of calling error using the Kneedle elbow‐detection method [46], rather than chosen arbitrarily. For zygosity, allele count for STRiker was determined via KDE of the per‐read motif‐count distribution, with the number of detected peaks giving the call. Following the Methods for HMMSTR, a truth‐homozygous locus was scored as misclassified if a tool reported more than one allele, and a truth‐heterozygous locus if it reported only one; misclassified regions were counted over the intersection of loci genotyped by all four tools, and the miscall rate was this count divided by the total number of called regions of each tool.

### 4.14 Statistical Analysis

Statistical analyses were primarily descriptive and focused on summarizing assay performance and clinical diagnostic yield rather than formal hypothesis testing. Continuous variables, such as age at onset, time from symptom onset to genetic diagnosis, and repeat counts, are summarized as the mean and standard deviation, the median, and the range, as appropriate; categorical variables, such as sex, presence of a family history, and presence of specific STR expansions, are summarized as counts and percentages.

For in vitro optimization experiments, normalized cleavage efficiencies were calculated from ImageJ‐quantified, length‐corrected band intensities, as described in the “IVC” section. Sequencing coverage metrics for each ROI, total on‐target bases, and length‐normalized average coverage depth were calculated from aligned BAM files as described in the “coverage analysis” section. Coverage uniformity across loci and experimental conditions was assessed by comparing distributions of raw and length‐normalized depth values rather than by formal hypothesis testing. STRiker‐generated repeat‐length distributions and motif compositions, including reference and *de novo* motifs, were analyzed using custom Python scripts integrated into the STRiker pipeline.

## Author Contributions

**S.B. and J.M**. conceived the project. **C.J**. developed the bioinformatics algorithms. **M.K. and N.K**. performed the cell and sequencing experiments. **S.L., C.J., and G.‐H.H**. analyzed the sequencing data. **S.L., S.‐T.L., K.C., S.K.L., H.‐J.K., J.H.C., and J.M**. enrolled and clinically evaluated the study patients. **S.B. and J.M**. supervised the project. **S.L., C.J., M.K., and S.B**. wrote the manuscript, with input from all authors.

## Funding

This research was supported by grants from the National Research Foundation of Korea (NRF) No. RS‐2024‐00344068 awarded to J.M. Additional support by grant of Korean ARPA‐H Project through the Korea Health Industry Development Institute (KHIDI) No. RS‐2026‐25618734, a grant from the Ministry of Food and Drug Safety (No. 25202MFDS003) in 2025, and the SNUH Lee Kun‐hee Child Cancer & Rare Disease Project (No. 25B‐001‐0700), also awarded to S.B.

## Conflicts of Interest

The authors declare that they have no competing interests.

## Supporting information




**Supporting File 1**: advs77475‐sup‐0001‐SuppMat.pdf.

**Supporting File 2**: advs77475‐sup‐0002‐SuppMat.docx.

**Supporting File 3**: advs77475‐sup‐0003‐SuppMat.xlsx

## Data Availability

All data are available in the main text or the supplementary materials. The code related to this study is available at https://github.com/BaeLab/STRiker.
