# Supplementary material for: Optimized Cas9‐Enriched Nanopore Sequencing and Analysis Workflow for Clinical Diagnosis of Repeat Expansion Disorders
Source: Adv Sci (Weinh). 2026 Aug 29:e77475. Online ahead of print. doi: 10.1002/advs.77475 (PMC13525614; doi:10.1002/advs.77475)
Supplement: Supplementary file 1 [file ADVS-9999-e77475-s002.pdf]

Supplementary Materials

Optimized Cas9-enriched nanopore sequencing and analysis workflow for clinical diagnosis of repeat expansion disorders

Seungbok Lee, Chanju Jung, Minjeong Kim, Narae Kim, Gue-Ho Hwang, Soon-Tae Lee, Kon Chu, Sang Kun Lee, Han-Joon Kim, Jong Hee Chae, Sangsu Bae, Jangsup Moon

List of Supplementary Materials:

Figure S1 to S12 for multiple supplementary figures

Table S1 to S19 for multiple supplementary tables

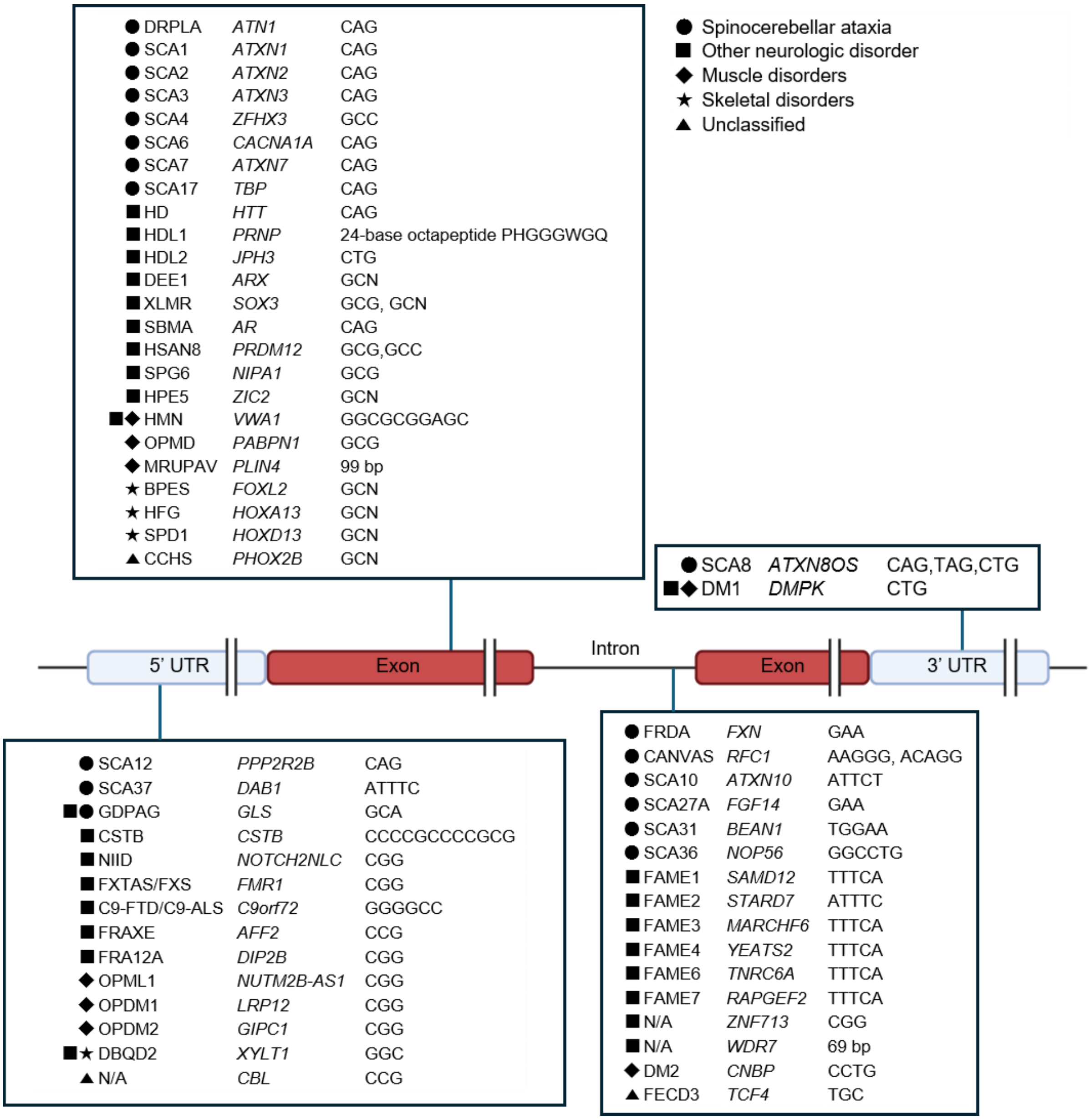

Figure S1. Overview of STR loci included in the 56-gene panel.

Schematic representation of disease-associated short tandem repeat (STR) loci categorized by their genomic location (5′ untranslated region (UTR), exon, intron, or 3′ UTR) and repeat motif type. Each box lists representative disorders, associated genes, and repeat motifs. Symbols indicate the major clinical categories. This panel comprises 56 known pathogenic or candidate STR loci encompassing a wide range of repeat motifs implicated in neurological, muscular, and developmental disorders. Created with BioRender.com

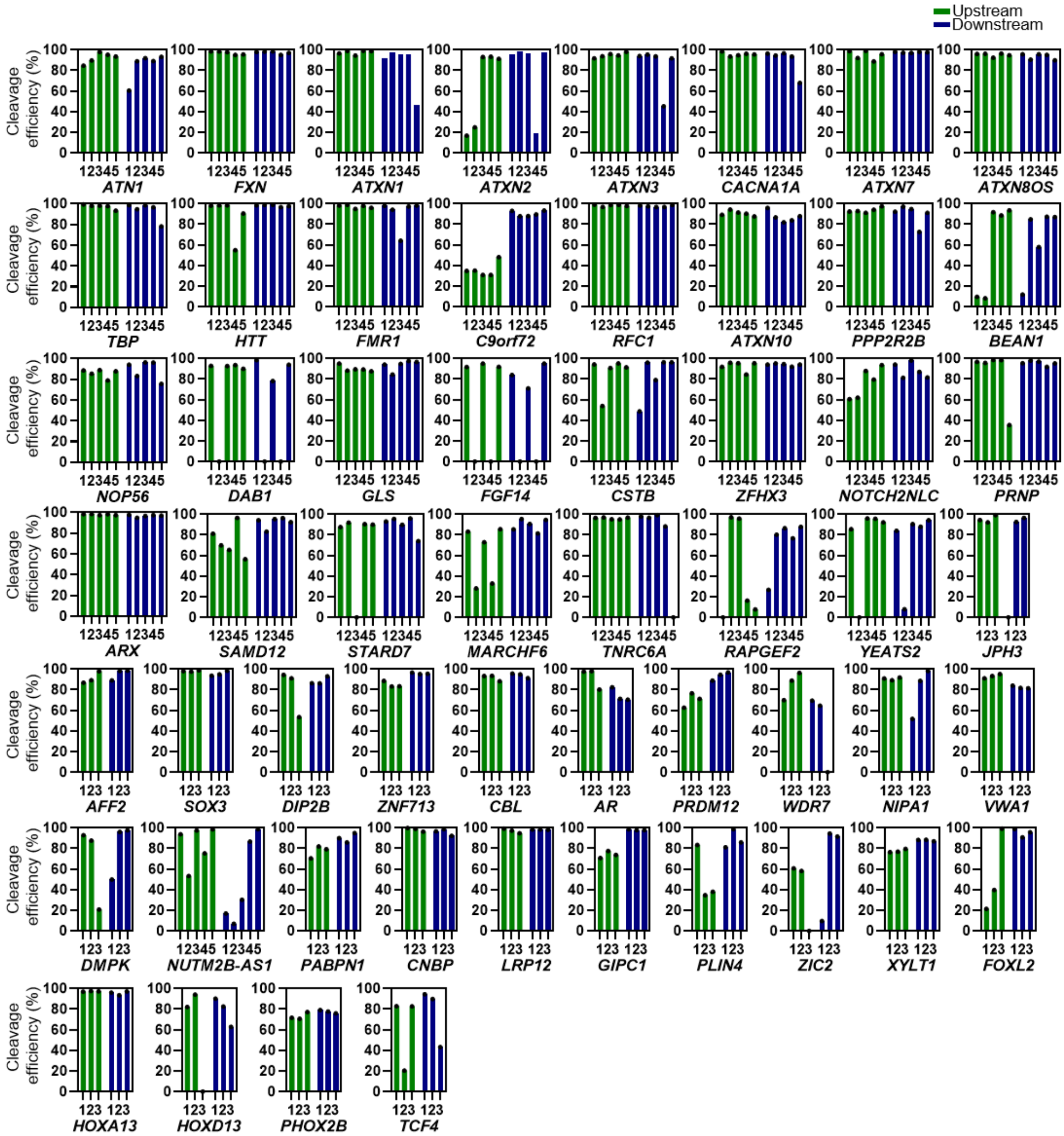

Figure S2. sgRNA screening for high cleavage efficiency via *in vitro* cleavage assay.

Cleavage efficiency of sgRNAs targeting upstream and downstream regions of repeat expansion disease-associated gene regions of interest (ROIs), assessed by *in vitro* cleavage assay (n=1). Three or five different sgRNAs were tested for each region.

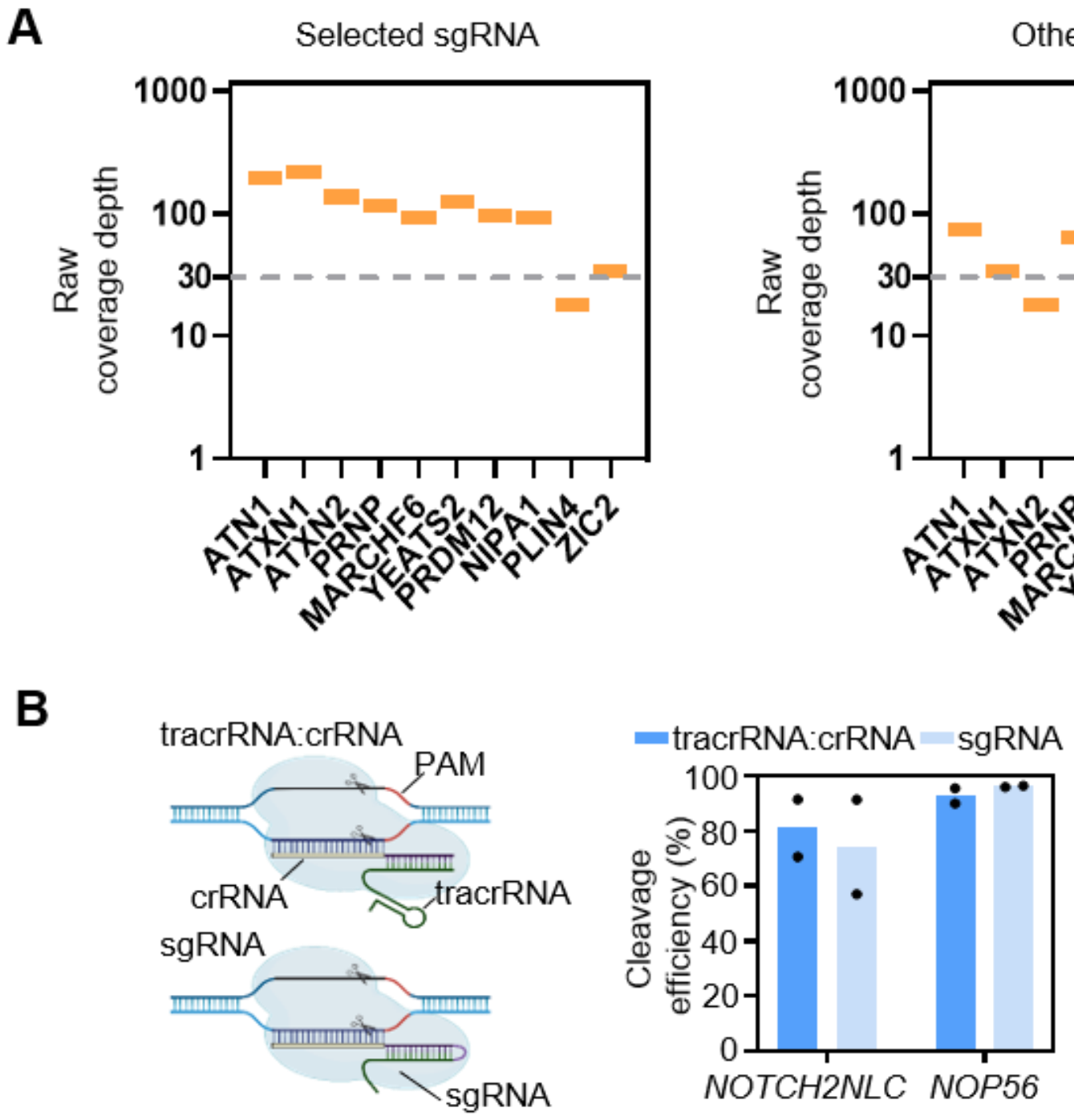

C

Item	sgRNA	crRNA:tracrRNA
Cost per guide	USD 109	USD 84
Shared tracrRNA (5 nmol)	–	USD 95
Number of guides (56-locus panel)	122	122
Total estimated cost	USD 13,298	USD 10,343
Cost reduction	–	22% (≈ USD 2,955)

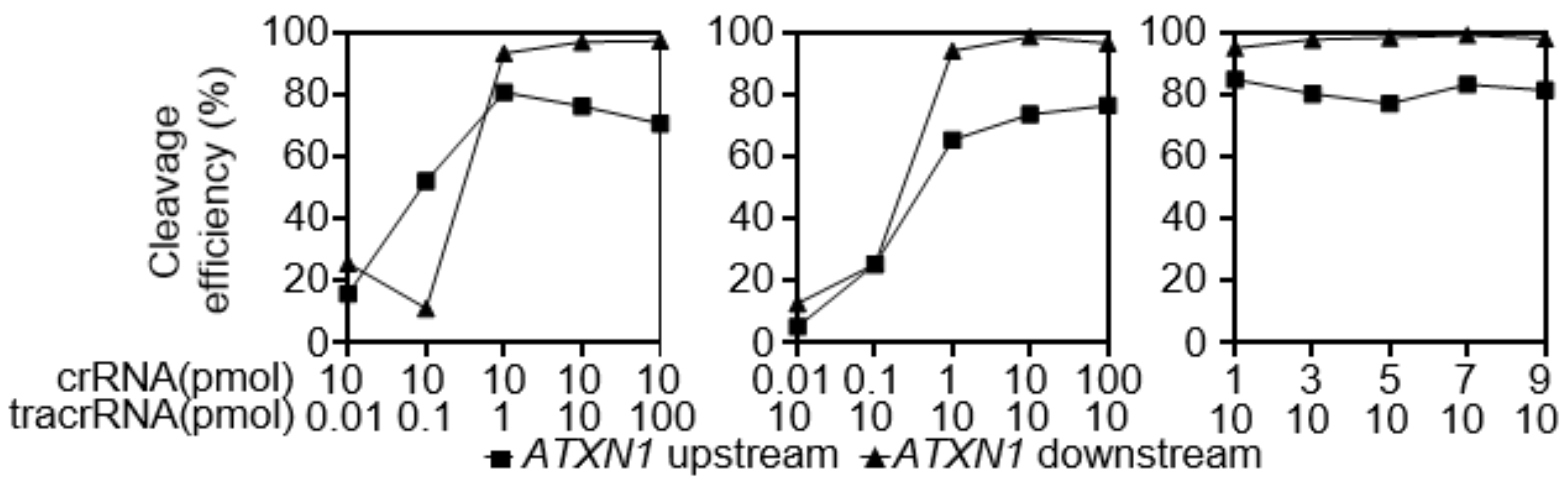

Figure S3. Impact of guide RNA selection on nCATS sequencing coverage and guide RNA optimization.

(A) Impact of guide RNA selection on nCATS sequencing coverage. Raw coverage depth obtained using selected and non-selected sgRNA pairs for representative STR loci. The dashed line indicates 30× coverage. (B) Schematic illustration of sgRNA and crRNA:tracrRNA guide RNA formats (left). Comparison of in vitro cleavage efficiencies between sgRNA and crRNA:tracrRNA for representative guide RNAs targeting NOTCH2NLC and NOP56 (right). Each dot represents an independent experiment. (C) Cost comparison between sgRNA and crRNA:tracrRNA for constructing the 56-locus STR panel. (D) In vitro cleavage efficiencies measured across different crRNA:tracrRNA ratios for representative guide RNAs targeting the upstream and downstream regions of ATXN1.

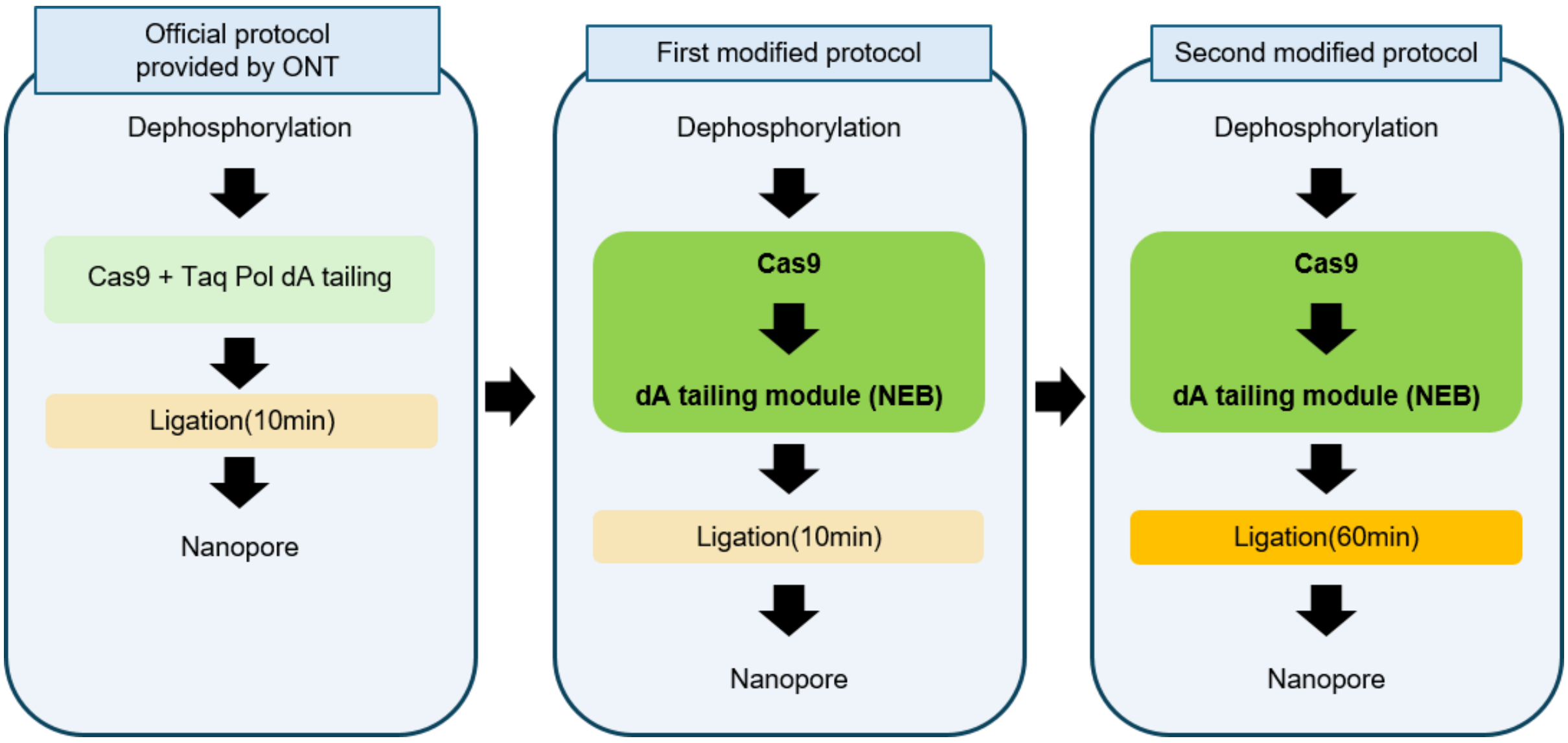

Figure S4. Schematic diagram with a comparison of the optimized nCATS protocol with the conventional protocol.

The optimized protocol employs sequential Cas9 cleavage at both ROI termini, followed by bead purification, rather than simultaneous Taq polymerase and Cas9 treatment. Further optimization includes dA-tailing using a Klenow fragment (exo-) (NEB) and an extended nanopore adapter ligation time to 60 minutes.

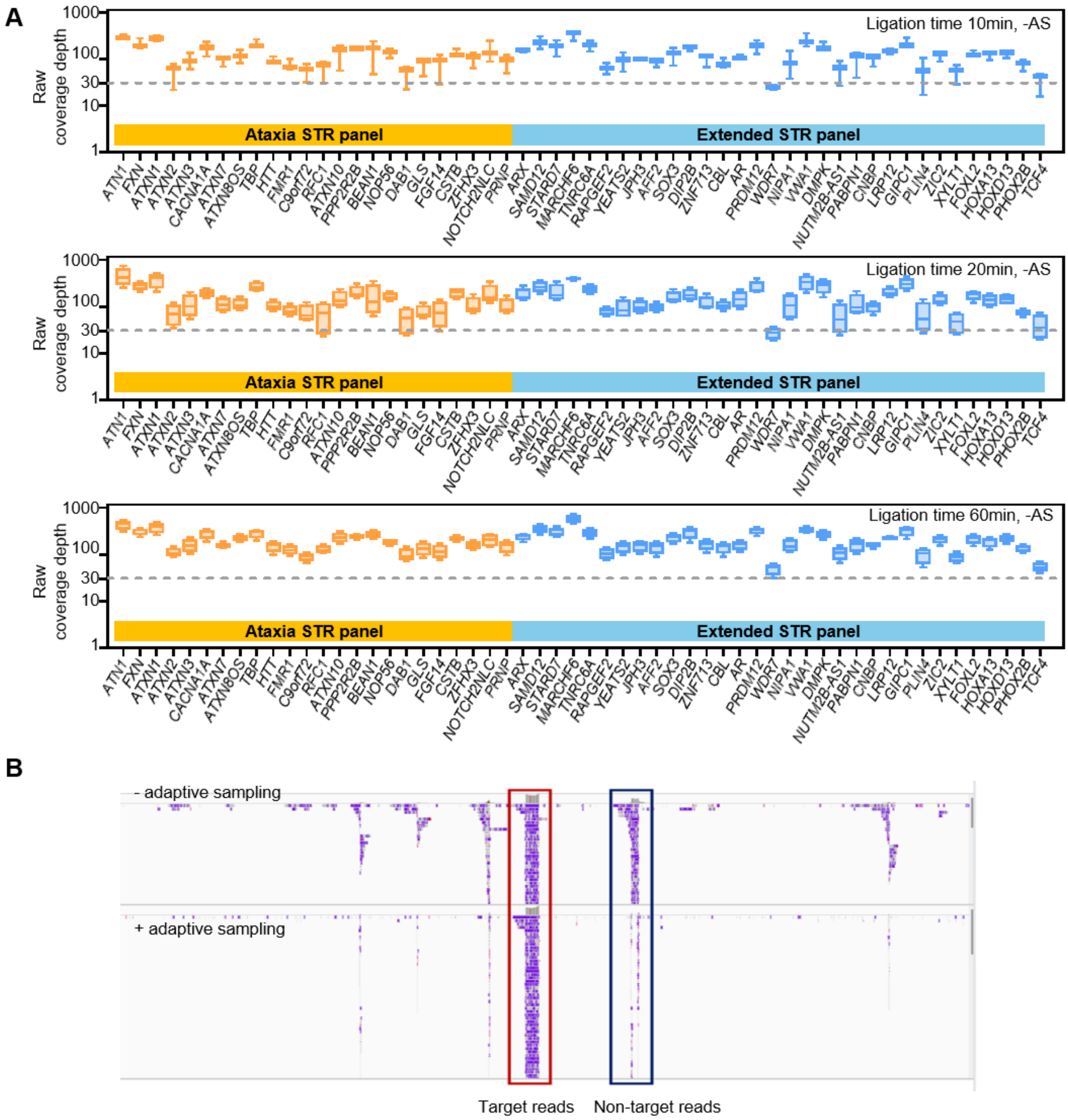

Figure S5. Optimization of ligation time and evaluation of adaptive sampling in the multiplexed nCATS workflow.

(A) Comparison of raw coverage depth across all 56 STR loci under 10-, 20-, and 60-min ligation conditions without adaptive sampling (−AS). Each box represents the distribution of raw coverage obtained from independent sequencing runs for each STR locus. The dashed line indicates 30× coverage. STR loci are grouped into the Ataxia STR panel (orange) and the Extended STR panel (blue). The 60-min ligation condition showed slightly more uniform coverage across the STR panel. (B) Representative IGV visualization comparing sequencing reads generated without adaptive sampling (−AS) and with adaptive sampling (+AS). Adaptive sampling reduced reads originating from non-target regions while increasing the proportion of reads mapped to target regions. Red and blue boxes indicate representative target and non-target regions, respectively.

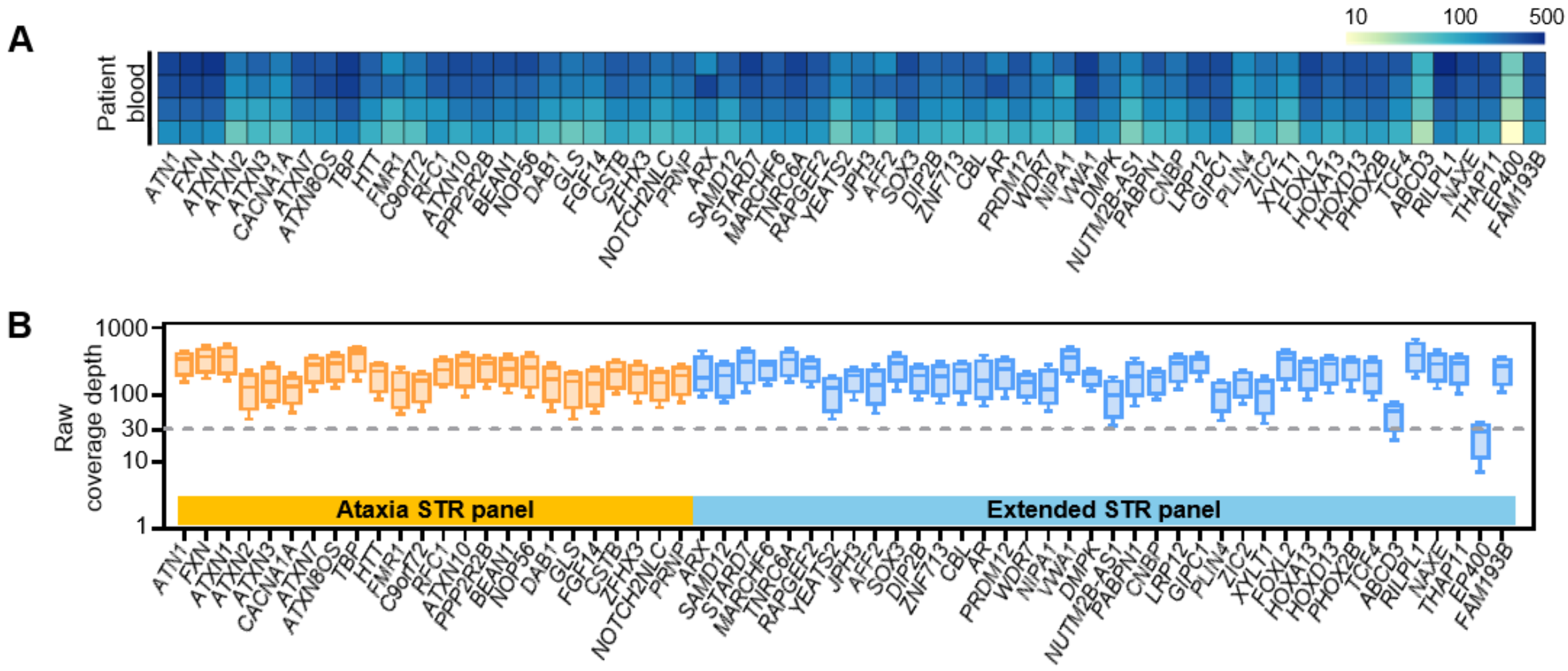

Figure S6. Coverage distribution across the 62-locus STR panel in patient-derived samples.

(A) Heatmap showing the average raw coverage depth across the 62 STR loci in patient-derived samples analyzed using the optimized nCATS workflow. Color intensity represents the average raw coverage depth.(B) Distribution of raw coverage depth across the 62 STR loci in patient-derived samples. Each box represents the distribution of raw coverage depth obtained from independent patient samples for each STR locus. The dashed line indicates 30× coverage. STR loci are grouped into the Ataxia STR panel (orange) and the Extended STR panel (blue).

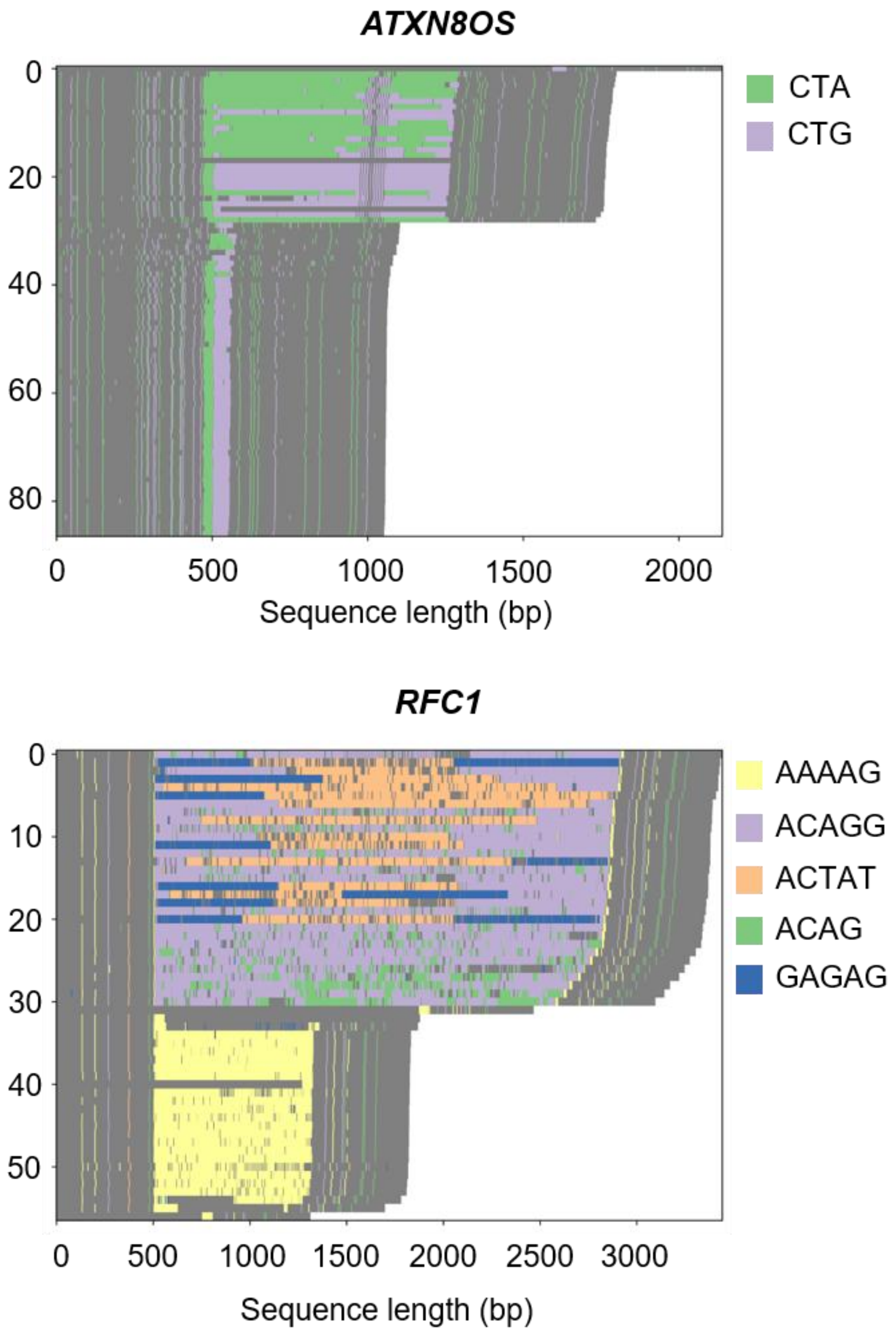

Figure S7. STRiker identifies a pathogenic length repeat expansion at *ATXN8OS* and *RFC1* that was not detected by HMMSTR.

Heatmaps show per-read motif composition in patient P15, with each row representing a single read and colors indicating repeat motif identity (legend, right). STRiker resolved pathogenic-length expansions at both loci that HMMSTR was unable to call.

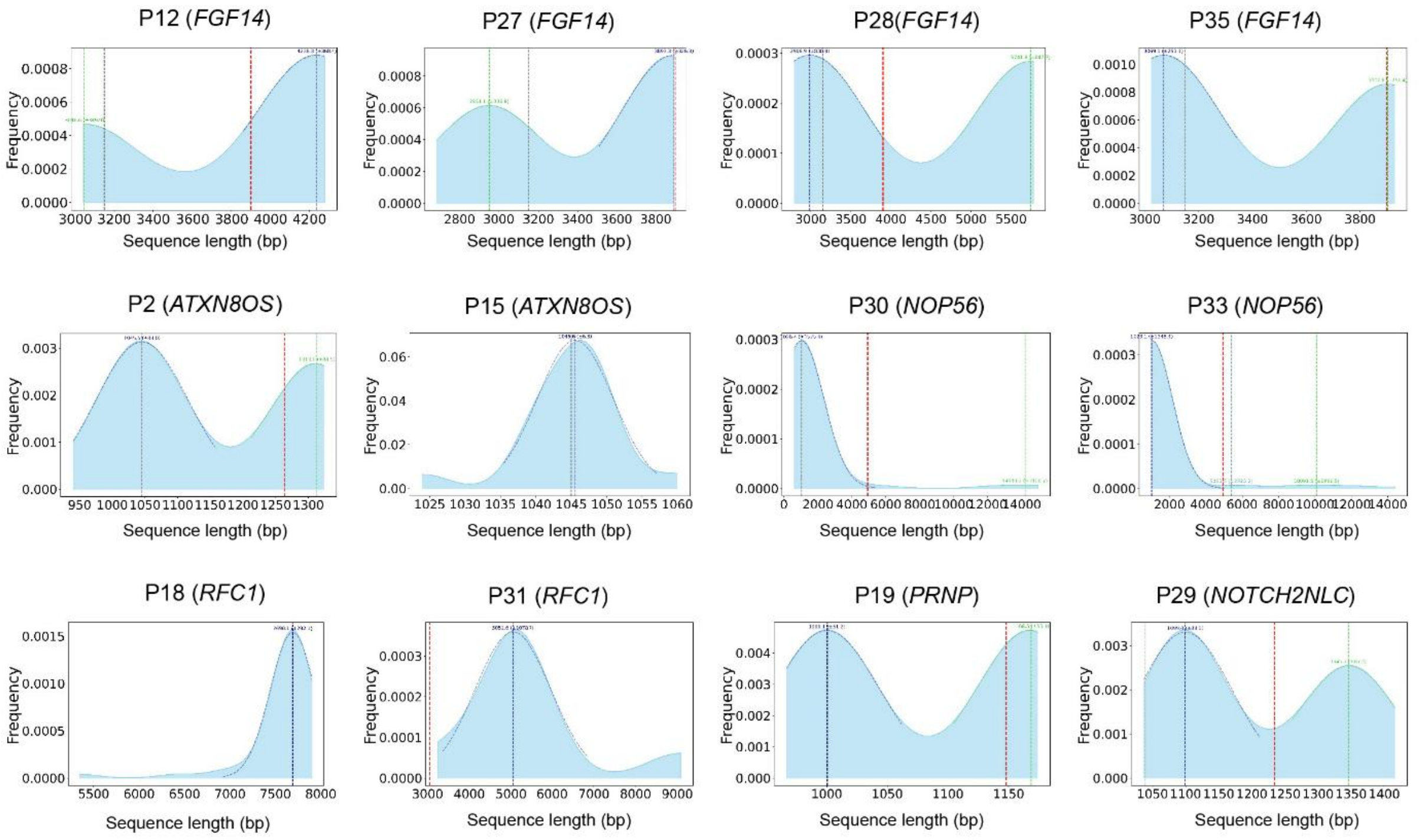

Figure S8. KDE read length distributions at expansion sites for newly diagnosed patients using the STRiker pipeline.

Read lengths span the repeat locus plus 500 bp flanking regions on each side. Gaussian-fitted peaks are shown in blue (primary) and green (secondary). The red line indicates the threshold for pathogenic expansion.

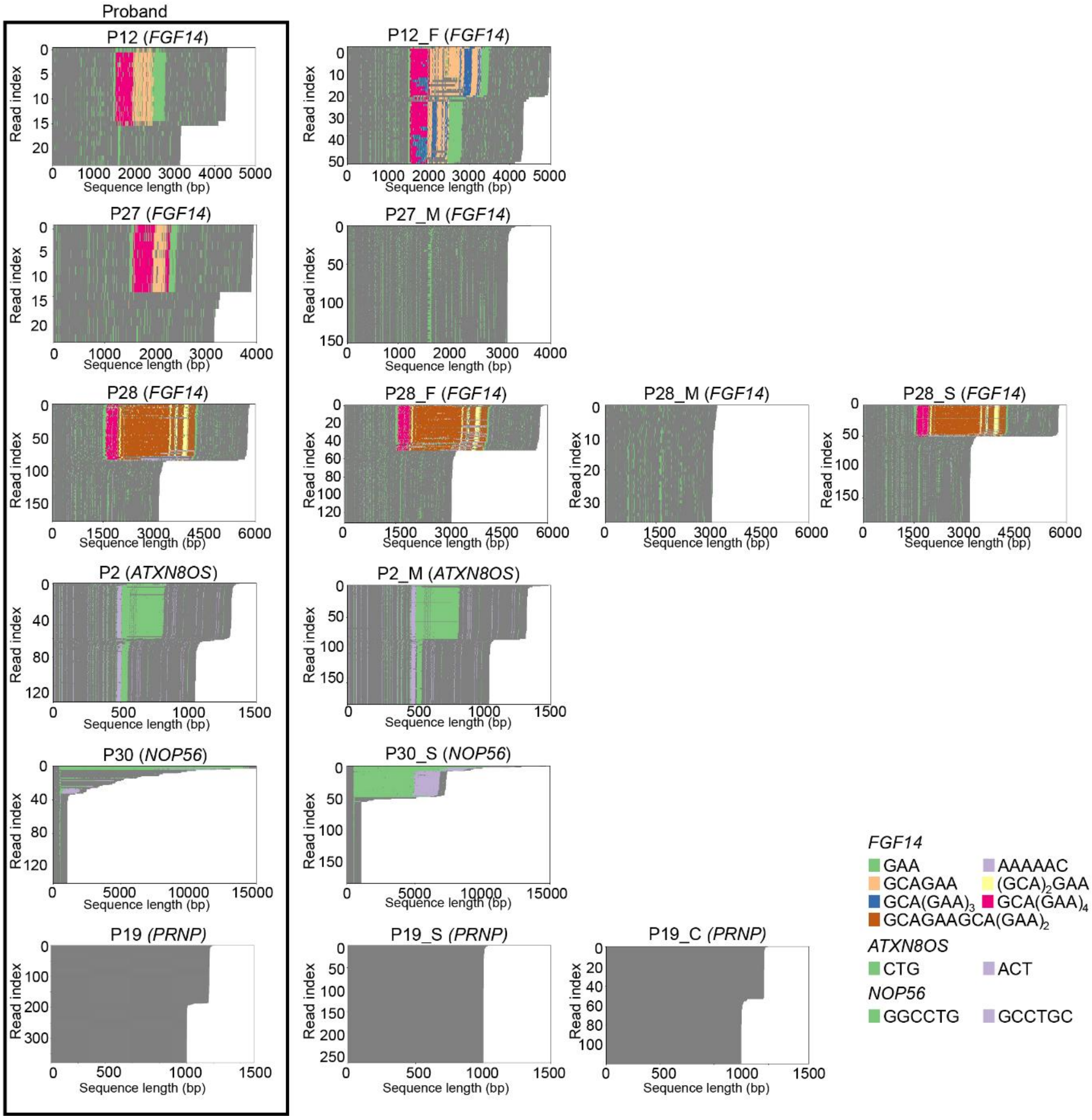

Figure S9. Repeat motif composition analysis in family members identified through cascade screening.

Heatmaps of the repeat motif composition for newly diagnosed patients and their relatives identified by cascade screening. Each panel represents long-read alignments at disease-associated loci, with distinct colors indicating different motif types for each gene. *PRNP* repeat expansions consist of an octapeptide motif of 24 bp (PHGGGWGQ), for which no distinct motif colors are applied.

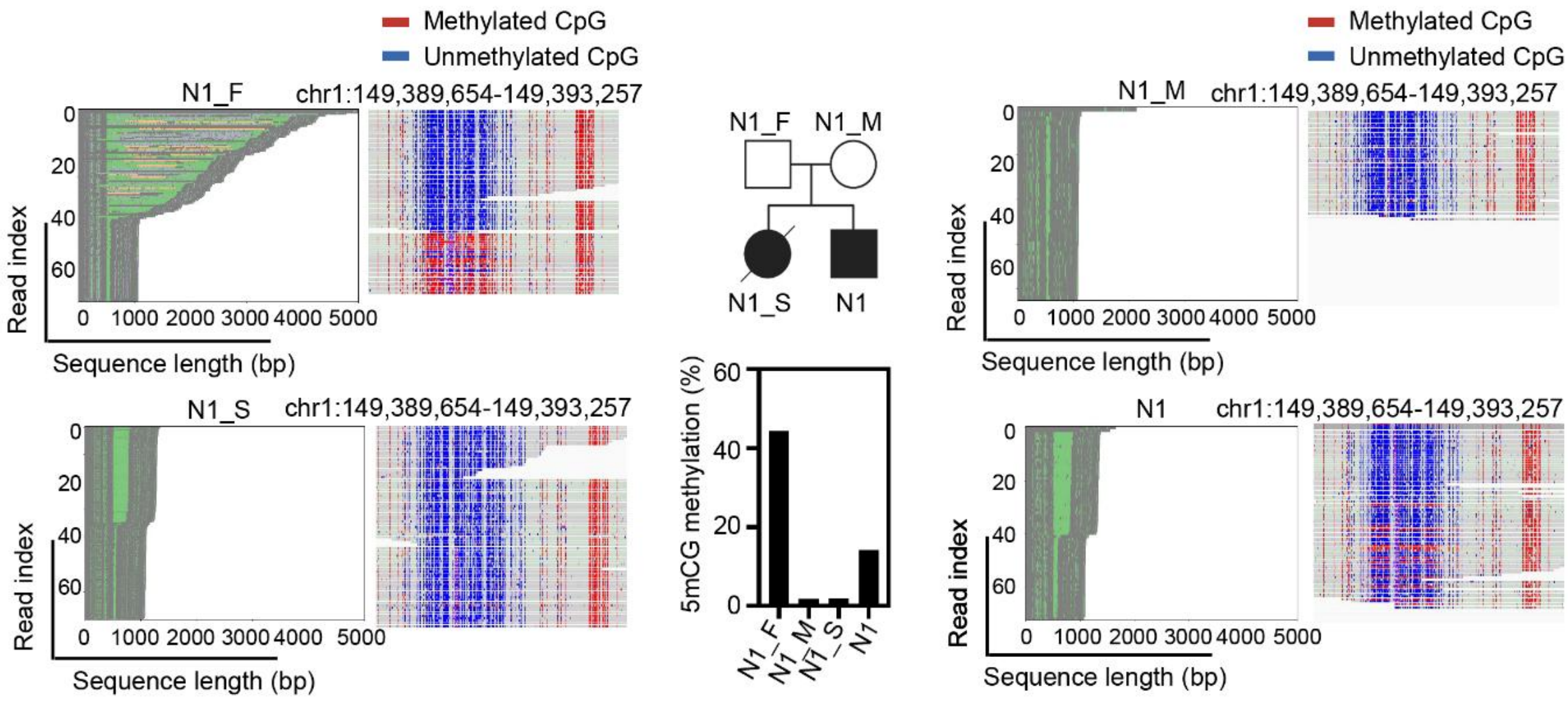

Figure S10. Case of contraction during paternal inheritance in a family with *NOTCH2NLC*-related neuronal intranuclear inclusion disease (NIID).

Long-read alignment and methylation profiles of each family member (N1_F, N1_M, N1, and N1_S). The father (N1_M) carried a long GGC repeat with hypermethylation and was asymptomatic, whereas the patient (N1) inherited a shorter allele with hypomethylation, which is likely associated with the development of neuronal intranuclear inclusion disease (NIID). The older sister (N1_S) of the patient also carried a hypomethylated expanded allele, was clinically affected, and died. Created with BioRender.

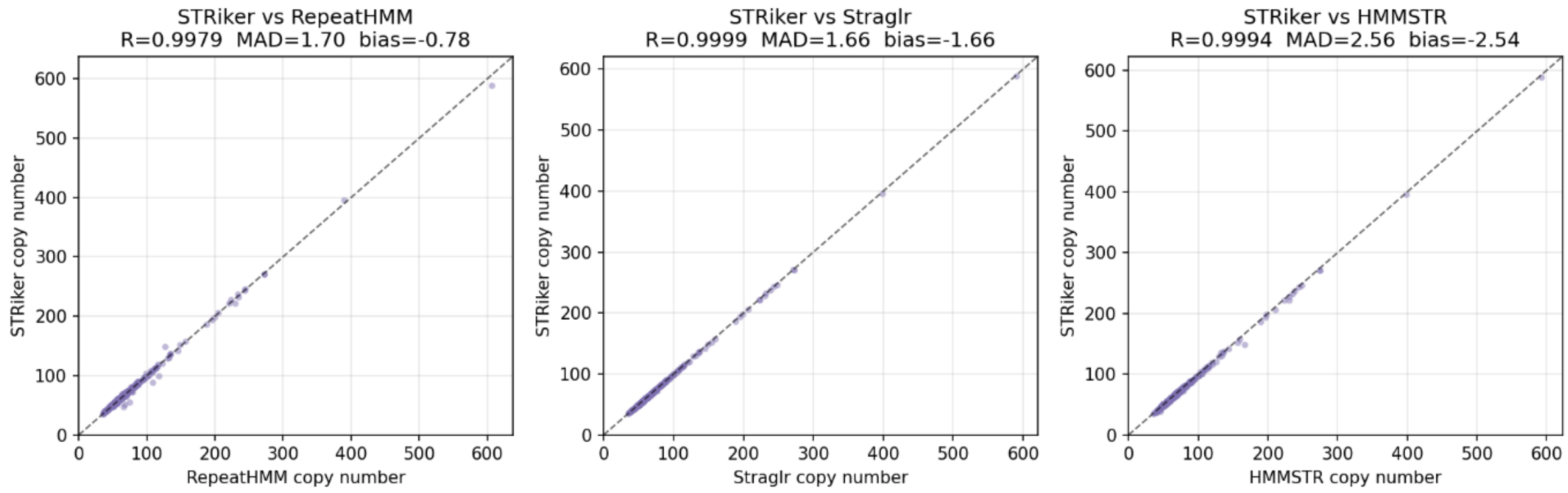

Figure S11. Concordance between STRiker and existing tools. (CHM13, 439 haploid loci).

Scatter plots comparing STR repeat-count calls from STRiker (y-axis) against RepeatHMM, Straglr, and HMMSTR (x-axis) across 439 benchmarked loci. The dashed line indicates the line of identity (y = x). Pearson correlation coefficient (R), mean absolute difference (MAD), and mean bias (STRiker minus comparator tool) are shown for each comparison.

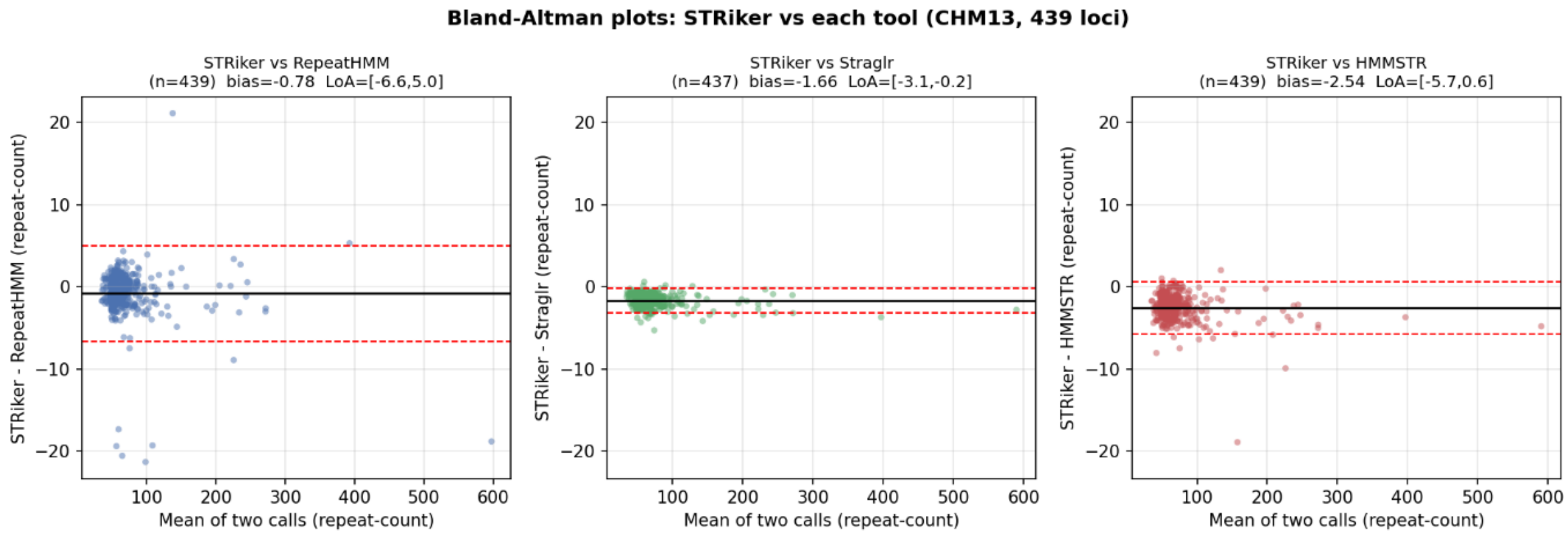

Figure S12. Bland-Altman analysis of agreement between STRiker and existing tools.

For each pairwise comparison, the difference between STRiker and the comparator tool's repeat-count call is plotted against the mean of the two calls. The solid black line indicates the mean bias; red dashed lines indicate the 95% limits of agreement (bias ± 1.96 SD). Narrower limits of agreement indicate closer concordance between tools.

Tool	Called regions	Genotyped	Excluded	Pearson R		MAD		Misclassified regions	Miscall rate
				H1	H2	H1	H2		
STRiker	13858	99.99%	0	0.8545	0.8545	1.323	1.323	3934	28.39%
HMMSTR	13850	99.93%	0	0.8129	0.8317	1.395	1.246	964	6.96%
Straglr	13094	94.47%	723	0.8369	0.8824	1.194	1.004	284	2.17%
RepeatHMM	7493	54.06%	0	0.6601	0.6897	3.166	2.754	1475	19.69%

Fig. S13. Genotyping accuracy of STRiker, HMMSTR, Straglr, and RepeatHMM on homozygous NA12878 loci.

Genotyping accuracy statistics for the same four tools against a haplotype-resolved homozygous NA12878 benchmark set (n = 4,731 loci). Miscall rate was counted over the intersection of loci genotyped by all four tools and expressed as a percentage of each tool's own total calls.

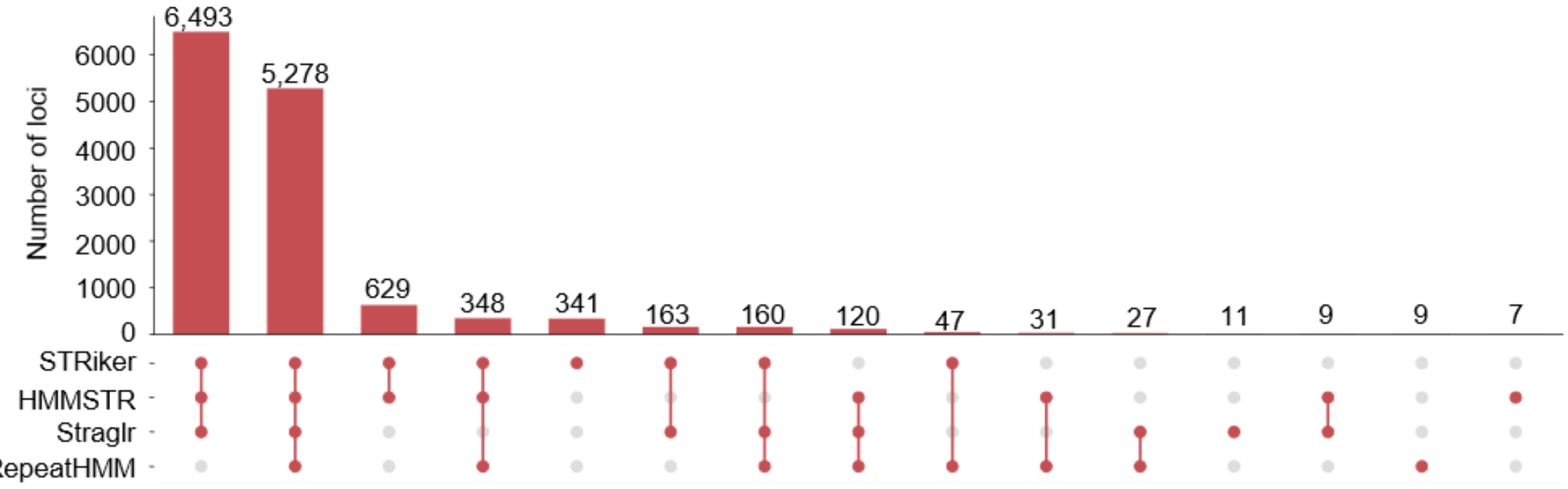

Fig. S14. Correctly-genotyped locus overlap among tools on homozygous NA12878 loci

Venn diagram of loci genotyped correctly by each tool against a homozygous NA12878 benchmark set (n = 13,860 chromosome 1 loci). Correct genotyping was defined as loci where both the H1 and H2 calls fell within a data-derived tolerance of ±4 copies of the truth. Bars indicate the number of loci in each intersection; filled circles below each bar denote which tool(s) contributed to that intersection, with connecting lines linking co-occurring tools.

Table S1. Patient demographics and clinical characteristics

**Characteristics**	**N (%)**
Total patients	37
Gender	
- Male	18 (48.6)
- Female	19 (51.4)
Age (yr), mean ± SD	43.7 ± 14.2
Family history	
- Yes	14 (37.8)
- No	23 (62.2)
Onset age (yr), mean ± SD	35.5 ± 17.5
Cerebellar atrophy	
- Yes	30 (88.2)
- No	4 (11.8)
Other brain abnormality	
- Yes	18 (52.9)
- No	16 (47.1)
Cognitive dysfunction	
- Yes	6 (16.2)
- No	31 (83.8)
Epilepsy	
- Yes	1 (2.7)
- No	36 (97.3)
Diagnosed	
- Yes	12 (32.4)
- No	25 (67.6)
Diagnosed gene	
- *FGF14*	4 (10.8)
- *ATXN8OS*	2 (5.4)
- *RFC1*	2 (5.4)
- *NOP56*	2 (5.4)
- *NOTCH2NLC*	1 (2.7)
- *PRNP*	1 (2.7)
- Undiagnosed	25 (67.6)

SD, standard deviation

Table S2. Clinical and genetic information of study participants

ID	Age at last follow-up (yr)	Gender	Family history	Affected family members	Onset age (yr)	Neurologic symptom	Gait at last follow-up	Cerebellar atrophy	Other brain abnormality	Cognitive dysfunction	Epilepsy	Previous genetic test	Diagnosed gene
Patient 1	42	Male	No	NA	40	Cognitive dysfunction, dysarthria, ataxia	Impossible	Yes	Diffuse brain atrophy	Yes	No	WES, gene panel	NA
Patient 2	44	Female	Yes	Mother	30	Dysarthria, ataxia	Possible (SARA gait 1)	Yes	No	No	No	Gene panel, SCA1,2,3,6,7,17, DRPLA, FRDA	*ATXN8OS*
Patient 2_M	NA	Female	NA	NA	NA	NA	NA	NA	NA	NA	NA	NA	*ATXN8OS*
Patient 3	59	Female	Yes	Sister	56	Ataxia, dysarthria, dysphagia	Possible (ataxic)	Yes	No	No	No	WES, SCA1,2,3,6,7,8,17	NA
Patient 4	39	Male	No	NA	29	Dysarthria, ataxia, but with symptom fluctuation	Possible (ataxic)	Yes	No	No	No	WES, gene panel, SCA1,2,3,6,7,17, DRPLA, FRDA	NA
Patient 5	43	Male	No	NA	37	Ataxia, dizziness, dysarthria, dysphagia, neurogenic bladder, expired	Impossible (since 42yr)	Yes	Severe atrophy of the pons and bilateral cerebellums with: 1) T2 HSI in the bilateral middle cerebellar peduncles, 2) a cruciform T2 HSI in the pons	No	No	WES, gene panel, SCA1,2,3,6,7,8,17, DRPLA, C9orf72	NA
Patient 6	51	Female	Yes	Father, sister	45	Ataxia	Possible (SARA gait 2)	NA	NA	No	No	WGS, WES, SCA1,2,3,6,7,17, DRPLA, FRDA	NA
Patient 7	55	Male	No	NA	53	Ataxia, spasticity	Possible	Yes	1) Atrophic change of the pons and cerebellum. 2) T2 HSI in the bilateral cerebral white matter, suggestive of small vessel disease	No	No	WES, SCA1,2,3,6,7,17, DRPLA, FRDA	NA

ID	Age at last follow-up (yr)	Gender	Family history	Affected family members	Onset age (yr)	Neurologic symptom	Gait at last follow-up	Cerebellar atrophy	Other brain abnormality	Cognitive dysfunction	Epilepsy	Previous genetic test	Diagnosed gene
Patient 8	41	Female	No	NA	29	Ataxia, dysarthria	Possible (SARA gait 2)	Yes	No	No	No	Gene panel, SCA1,2,3,6,7,8,17, DRPLA, FRDA	NA
Patient 9	69	Female	No	NA	68	Ataxia, gait disturbance	Possible	Yes	No	No	No	WES, SCA1,2,3,6,7,8,17, DRPLA	NA
Patient 10	31	Male	No	NA	1	Global developmental delay, intellectual disability, ataxia	Possible	Yes	No	Yes	No	WES, gene panel, SCA1,2,3,6,7,8,17, DRPLA	NA
Patient 11	48	Female	Yes	Father, brother, sister	40	Ataxia, gait disturbance	Possible	Yes	No	No	No	WES, gene panel, SCA1,2,3,6,7,8,17, DRPLA	NA
Patient 12	29	Male	Yes	Father, grandfather	25	Ataxia, gait disturbance, dysarthria	Possible	Yes	No	No	No	WES, gene panel, SCA1,2,3,6,7,8,17, C9orf72	*FGF14*
Patient 12_F	61	Male	NA	NA	35	Ataxia, gait disturbance	Possible (ataxic)	Yes	No	No	No	NA	*FGF14*
Patient 13	53	Female	No	NA	50	Ataxia, gait disturbance, dysarthria	Possible	Yes	Diffuse volume decrease at bilateral cerebellum, pons, and medulla	No	No	WES, gene panel, SCA1,2,3,6,7,17, DRPLA	NA
Patient 14	25	Female	Yes	Mother	21	Ataxia, gait disturbance, dysarthria	Impossible	Yes	No	No	No	Gene panel, SCA1,2,3,6,7,17, DRPLA	NA
Patient 15	28	Female	No	NA	21	Ataxia, dizziness	Possible (SARA gait 2)	Yes	No	No	No	WES, gene panel, SCA1,2,3,6,7,8,17, DRPLA	*ATXN8OS*
Patient 16	61	Male	No	NA	35	Ataxia, gait disturbance, dysarthria	Impossible	Yes	Diffuse brain atrophy	No	No	WGS, WES, SCA1,2,3,6,7,8,17	NA
Patient 17	35	Male	No	NA	13	Ataxia, dysarthria	Impossible	Yes	Diffuse brain atrophy	Yes	No	WES, gene panel SCA1,2,3,6,7,8,17, DRPLA	NA
Patient 18	64	Male	No	NA	57	Ataxia, gait disturbance, scanning speech	Possible (ataxic)	Yes	No	No	No	WES, gene panel SCA1,2,3,6,7,8,17, DRPLA	*RFC1*

ID	Age at last follow-up (yr)	Gender	Family history	Affected family members	Onset age (yr)	Neurologic symptom	Gait at last follow-up	Cerebellar atrophy	Other brain abnormality	Cognitive dysfunction	Epilepsy	Previous genetic test	Diagnosed gene
Patient 19	35	Female	Yes	Father, aunt	30	Ataxia, dysarthria, dizziness	Possible (ataxic)	Yes	Diffuse brain atrophy	Yes	No	WES, gene panel SCA1,2,3,6,7,8,17, DRPLA	*PRNP*
Patient 19_S	34	Male	NA	NA	NA	NA	NA	NA	NA	NA	NA	NA	NA
Patient 19_C	28	Male	NA	NA	NA	NA	NA	No	No	No	No	NA	*PRNP*
Patient 20	18	Male	No	NA	14	Ataxia, gait disturbance, dysarthria, dysphagia	Possible (ataxic)	Yes	No	No	No	WES, gene panel SCA1,2,3,6,7,8,17, DRPLA, FRDA	NA
Patient 21	30	Female	No	NA	28	Ataxia, gait disturbance, dysarthria, dysphagia, bradykinesia	Possible (ataxic)	Yes	Chiari malformation 1 with syringohydromyelia, mild midbrain atrophy	Yes	No	Gene panel SCA1,2,3,6,7,8,17, DRPLA	NA
Patient 23	64	Male	No	NA	60	Ataxia, dysarthria	Possible	Yes	Diffuse brain atrophy	No	No	Gene panel SCA1,2,3,6,7,8,17, DRPLA	NA
Patient 24	61	Female	No	NA	56	Dysarthria, ataxia, scanning speech, gait disturbance	Possible (ataxic)	Yes	Atrophic change in the bilateral cerebellar and brain stem	No	No	WES, gene panel SCA1,2,3,6,7,8,17, DRPLA	NA
Patient 25	44	Male	No	NA	39	Dysarthria, ataxia, gait disturbance	Possible (ataxic)	Yes	1) Underlying atrophic change in the brain parenchyma. 2) Mild degree T2 HSI lesions around bilateral lateral ventricles (posterior horns)	No	No	Gene panel SCA1,2,3,6,7,8,17, DRPLA	NA
Patient 26	39	Male	Yes	Mother	31	Dysarthria, ataxia	Possible	Yes	No	No	No	Gene panel SCA1,2,3,6,7,8,17, DRPLA	NA
Patient 27	33	Male	No	NA	1	Developmental delay, dysarthria, ataxia	Possible	Yes	No	No	No	WES, gene panel SCA1,2,3,6,7,8,17, DRPLA, FRDA, HD	*FGF14*
Patient 27_M	NA	Female	NA	NA	NA	NA	NA	NA	NA	NA	NA	NA	NA

ID	Age at last follow-up (yr)	Gender	Family history	Affected family members	Onset age (yr)	Neurologic symptom	Gait at last follow-up	Cerebellar atrophy	Other brain abnormality	Cognitive dysfunction	Epilepsy	Previous genetic test	Diagnosed gene
Patient 28	26	Female	Yes	Sister	7	Gait disturbance, ataxia, dystonia, spasticity	Possible (ataxic)	No	Bilateral T2 HIS at globus pallidus	No	No	LRS, WGS, WES, gene panel	*FGF14*
Patient 28_S	24	Female	NA	NA	3	Periodic paralysis, dystonia, ataxia, spasticity	Possible (ataxic)	No	Sublte T2 signal abnormalities in the both globus pallidus	No	No	LRS, WGS, WES, gene panel	*FGF14*
Patient 28_M	NA	Female	NA	NA	NA	NA	NA	NA	NA	NA	NA	NA	NA
Patient 28_F	NA	Male	NA	NA	NA	NA	NA	NA	NA	NA	NA	NA	*FGF14*
Patient 29	61	Female	No	NA	60	Transient cognitive impairment, ataxia	Possible	No	Bilateral confluent T2 HSI on white matter, positive zigzag edging sign	No	No	NOTCH2NLC	*NOTCH2NLC*
Patient 30	49	Male	Yes	Father, sister	45	Dysarthria, ataxia	Possible	NA	NA	No	No	Gene panel, SCA1,2,3,6,7,8,17, DRPLA	*NOP56*
Patient 30_S	52	Female	NA	NA	44	Abnormal sensation of lower limb, dysarthria, gait disturbance, ataxia	Possible (ataxic)	NA	NA	No	No	NA	*NOP56*
Patient 31	62	Female	No	NA	55	Gait disturbance, ataxia, peripheral neuropathy	Possible (ataxic)	No	Suspicious two tiny nodular isointense lesions, adjacent to the course of the right anterior inferior cerebellar artery	No	No	None	*RFC1*
Patient 32	17	Male	No	NA	12	Gait disturbance, ataxia	Possible (ataxic)	Yes	Pachy areas of increased T2 signal in the cerebral deep white matter	No	No	WGS, WES, gene panel, SCA1,2,3,6,7,8,17, DRPLA	NA

ID	Age at last follow-up (yr)	Gender	Family history	Affected family members	Onset age (yr)	Neurologic symptom	Gait at last follow-up	Cerebellar atrophy	Other brain abnormality	Cognitive dysfunction	Epilepsy	Previous genetic test	Diagnosed gene
Patient 33	60	Female	Yes	Aunt, uncle, cousin	55	Dizziness, gait disturbance, ataxia, mild scanning speech	Possible	Yes	No	No	No	WGS, WES, gene panel, SCA1,2,3,6,7,8,17, DRPLA, FRDA	*NOP56*
Patient 34	47	Male	Yes	Brother	33	Gait disturbance, dysarthria	Possible (ataxic)	Yes	No	No	No	WGS, WES, gene panel, SCA1,2,3,6,7,8,17, DRPLA	NA
Patient 35	26	Female	Yes	Mother, aunt, uncle, grandmother	25	Dysarthria, ataxia, mild scanning speech	Possible	NA	NA	No	No	Gene panel, SCA1,2,3,6,7,8,17, DRPLA	*FGF14*
Patient 36	46	Female	No	NA	43	Gait disturbance, dizziness, weakness, movement disorder	Possible	No	A few microbleeds at right frontal and occipital lob	No	No	Gene panel, SCA1,2,3,6,7,8,17, DRPLA	NA
Patient 37	51	Male	Yes	Father, grandfather	47	Gait disturbance, dysarthria, ataxia	Possible (ataxic)	Yes	1) Atrophic change of medulla and cervical spinal cord. 2) Confluent periventricular white matter hyperintensity. 3) A large supracerebellar arachnoid cyst.	No	No	None	NA

Patient IDs ending with M, F, S, and C denote relationships with the proband as follows: mother (M), father (F), sibling (S), and cousin (C).

NA, not available; WES, whole-exome sequencing; SARA, Scale for the Assessment and Rating of Ataxia; SCA, spinocerebellar ataxia; DRPLA, dentatorubral-pallidoluysian atrophy;

FRDA, Friedeich ataxia; HSI, high signal intensity; WGS, whole-genome sequencing; HD, Huntington disease; LRS, long-read sequencing

Table S3. HMMSTR genotyping results at STR loci identified by STRiker in patient samples

Patient	Gene	A1 (mode)	A2 (mode)	Over pathogenic expansion threshold
Patient 2	*ATXN8OS*	16	105	YES
Patient 12	*FGF14*	2	427	YES
Patient 15	*ATXN8OS*	17	18	NO
Patient 15	*RFC1*	2	163	NO
Patient 18	*RFC1*	2	1279	YES
Patient 19	*PRNP*	1	7	NO
Patient 27	*FGF14*	4	302	YES
Patient 28	*FGF14*	2	924	YES
Patient 29	*NOTCH2NLC*	34	116	YES
Patient 30	*NOP56*	0	7	NO
Patient 31	*RFC1*	833	840	YES
Patient 33	*NOP56*	9	1258	YES
Patient 35	*FGF14*	2	305	YES

Table S4. STR genotype program benchmarking using P28 sample with 22 loci

**Gene**	**Known Motif**	**RepeatHMM**	**Straglr**	**HMMSTR**	**STRiker**
ATN1	CAG	14/23	14.7/23.7	15/24	17.0 [1)]
FXN	GAA	11	16	16	15.9
ATXN1	CAG	25/26	2.2 *	25	24.7
ATXN2	CAG	22	3.7	22	21.0
ATXN3	CAG	19/27	19/27	19/27	20/24.7
CACNA1A	CAG	13/14	13.3	13	9.0
ATXN7	CAG	10	10.3	10	8.1
ATXN8OS	CAG/TAG	9/15	no motif detected	8/14	8.5
TBP	CAG	37	35.3	36	34.8
HTT	CAG	21	19.3	22	21.8
FMR1	CGG	30	53	53	51.8
C9orf72	GGGGCC	0	8 *	9	17.5 [2)]
RFC1	AAGGG/ACAGG	0	138.2 *	121/131	[3)]
ATXN10	ATTCT	14	14	14	13.4
PPP2R2B	CAG	9/10	5.2 *	10	8.8
BEAN1	TGGAA	0	8.8 *	5	8.1[4)]
NOP56	GGCCTG	9	8.3	7/9	no motif detected
DAB1	ATTTC	0	no motif detected	14	13.7
GLS	GCA	8/14	11.7	8/14	8.8
FGF14	GAA	695/1595	14.3/925.5	2/924	[5)]
CSTB	CCCCGCCCCGCG	0	4.3 *	2	no motif detected
NOTCH2NLC	CGG	12/33	15.3/31.7	10/13	29.0

1) KDE-based automatic motif fitting by STRiker did not converge to the expected distribution.

2) STRiker called the repeat motif as CCG instead of the known pathogenic motif.

3) Multiple motifs were detected beyond the known motif by STRiker, with an abnormally high sigma value.

4) STRiker called the repeat motif as ATCC and CCTT instead of the known pathogenic motif.

5) Multiple major motifs of varying lengths were detected, making it difficult to measure the length of a specific motif (see Fig. 4B).

***** The motif automatically identified by Straglr did not match the known pathogenic motif.
Motifs in a reverse-complement relationship were treated as identical and merged for downstream analysis.

Mod value was used when benchmarking HMMSTR

Table S5. Motif identification by STRiker and Straglr in patient sample P28

Gene	Known Motif	STRiker	Straglr
ATN1	CAG	CAG	AGC, AGAAGT, ATG, CCT, GAAGAGTAGAAGTAGAAGTAGAGAG, GTA, GTAGAAGTA
FXN	GAA	GAA	AAG, AAAAAGACAAAAAAATAAAG, AAAAATAAAA, TACAAAAAAA, AAAAT, AATAAAATAATAGAA, GCTGGGG, AGGG, CGC, AAA, AAAAAAAAG, AAAAAAAGA, GC, GTG
ATXN1	CAG	GCT	CAGTGG, TGC, GCTGG, GGGGTG, GGCTG, GCGCCGCCGC, GCT, TGGACG, GCTG, GGCT, TGGG, CTG, TCC, CCGTCGTC, CGT, TGCTGCTGCTTGCTGC
ATXN2	CAG	CTG	GCA, AGCCGGG, GCT, GGC, CCG, CGGCG, CGGCGAGGCG, CGG, CTG, TGCTGCTCCTGCTG, GGCGC, GGCG, GGGACAGCC, GCC, CGA, GCCGCT, GAAGGCGCGCCGCCGGGCACGCTAA, CGGCCGGGA, GC, GCG, GCCGGGA, GCT, GGC, GGCGCGGC, GGACAGCC, GACGGC, CTACAAAGGCAGCCGCGCCGGC, GCG
ATXN3	CAG	CTG	TGC
CACNA 1A	CAG	CTG, AGG	CTG
ATXN7	CAG	CAG, CGG	CAG, AAGG, AAGGAGG, AGG, AGGAGGAAGG, GGC, GTG, TGC
ATXN8 OS	CAG/TA G	CTG	no motif detected
TBP	CAG	CAG	CAG, AAGAAGGAAGGAGGAAGGAAGGAAGGAAGGAAG, ACTGCAC, GAAG, GAGT, GTA
HTT	CAG	CAG, GCC	CAG
FMR1	CGG	CGG, AAACC	GCG
C9orf7 2	GGGGC C	CCG	CCCCGC, CGCCG, CCCGCCCCGGG, CCGCCT, CGCAGG, CGCCGCAGG, GCCCCG, GCCCCGCCCCGACCACGCCCCCG, CCCCGGGCCCGCC, GCCTCC, GCCTCTCGCCACCGCCGGC, CCAC, CCACCG, CCGACC, CGCCCCGACCCCGA, CTTT, GCCCC, GCT, GGCCCGGCCCC, GCCCCGG, TTGT, CCGCCACCGCCG, CCGCCG, GCCCCGACCACGCCCCGCCCCCGGCCC, CG
RFC1	AAGGG/ ACAGG	AAAGA, ACAGG, AGAC, ATACT	GAAG, AAAGA, AAGAA, AGGAC, ATACT, GGACA, AAC, ACA, ACAGG, GCA, AAAG, TATAC, TATGA, CAG, GA, AAGA, GACAG, GAT, TA, AGGAG, GGAGA, TAT, AGAA, AGAGG, GAGAG, AATAG
ATXN1 0	ATTCT	ATTCT	TTCTA, TCTTC, TCTTCTCTTCTATTTCTTTC, TCTTCTCTTGTA, ATA, CTCAG, TATT, TCCA, TCTA, TTATTTA, TTTATTTATT, ATGTAA, TATGTAAA, TC
PPP2R 2B	CAG	CTG	CAGC, CGCACG, GCT, GGCGC, AGCTGGCAGGG, CACCCA, GCTGGAGGAG, AGCTGGCCAGGG, GCGCC, TGT, TGC, CAGCCGCGCA, CTG, GGCGCCAGCC
BEAN1	TGGAA	ATCC, CCTT	CCTT, CCATCCTGCCCTC, CCTCCCT, CT, CTTTCTCCTTTACCCT, TCCTT, TCTTCCCTC, TCTTTTCTTTTT, TTCTTTCTTTT, TTTC, TTTCTTT, TTTTTTTTTTCTTTCCTTTTTTATCCAATCTTATACTTTGCACCATCC CTGCCCTCCCATCCTCCCTCCTTTCCTTTAAATTTTCTTTCCTTCTCT TCCCTC
NOP56	GGCCT G	no motif detected	GGCCTG, GCCGGGCCTGGGCCTGCGCCTGC, GCCTGG, GCCTGCGCCTGGGCCTGG, GCGCCTGCGCCT, GCCTGC, GCTGG, CCTGCG, GGGCCC, GCGAC, GCGCCTG, GGCTGG, GCCGC, GTGAT, GGGGCGC
DAB1	ATTTC	AAAAT	no motif detected
GLS	GCA	GCA	CAG, AGCCTCGTGCGG
FGF14	GAA	GAA, AAAAAC,	GAA

		GAAGCA, GAAGCAGCA, GAAGCAGAAGCAGAA, GAAGAAGAAGCAGAA	
CSTB	CCCCGCCCCGCG	no motif detected	GGGGCGC, GGGGCGCGGCGGGGC, GCGCGGGGGCG, GCGCTCGG, GGGGC
NOTCH2NLC	CGG	CGG	GCG, AGG, CAG, CT

Table S6. STR genotyping outputs across analytical tools for the CHM13 reference sample (CHM13, 439 haploid loci)

Site	Chr	Start	End	Known Motif	Straglr	HMMSTR	STRiker
site_1	chr1	10361639	10361926	TTTC	67.2	66	66.09
site_2	chr1	10947433	10947651	TTTC	51.5	52	49.8
site_3	chr1	12542130	12542376	TCTT	59.0	60	56.97
site_4	chr1	18359870	18360136	GAAG	63.8	65	62.29
site_5	chr1	18745558	18746045	ATGG	120.8	120	118.45
site_6	chr1	23737046	23737419	AAGA	90.0	90	88.38
site_7	chr1	23904972	23905182	TTTC	51.5	52	50.24
site_8	chr1	23950970	23951172	TTTC	48.5	49	46.62
site_9	chr1	24000436	24000648	TTTC	51.2	53	49.57
site_10	chr1	24057196	24057470	TTTC	66.8	67	65.54
site_11	chr1	24104156	24104392	GATG	58.2	59	55.33
site_12	chr1	25002991	25003242	GGAA	61.8	63	60.11
site_13	chr1	29748700	29749008	TCTT	73.5	75	71.75
site_14	chr1	38208316	38208538	CTTT	54.5	55	52.51
site_15	chr1	42256092	42256308	TTTC	52.8	54	50.47
site_16	chr1	46677530	46677735	CTTT	50.0	52	48.74
site_17	chr1	54393751	54394026	AAACC	55.2	55	54.2
site_18	chr1	56435740	56435962	AGAA	53.2	54	52.28
site_19	chr1	63055837	63056049	AAGA	50.8	52	48.52
site_20	chr1	66770415	66770629	AAAG	52.2	52	50.62
site_21	chr1	69658670	69658965	AAAG	71.5	70	70.4
site_22	chr1	70451745	70451976	TATC	57.5	58	55.33
site_23	chr1	73747738	73747940	TTTC	49.2	49	47.59
site_24	chr1	94579275	94579493	TTTC	52.5	51	50.4
site_25	chr1	100375002	100375209	TTC	67.7	69	65.37
site_26	chr1	112020668	112020877	CTTT	50.8	53	49.04
site_27	chr1	118906444	118906851	AAAG	100.0	101	98.49
site_28	chr1	152100576	152100779	AGAA	49.8	50	48.59
site_29	chr1	161394056	161394309	TTTC	61.5	62	59.78
site_30	chr1	163853858	163854078	AGAA	53.2	53	51.5
site_31	chr1	173553541	173553776	TTCT	57.8	57	56.35
site_32	chr1	181953640	181953864	AAAG	54.8	55	52.99
site_33	chr1	191254051	191254289	TTCT	56.5	58	54.52
site_34	chr1	191903675	191903879	AAAG	47.8	47	46.2
site_35	chr1	204032990	204033246	TCTT	62.8	62	60.92
site_36	chr1	206008073	206008320	TCTT	60.2	59	57.74
site_37	chr1	215290741	215290961	TTCCT	43.2	46	42.2
site_38	chr1	221066672	221066890	GAAA	53.5	54	52.17
site_39	chr1	229820285	229820487	TCTT	49.0	51	47.37
site_40	chr1	233526375	233526597	AATAA	44.6	45	44.14
site_41	chr1	244852513	244852725	TCCA	52.0	53	50.31

site_42	chr10	5325940	5326204	AAAG	65.2	67	62.66
site_43	chr10	11673649	11673939	AGAA	71.5	73	70.43
site_44	chr10	14140989	14141203	CTTT	51.2	52	49.57
site_45	chr10	21246357	21246578	TTC	71.3	71	68.6
site_46	chr10	25899299	25899655	AGGGA	68.4	70	67.25
site_47	chr10	27378237	27378490	TTTC	61.8	61	59.9
site_48	chr10	29034994	29035233	TTTTA	47.4	49	46.21
site_49	chr10	31606392	31606667	AAAG	67.0	67	65.05
site_50	chr10	38753330	38753546	AAAG	53.2	54	51.12
site_51	chr10	63747654	63748009	AGAA	86.6	88	85.05
site_52	chr10	69112677	69112901	AAGA	55.0	56	53.4
site_53	chr10	79391975	79392287	TTTC	75.5	76	74.09
site_54	chr10	90437826	90438033	AAAG	49.5	51	47.98
site_55	chr10	90873973	90874204	TTTC	56.0	57	53.85
site_56	chr10	100390188	100390439	AAAG	61.2	62	59.95
site_57	chr10	118839423	118839640	CTTT	52.5	55	50.48
site_58	chr10	129901811	129902042	AAAG	54.0	55	51.57
site_59	chr11	741003	741248	AGGGG	47.2	49	46.08
site_60	chr11	3305402	3305766	TCCA	90.0	91	87.7
site_61	chr11	3960910	3961147	TTCT	57.5	59	55.82
site_62	chr11	8257523	8257966	TCTC	106.5	108	105.03
site_63	chr11	16230177	16230442	AAAG	64.0	65	62.3
site_64	chr11	27658050	27658345	TCTT	71.5	72	69.69
site_65	chr11	37897317	37897537	TTTC	53.5	55	52.2
site_66	chr11	44621172	44621423	TTTC	60.8	62	59.67
site_67	chr11	46325481	46325741	AAAG	64.1	65	62.87
site_68	chr11	57353445	57353818	AAG	123.0	126	119.74
site_69	chr11	60398203	60398647	TATGC	87.8	89	86.68
site_70	chr11	75056935	75057152	TTTC	50.2	51	48.81
site_71	chr11	80017924	80018139	TCCT	53.2	54	51.02
site_72	chr11	82229329	82229713	GAAA	94.0	95	92
site_73	chr11	89676752	89676987	AAGA	57.0	60	55.25
site_74	chr11	105165943	105166195	AGAA	62.5	65	61.25
site_75	chr11	110940816	110941039	TAGCA	43.8	45	42.97
site_76	chr11	118113194	118113451	AGAA	62.8	61	60.57
site_77	chr11	122014910	122015470	AAAG	137.8	137	136.69
site_78	chr11	122396674	122396877	GAAA	45.5	46	42.71
site_79	chr11	130632369	130632903	TAGA	132.2	134	129.47
site_80	chr12	214123	214347	TGGA	55.2	57	53.96
site_81	chr12	5910220	5910514	GGGAG	56.1	58	55.28
site_82	chr12	39484521	39484817	AGAA	72.6	73	70.46
site_83	chr12	40498971	40499217	TTTC	59.6	60	57.89
site_84	chr12	53777856	53778324	TTTC	114.2	115	112.03
site_85	chr12	68256752	68256958	AAAG	48.8	49	47.25
site_86	chr12	69870717	69870989	TTTC	66.2	70	65.72

site_87	chr12	70073043	70073285	AAGA	57.0	57	56.37
site_88	chr12	71322526	71322748	AAG	72.8	73	70.78
site_89	chr12	74637949	74638173	TTTC	54.8	56	52.81
site_90	chr12	81023783	81024040	CTTT	63.2	64	61.06
site_91	chr12	88727444	88727659	AGAA	51.8	53	50.03
site_92	chr12	93595794	93596074	TTTC	66.2	67	63.87
site_93	chr12	97923852	97924110	AGAA	62.5	62	60.06
site_94	chr12	99342974	99343181	AGAT	51.4	52	50
site_95	chr12	101440286	101440510	AAAG	54.1	53	51.9
site_96	chr12	103978325	103978618	AAAG	70.8	70	69.94
site_97	chr12	114852503	114852706	GAGGG	38.0	40	37.17
site_98	chr12	115352856	115353814	GGAA	238.8	239	236.71
site_99	chr12	126657471	126657774	AAGA	74.2	75	72.93
site_100	chr12	129463817	129464067	ATGG	61.5	63	60.06
site_101	chr13	24675376	24675664	TTTC	70.8	71	67.57
site_102	chr13	27695863	27696087	GAAA	54.5	56	53.23
site_103	chr13	33804243	33804467	AGAA	53.8	55	52.83
site_104	chr13	54379360	54379598	TTCT	57.0	60	56.24
site_105	chr13	54916943	54917164	TTC	72.3	74	68.92
site_106	chr13	77885035	77885283	AAAG	58.2	59	56.77
site_107	chr13	101377549	101377792	GAA	76.8	79	71.52
site_108	chr13	108575965	108576209	AAAG	59.5	60	58.45
site_109	chr14	1794091	1794292	TTTC	48.2	48	47.07
site_110	chr14	14207500	14207777	AGGG	68.5	69	66.68
site_111	chr14	38196066	38196275	AGA	68.5	69	67.81
site_112	chr14	69181130	69181349	TTTC	53.2	54	52.13
site_113	chr14	74131782	74132009	TTC	72.7	74	69.85
site_114	chr14	74135730	74135969	AAGA	57.2	60	55.4
site_115	chr14	77312697	77312931	TTTC	58.2	59	56.94
site_116	chr14	80686286	80686598	GAAG	77.0	77	75.31
site_117	chr14	84721133	84721584	CCTCC	87.0	87	84.55
site_118	chr14	86480641	86480844	AGAA	49.0	50	47.91
site_119	chr14	92186096	92186308	CTTT	51.8	54	50.67
site_120	chr14	93460493	93460746	ATGG	62.5	63	59.89
site_121	chr14	93868422	93868709	GAAA	70.2	70	69.46
site_122	chr15	46942	47168	AAG	74.3	74	72.66
site_123	chr15	2202700	2202908	TTTC	49.6	50	48.32
site_124	chr15	36600364	36600596	CTTT	56.5	57	54.81
site_125	chr15	37798370	37798586	TCCA	53.2	54	50.74
site_126	chr15	50747481	50747687	CTTT	50.5	51	48.41
site_127	chr15	51139248	51139484	TTCT	57.8	60	56.32
site_128	chr15	67123077	67123364	TTTC	70.5	71	67.75
site_129	chr15	67144967	67145199	AAG	75.7	77	72.76
site_130	chr15	67500498	67500715	CTTTC	42.4	45	40.9
site_131	chr15	67815487	67815890	AAAG	99.0	101	96.67

site_132	chr15	71705863	71706072	TCC	68.7	69	67.24
site_133	chr15	72860917	72861161	TTCT	56.9	57	55.23
site_134	chr15	75396158	75396397	TTTC	58.5	58	56.95
site_135	chr15	78931413	78931690	TTTAT	55.2	57	53.62
site_136	chr15	86021942	86022384	GAAA	107.2	108	105.13
site_137	chr15	88841779	88841979	TTCC	49.2	50	46.57
site_138	chr15	99677410	99677648	TTTC	57.8	59	55.94
site_139	chr16	5484409	5484727	TCTT	77.5	77	75.57
site_140	chr16	15290437	15290651	GAAA	26.1	54	50.27
site_141	chr16	19971908	19972184	AGGAG	53.6	54	52.32
site_142	chr16	23096338	23097237	TTCC	223.2	224	221.11
site_143	chr16	23258315	23258571	TGGA	63.5	64	59.9
site_144	chr16	25897955	25898159	CTTT	49.8	51	48.57
site_145	chr16	26834360	26834594	TTTC	57.8	59	54.97
site_146	chr16	28227773	28228035	AGAA	63.5	65	60.53
site_147	chr16	31721829	31722140	TTTC	75.8	76	74.03
site_148	chr16	32800050	32800277	TCTT	55.2	56	52.59
site_149	chr16	34678921	34679149	GAAA	56.0	57	53.64
site_150	chr16	53739268	53739470	TTC	65.7	67	62.59
site_151	chr16	55750686	55750923	AGAA	57.2	58	52.89
site_152	chr16	58755910	58756124	AAAG	52.0	53	49.9
site_153	chr16	58790203	58790412	AAAG	51.0	50	50.05
site_154	chr16	60145080	60145324	TTTC	59.2	59	57.04
site_155	chr16	67213725	67214173	TTTC	108.8	112	107.03
site_156	chr16	67925255	67925459	ATCT	50.8	51	48.8
site_157	chr17	3288093	3288294	AAAG	48.6	50	47.86
site_158	chr17	5777814	5778028	CCCTC	39.6	42	39.39
site_159	chr17	37649480	37649697	TTCT	52.2	52	49.25
site_160	chr17	41900081	41900362	TTCT	34.5	69	67.84
site_161	chr17	57755613	57755822	GAA	67.5	69	65.44
site_162	chr17	62991808	62992066	AAGA	63.0	64	62.12
site_163	chr17	64861841	64862049	TCTT	50.2	51	47.7
site_164	chr17	64885120	64885532	GAAA	101.1	103	98.71
site_165	chr17	69363025	69363259	TTCT	57.2	58	56.1
site_166	chr17	69458218	69458447	TTTC	55.2	54	53.41
site_167	chr17	80378675	80380278	CCTC	399.0	399	395.3
site_168	chr17	81566745	81567117	TTTCC	73.6	73	72.9
site_169	chr18	743137	743422	TCTT	70.0	71	67.64
site_170	chr18	821216	821905	GATGGT	114.3	115	112.88
site_171	chr18	9804689	9804903	AGAT	52.8	55	51.57
site_172	chr18	22263567	22263790	TTTC	54.5	54	53.4
site_173	chr18	22638524	22638959	CCCCAG	73.0	73	71.09
site_174	chr18	23283085	23283451	GAAA	90.0	94	90.19
site_175	chr18	27247114	27247384	AAG	89.7	90	87.28
site_176	chr18	39929103	39929362	AGAA	62.5	61	60.95

site_177	chr18	63289470	63289699	TTCT	56.0	57	55.04
site_178	chr18	71427383	71427590	TCCA	51.2	52	48.94
site_179	chr18	72575919	72576135	TTTC	52.4	53	50.82
site_180	chr19	8531218	8531486	GGTGGT	44.2	45	42.11
site_181	chr19	8715704	8716094	ATCC	96.5	98	94.7
site_182	chr19	15775930	15776243	TTTC	76.5	76	75.84
site_183	chr19	16197394	16197738	AGAA	82.5	85	82.32
site_184	chr19	19881629	19881963	TTCCT	64.6	65	62.46
site_185	chr19	21673853	21674068	TTTC	52.0	52	51.21
site_186	chr19	30857881	30858095	CCCTT	41.0	43	38.95
site_187	chr19	31626409	31626650	CTTT	56.8	56	55.2
site_188	chr19	33522821	33523111	CCTT	71.5	73	69.77
site_189	chr19	36139093	36139392	TCT	97.3	97	95.74
site_190	chr19	44609110	44609317	AGAA	50.5	52	49.64
site_191	chr19	59802904	59803104	TTTC	48.5	49	46.43
site_192	chr19	59836487	59836769	TTTC	68.0	70	66.77
site_193	chr19	61523244	61523516	CTTT	63.8	64	62.19
site_194	chr2	5689117	5689414	AGAA	72.8	72	71
site_195	chr2	10427771	10429247	CACCAC	243.7	245	242.82
site_196	chr2	17509653	17509951	AAAG	72.5	71	71.11
site_197	chr2	21095222	21095422	TCTCA	39.0	40	37.55
site_198	chr2	26973690	26973895	AAAG	48.5	49	47.24
site_199	chr2	29845895	29846134	GGAGG	46.0	48	43.89
site_200	chr2	57745050	57745273	AAAG	54.8	57	52.58
site_201	chr2	59859106	59859368	AGAT	64.5	65	63.26
site_202	chr2	67916116	67917322	GGAAA	232.2	236	231.88
site_203	chr2	74569617	74569827	GGA	69.3	70	67.7
site_204	chr2	98505959	98506242	CCTTT	55.6	57	54.86
site_205	chr2	104106492	104106696	GAAA	50.0	50	49.33
site_206	chr2	105408346	105408546	AGAA	48.5	50	46.72
site_207	chr2	107253198	107253432	TTC	76.0	76	72.94
site_208	chr2	109659823	109660841	AATGG	199.3	198	197.79
site_209	chr2	110285064	110285274	CTTTT	40.6	42	39.58
site_210	chr2	113226885	113227183	TTTC	72.2	74	69.86
site_211	chr2	114822018	114822437	TTTC	100.2	106	99.61
site_212	chr2	115306391	115306617	CTAT	56.2	56	54.46
site_213	chr2	125144157	125144646	AAG	160.0	160	157.01
site_214	chr2	132842991	132843194	TTCC	49.8	50	48.16
site_215	chr2	136743544	136743781	TTTC	56.0	56	54.79
site_216	chr2	146065603	146065966	AGAA	87.1	88	85.72
site_217	chr2	146504629	146504829	AAAG	49.0	50	48.08
site_218	chr2	151585184	151585596	GAAA	97.5	99	96.21
site_219	chr2	153798105	153798333	TTTC	55.2	55	53.99
site_220	chr2	163209556	163209909	AAAAG	67.1	70	66
site_221	chr2	176861612	176861872	CTTT	63.2	64	61.26

site_222	chr2	204544640	204544915	TTCC	67.8	66	66.74
site_223	chr2	206470697	206470915	TAAAA	43.2	45	40.86
site_224	chr2	207528441	207528651	AGA	68.7	69	65.43
site_225	chr2	221031196	221031611	TTC	136.0	133	135.02
site_226	chr2	221822656	221822874	AAAG	51.5	55	50.35
site_227	chr2	222051790	222052082	AGAAGA	48.0	48	47.25
site_228	chr2	227910599	227910883	TTTC	69.5	69	67.93
site_229	chr2	232336555	232336818	TTTC	63.8	65	62.68
site_230	chr2	232871781	232872060	AAG	92.2	92	89.86
site_231	chr2	237006165	237006473	TCTT	73.8	74	71.91
site_232	chr20	379529	379741	TTC	69.7	71	67.19
site_233	chr20	1831355	1831582	ATGG	55.9	57	53.1
site_234	chr20	4153189	4153430	TTC	79.3	81	78.41
site_235	chr20	17398477	17398792	GAAA	76.2	77	73.15
site_236	chr20	23620472	23620693	TCTT	52.0	54	50.48
site_237	chr20	33598270	33598487	AAAG	53.2	55	52.26
site_238	chr20	33614595	33614862	TCTT	64.4	67	63.02
site_239	chr20	39919652	39920750	TCCA	271.4	275	270.39
site_240	chr20	40793392	40793655	CTTT	64.8	65	63.74
site_241	chr20	43439151	43439466	AGAA	77.4	78	75.67
site_242	chr20	62129627	62129894	CTTT	62.8	66	61.04
site_243	chr20	62488464	62488678	TCTT	51.6	52	50.64
site_244	chr20	62881783	62882025	GAAA	59.2	61	57.07
site_245	chr20	64674773	64675588	CACCCC	136.5	136	134.32
site_246	chr21	12131601	12131819	GAAA	52.5	54	50.82
site_247	chr21	12831483	12831809	AAAG	79.4	78	78.19
site_248	chr21	13663391	13663604	AAAG	52.2	51	51.08
site_249	chr21	15755013	15755295	AAAG	68.8	69	66.51
site_250	chr21	21907411	21907622	TTCT	52.0	54	51.19
site_251	chr21	27194614	27194957	AAAG	84.2	85	82.97
site_252	chr21	27926119	27926483	TTTC	88.8	89	87.6
site_253	chr21	36585444	36585744	TATC	74.5	74	72.8
site_254	chr21	37968298	37968562	TTCCT	51.2	52	50.44
site_255	chr21	39956558	39956768	ATGG	51.8	53	49.3
site_256	chr22	20874950	20875182	TGGA	57.5	58	56.17
site_257	chr22	27301707	27301957	GAAA	61.5	61	59.57
site_258	chr22	27939065	27939445	TTTGG	75.6	76	74.76
site_259	chr22	33553691	33553948	TTC	83.7	84	81.98
site_260	chr22	37417959	37418746	ATGG	195.1	197	193.11
site_261	chr22	42051178	42051403	CCAT	55.5	56	53.72
site_262	chr22	44395647	44395901	CTT	83.3	81	80.52
site_263	chr22	44463228	44463987	CCAT	189.0	190	185.59
site_264	chr22	49724479	49724732	AGAA	62.0	60	60.15
site_265	chr22	49728909	49729132	ATGG	55.0	56	53.8
site_266	chr3	2281478	2281699	AAGAAG	36.2	37	35.29

site_267	chr3	28799493	28799830	TCTT	82.0	84	79.64
site_268	chr3	36919818	36920045	AGAA	55.0	56	53.4
site_269	chr3	63451702	63451968	AAGA	64.2	65	62.56
site_270	chr3	70667617	70667863	GAAA	59.5	61	57.98
site_271	chr3	76887532	76887866	TTTC	81.8	80	80
site_272	chr3	85015404	85015713	AAAG	75.8	76	73.71
site_273	chr3	86407515	86407859	TTTAT	69.6	70	68.53
site_274	chr3	88522059	88522303	TTTC	59.2	60	58.58
site_275	chr3	106338361	106338585	AAAGA	44.0	44	42
site_276	chr3	111760610	111760820	GAAA	50.2	51	47.71
site_277	chr3	114501109	114501320	AAG	66.3	70	63.87
site_278	chr3	124274816	124275024	GAAA	51.0	52	49.67
site_279	chr3	124474740	124474940	AGAA	48.8	50	47.15
site_280	chr3	136808921	136809187	GAAA	63.8	65	61.97
site_281	chr3	137144037	137144284	TTCT	57.2	59	56.06
site_282	chr3	140497718	140497938	GAAA	54.0	54	52.6
site_283	chr3	144226492	144226780	AAG	95.0	96	91.96
site_284	chr3	151131974	151132177	AAAG	49.5	51	47.37
site_285	chr3	162794613	162794923	GAAA	76.0	77	74.61
site_286	chr3	162846507	162846791	TTTAT	56.4	56	55.9
site_287	chr3	169211146	169211349	AGAA	49.2	50	47.76
site_288	chr3	172162249	172162524	AAAG	66.8	68	64.49
site_289	chr3	185067893	185068094	AGAA	48.0	50	47.23
site_290	chr3	187528118	187528446	TCCT	80.8	82	78.69
site_291	chr3	190960036	190960332	AAGA	71.8	73	70.48
site_292	chr3	192188150	192188393	GAAA	58.0	60	56.01
site_293	chr3	192782342	192782573	AGAA	56.5	58	56.1
site_294	chr3	193823521	193823765	GAAA	58.0	59	56.82
site_295	chr3	197008938	197009158	GGAT	54.8	55	52.16
site_296	chr4	4667251	4667481	ATCT	56.8	58	55.31
site_297	chr4	5675762	5678135	ATAG	591.0	593	588.2
site_298	chr4	7154186	7154592	CATC	100.6	102	98.13
site_299	chr4	13212543	13212752	AAGA	50.8	51	48.37
site_300	chr4	13286732	13287084	GAAA	84.2	84	81.84
site_301	chr4	22163249	22163551	TAGA	75.0	74	73.24
site_302	chr4	34549779	34550032	CTTT	60.8	62	58.98
site_303	chr4	61005345	61005684	TTTC	82.5	85	81.66
site_304	chr4	77939103	77939367	AAAG	64.5	65	63.07
site_305	chr4	102149924	102150125	AAGA	49.0	51	47.27
site_306	chr4	106889086	106889330	TCTT	59.5	60	58.41
site_307	chr4	112706375	112706676	AGAA	73.0	75	72.29
site_308	chr4	121143374	121143577	AGAT	50.2	51	49.31
site_309	chr4	141218984	141219187	GAAGG	38.8	41	37.7
site_310	chr4	152079140	152079438	CTTT	71.5	70	70.41
site_311	chr4	154129063	154129591	AAAGG	104.0	103	102.92

site_312	chr4	156568205	156568527	TCT	105.0	106	101.42
site_313	chr4	159359253	159359502	AAGAAG	41.0	41	40.22
site_314	chr4	163761058	163761286	ATCT	56.2	58	54.23
site_315	chr4	168211992	168212194	AAG	65.7	68	63.91
site_316	chr4	175835064	175835291	TTTC	55.2	55	54.42
site_317	chr4	176361429	176361633	AAGG	50.5	51	48.83
site_318	chr4	183586793	183587093	TTTC	70.8	71	67.55
site_319	chr4	189326187	189326413	TTTC	55.2	55	53.98
site_320	chr5	5935710	5935932	GAGT	54.0	56	53.36
site_321	chr5	9292860	9293451	AAGGA	116.4	121	115.6
site_322	chr5	13981684	13981971	GAAA	67.8	69	66.91
site_323	chr5	20097141	20097448	AAAG	75.0	76	73.5
site_324	chr5	27219134	27219496	GAAA	89.0	88	87.19
site_325	chr5	44941893	44942826	AAAG	230.8	231	227.37
site_326	chr5	54000032	54000287	TTTCC	50.8	50	49.36
site_327	chr5	55820114	55820316	TAGA	50.2	52	47.31
site_328	chr5	56029951	56030255	AAGA	72.2	72	71.07
site_329	chr5	57859279	57859493	TCTT	52.2	53	51
site_330	chr5	60486957	60487166	AAG	69.0	68	68.3
site_331	chr5	79664034	79664864	GGAAG	149.6	167	148.11
site_332	chr5	93485067	93485322	AAG	83.8	85	81.83
site_333	chr5	106438242	106438449	GAAA	48.2	50	46.61
site_334	chr5	117963392	117963626	TTCT	56.5	58	55.05
site_335	chr5	128014673	128014961	AGAA	68.8	68	68.17
site_336	chr5	141800272	141800537	AAAG	65.0	65	63.55
site_337	chr5	143993064	143993342	TTTC	67.8	68	66.38
site_338	chr5	144505041	144505403	TTTC	86.2	89	84.88
site_339	chr5	155117418	155117630	AGAAG	40.8	43	40.04
site_340	chr5	171373699	171374526	ACC	273.2	275	270
site_341	chr5	172597176	172597391	GAAA	51.8	53	49.37
site_342	chr5	173518649	173518851	GAAA	46.5	46	45.82
site_343	chr5	174056997	174057204	TTTC	49.6	51	48.09
site_344	chr5	174324176	174324388	AAAG	50.8	51	49.1
site_345	chr5	174654536	174655438	CTAT	223.8	231	221.09
site_346	chr5	174699964	174700169	TCCA	50.2	51	48.77
site_347	chr5	176919424	176919728	CATC	75.0	76	73.54
site_348	chr5	177869991	177870193	AAAG	48.8	49	47.77
site_349	chr6	12216418	12216711	TTTC	71.5	70	70.02
site_350	chr6	14998259	14998549	AAGA	69.2	71	68.32
site_351	chr6	15971741	15972495	AAG	248.7	249	245.55
site_352	chr6	19216600	19216854	AAAG	62.0	63	61.61
site_353	chr6	23900153	23900629	TTC	154.7	157	151.27
site_354	chr6	32095598	32095837	TTCC	59.5	60	56.91
site_355	chr6	33315034	33315253	AAAG	52.5	53	50.2
site_356	chr6	48795427	48795789	GAAA	87.8	90	85.37

site_357	chr6	51222433	51222652	AAAG	51.8	53	49.55
site_358	chr6	51828261	51828721	AAGG	114.2	116	112.15
site_359	chr6	52172041	52172320	GAAA	68.0	67	66
site_360	chr6	53505884	53506085	AAAG	48.8	49	47.06
site_361	chr6	101586297	101586549	AAGA	61.4	62	59.95
site_362	chr6	126449243	126449450	AAAG	50.2	50	49.72
site_363	chr6	128306731	128306945	AAAG	51.8	53	48.01
site_364	chr6	132873365	132873578	CTTT	52.0	52	50.76
site_365	chr6	137644858	137645064	AAG	67.3	67	63.48
site_366	chr6	140281521	140281744	CTTT	54.0	55	51.18
site_367	chr6	156220639	156220859	CCCTC	41.2	43	40.76
site_368	chr6	156943079	156943303	TTCC	55.5	55	52.72
site_369	chr6	160379157	160379450	TTTC	71.2	72	69.76
site_370	chr6	167311334	167311536	AAGA	48.8	51	48.21
site_371	chr7	4507883	4508109	AAAG	54.5	56	52.71
site_372	chr7	5008706	5009155	CTAT	111.5	112	111
site_373	chr7	13334154	13334667	TTTC	128.5	130	128.59
site_374	chr7	28248194	28248417	AAAAGG	36.2	40	35.6
site_375	chr7	30624493	30624701	CTTT	50.8	49	50.01
site_376	chr7	43849323	43849585	AAAG	64.2	65	62.42
site_377	chr7	75049532	75049787	CCTT	62.5	61	60.91
site_378	chr7	82489652	82489920	CCTT	65.5	64	63.99
site_379	chr7	85940754	85941102	GAA	112.2	115	110.15
site_380	chr7	91897533	91897790	TTTC	62.6	65	62.27
site_381	chr7	96751312	96751587	TTTC	65.2	67	63.92
site_382	chr7	102809559	102809894	TTTC	79.2	78	77.82
site_383	chr7	105928908	105929116	CTCTT	39.4	46	37.96
site_384	chr7	107047682	107048515	TCCA	206.9	211	205.17
site_385	chr7	112599435	112599652	AAAG	52.8	54	51.48
site_386	chr7	127265423	127265822	AAGA	97.2	100	96.12
site_387	chr7	152705910	152706116	TCTT	49.2	49	48.34
site_388	chr8	10460595	10460851	AAGGA	50.4	51	48.27
site_389	chr8	14968586	14968845	GATA	64.0	65	62.06
site_390	chr8	19366984	19367299	TCTT	77.4	80	75.33
site_391	chr8	19931728	19932085	AGAA	88.2	88	86.64
site_392	chr8	25871417	25871667	TTCT	59.6	61	60.2
site_393	chr8	38833570	38833837	TTC	86.7	90	85.27
site_394	chr8	41159950	41160197	AAAG	60.0	61	58.28
site_395	chr8	47024457	47024694	AGAA	57.0	60	54.89
site_396	chr8	53383784	53383999	TTCT	51.6	51	49.61
site_397	chr8	60743023	60743325	AAAG	74.9	75	74.4
site_398	chr8	64576270	64576472	AGAT	49.8	50	48.67
site_399	chr8	69881657	69881966	GAAGG	58.4	63	58.55
site_400	chr8	70214411	70214634	AAAG	53.9	56	52.12
site_401	chr8	74510228	74510507	TTCT	67.5	68	65.95

site_402	chr8	75357983	75358197	TCTT	52.0	52	50.38
site_403	chr8	86003055	86003297	TCTT	58.6	61	57.02
site_404	chr8	95732005	95732217	AAAG	51.8	52	50.67
site_405	chr8	97375949	97376394	GAA	145.3	144	141.14
site_406	chr8	101879065	101879280	TCTT	52.8	53	51.98
site_407	chr8	109226264	109226713	AAAG	108.1	109	107.07
site_408	chr8	118888159	118888373	AAAG	52.8	53	51.1
site_409	chr8	125357209	125357413	AGAA	48.0	48	46.72
site_410	chr8	125998461	125998680	TTC	70.7	71	68.83
site_411	chr9	20032919	20033137	AAAG	52.2	53	51.35
site_412	chr9	21138116	21138315	TTC	63.3	65	61.11
site_413	chr9	47994698	47994911	AAAG	50.8	53	48.25
site_414	chr9	81824818	81825047	CTTT	55.5	55	53.57
site_415	chr9	82638327	82638538	TTTC	51.5	51	49.12
site_416	chr9	95293886	95294100	AAAG	51.2	51	50.03
site_417	chr9	96545199	96545411	CTTT	51.2	52	50.21
site_418	chr9	103788230	103788531	AGAA	73.0	74	72.57
site_419	chr9	127142153	127142488	TTTC	80.8	82	79.62
site_420	chr9	131120412	131120671	TTCTA	51.2	50	50.55
site_421	chr9	131278538	131278743	TTTC	50.2	51	48.89
site_422	chr9	147011448	147011797	CCAT	86.1	87	83.64
site_423	chrX	570950	571318	TTCT	88.8	89	86.42
site_424	chrX	880870	881145	AAGA	66.8	67	65.58
site_425	chrX	3665196	3665541	CTTT	82.2	82	80.55
site_426	chrX	4680125	4680383	TCTT	63.2	64	61.83
site_427	chrX	5341943	5342315	GAAA	89.5	93	88.34
site_428	chrX	5356029	5356310	AGAA	66.0	67	64.01
site_429	chrX	11776309	11776518	AGAA	50.0	51	48.08
site_430	chrX	23948398	23948618	CTGCTG	35.5	36	34.27
site_431	chrX	24653742	24653986	TTC	80.3	82	79.58
site_432	chrX	50892729	50892998	TTC	88.7	89	88.55
site_433	chrX	67337257	67337569	CTTT	74.5	75	73.76
site_434	chrX	85632401	85632642	AAAG	58.5	57	56.42
site_435	chrX	104570045	104570287	AAAG	58.5	59	56.99
site_436	chrX	106347502	106347929	TTTCC	84.2	85	83.05
site_437	chrX	118957353	118957601	AGA	80.8	83	78.39
site_438	chrX	122100371	122100617	AAGA	60.2	62	59.46
site_439	chrX	143432030	143432323	AGAA	70.5	71	68.89

Table S7. Pairwise concordance between tools (CHM13, 439 haploid loci)

Tool A	Tool B	n	Pearson R	Spearman R	MAD (copies)	RMSE (copies)
RepeatHMM	Straglr	437	0.998	0.984	1.72	2.975
RepeatHMM	HMMSTR	439	0.9974	0.9818	2.303	3.703
RepeatHMM	STRiker	439	0.9979	0.9813	1.696	3.072
Straglr	HMMSTR	437	0.9995	0.9958	1.195	1.719
Straglr	STRiker	437	0.9999	0.9979	1.66	1.82
HMMSTR	STRiker	439	0.9994	0.9935	2.565	3.008

Table S8. Accuracy vs leave-one-out consensus (median of the other three tools) (CHM13, 439 haploid loci)

Tool	n	MAD vs consensus	RMSE vs consensus	Bias (tool - consensus)
RepeatHMM	437	1.604	2.9	-0.727
Straglr	437	1.029	1.272	0.693
HMMSTR	437	1.773	2.308	1.72
STRiker	437	1.705	1.982	-1.682

Table S9. Bland-Altman: STRiker vs each tool (signed difference STRiker - other) (CHM13, 439 haploid loci)

Comparison	n	Mean bias	SD of diff	Lower LoA (95%)	Upper LoA (95%)
STRiker - RepeatHMM	439	-0.78	2.97	-6.61	5.04
STRiker - Straglr	437	-1.66	0.76	-3.14	-0.17
STRiker - HMMSTR	439	-2.54	1.62	-5.70	0.63

Table S10. STR genotyping outputs across analytical tools for the NA12878 reference sample

Gene	Known Motif	RepeatHMM	Straglr	HMMSTR	STRiker
ATN1	CAG	19	16.7	17/20	8.05
FXN	GAA	14	13.3	11/13	4.46
ATXN1	CAG	no reads / below MinSup	no motif detected	28	19.54
ATXN2	CAG	no reads / below MinSup	no motif detected	22	7.41
ATXN3	CAG	no reads / below MinSup	20	20	9.82
CACNA1A	CAG	no reads / below MinSup	11.2	1/11	-8.6
ATXN7	CAG	11	9.7	10	-0.38
ATXN8OS	CAG/TAG	no reads / below MinSup	no motif detected	17/20	12.27
TBP	CAG	54	35.5	36	28.16
HTT	CAG	no reads / below MinSup	17.3	19/23	15.33
FMR1	CGG	47	45.7	46/51	41.05
C9orf72	GGGGCC	no reads / below MinSup	3.4/9.8	8/11	5.06
RFC1	AAGGG/ACAGG	no reads / below MinSup	no motif detected	8/11	4.81
ATXN10	ATTCT	13/15	12.8	13/16	9.84
PPP2R2B	CAG	no reads / below MinSup	5	10/13	-1.09
BEAN1	TGGAA	no reads / below MinSup	7.5	5	0.25
NOP56	GGCCTG	6	6.7	6/7	1.42
DAB1	ATTTC	no reads / below MinSup	no motif detected	14/17	10.84
GLS	GCA	13	12.7	8/14	2.02
FGF14	GAA	676	10.7	1	659.83
CSTB	CCCCGCCCCGCG	no reads / below MinSup	4	2	-1.45
ZFHX3	GCC	21	18	21	4.5
NOTCH2NLC	CGG	13/19	13.2	13/18	2.36
PRNP	CCTCAGGGCGGTGGTGGCTGG	no reads / below MinSup	no motif detected	1	-1.42
ARX	GCC	14	12.7	14	2.82

SAMD12	TTTCA	no reads / below MinSup	no motif detected	22	17.67
STARD7	ATTTC	no reads / below MinSup	no motif detected	11	5.78
MARCHF6	TTTCA	13/15	no motif detected	14/16	9.58
TNRC6A	TTTTA	13/16	13.2	13/16	4.96
RAPGEF2	TTTTA	20	19.4	20	14.4
YEATS2	TTTCA	13	5.7	8	79.8
JPH3	CTG	15/17	14.7	14/17	8.05
AFF2	CCG	no reads / below MinSup	44.3	47	32.8
SOX3	GCG	12	13.7	12	-3.15
DIP2B	CGG	16/17	7.7/15.7	7/15	0
ZNF713	CGG	no reads / below MinSup	9.3	56/62	8.27
CBL	CCG	6/7	2.9	11/12	-3.32
AR	CAG	no reads / below MinSup	31.7	32	20.77
PRDM12	GCG	no reads / below MinSup	10.5	12	-1.09
WDR7	NNN				
NIPA1	GCG	7	11.7	8	-5.85
VWA1	GGCGCGGAGC	no reads / below MinSup	2.2	2	-1.61
DMPK	CTG	no reads / below MinSup	no motif detected	5/13	-3.77
NUTM2B-AS1	CGG	15	14.3	17	6.21
PABPN1	GCG	33	11	12/13	2.93
CNBP	CCTG	no reads / below MinSup	no motif detected	13	9.92
LRP12	CGG	no reads / below MinSup	4.8	11/16	0.96
GIPC1	CGG	no reads / below MinSup	4	22/24	9.76
PLIN4	NNN				
ZIC2	GCG	14	13.7	15	1.25
XYLT1	GGC	no reads / below MinSup	8.5	104	102.61

FOXL2	GCG	no reads / below MinSup	2.5	13	7.63
HOXA13	GCG	7	6.7	41/44	34.45
HOXD13	GCG	13	15.3	14	-0.93
PHOX2B	GCG	no reads / below MinSup	no motif detected	14/16	0.88
TCF4	TGC	no reads / below MinSup	no motif detected	17/24	8.95

WDR7 and *PLIN4* were included in benchmarking but excluded from the results, as the repeat expansion motifs at these loci have not been well characterized.

Table S11. Computational efficiency comparison across analytical tools for CHM13 STR genotyping (mean ± SD)

Metric	Threads	RepeatHMM	Straglr	HMMSTR	STRiker
Wall clock mean (s)	1	956.7 ± 12.1	1484.23 ± 66.53	33912.7 ± 282.39	3826.33 ± 165.63
Wall clock mean (s)	16	-	338.47 ± 55.73	8510.67 ± 639.93	1153.77 ± 272
Peak memory mean (MB)	1	121.6 ± 0.2	613.77 ± 0.6	15432.9 ± 3.71	2591.47 ± 31.45
Peak memory mean (MB)	16	-	462 ± 27.06	15429.6 ± 3.02	2532.17 ± 16.98
CPU utilization mean (%)	1	95.3 ± 1.2	99.67 ± 0.58	100 ± 0	111.67 ± 1.53
CPU utilization mean (%)	16	-	1383 ± 78.08	1493 ± 112.62	938.33 ± 204.85
Time per locus mean (s)	1	2.179 ± 0.027	3.4 ± 0.17	77.27 ± 0.6	8.73 ± 0.4
Time per locus mean (s)	16	-	0.77 ± 0.12	19.4 ± 1.48	2.63 ± 0.58

Table S12. Computational efficiency comparison across analytical tools for NA12878 STR genotyping (mean ± SD)

Metric	Threads	RepeatHMM	Straglr	HMMSTR	STRiker
Wall clock mean (s)	1	34.1 ± 1.6	31 ± 0.92	729.87 ± 3.11	60 ± 1.95
Wall clock mean (s)	16	-	9.07 ± 1.59	222.9 ± 0.66	23.8 ± 0.1
Peak memory mean (MB)	1	126.9 ± 0.1	293.53 ± 0.55	15564.63 ± 0.32	330.27 ± 0.55
Peak memory mean (MB)	16	-	244 ± 2	15562.8 ± 7.21	331.33 ± 4.65
CPU utilization mean (%)	1	85.7 ± 3.2	100.33 ±2.31	104.33 ± 0.58	199 ± 6.08
CPU utilization mean (%)	16	-	779 ± 49.76	1267 ± 2.65	722.33 ± 4.16
Time per locus mean (s)	1	0.632 ± 0.03	0.6 ± 0	13.53 ± 0.06	1.1 ± 0
Time per locus mean (s)	16	-	0.17 ± 0.06	4.1 ± 0	0.4 ± 0

Table S13. All crRNAs for nCATS determined through in vitro cleavage

Gene	crRNA sequence
*ATN1*_forward	AGGAGGATAGGCTTAAAAAC
	ATAGACAGATACGGATGGAT
	AATGAATACAGCTGGCTATA
	GAAGAGCGCACACACATCAG
	GAGCTAGCAGCACTTAATTC
*ATN1*_reverse	TGGGTGAAGGGATCCAAGCC
	TGTCACCAGAAGGCAGGTTT
	CCGGGTAAGGTGAGACCCTG
	TCGTTCTCGTGCAGAGGGTG
	CTAAAACTCGTGCTTCTCTT
*FXN*_forward	ATATCAACTTTGCAGGGAGC
	CTTGACTACCTCCAAGGAAG
	CAACATCTTTGCCCACGCCA
	AGGCTAAAAGCAACCAAGAG
	AATATCCTAATGGCAGAGCA
*FXN*_reverse	*ATCAACCAAATGTCACCTAA*
	TCAGGACCCATACCTCGCAG
	AGGTGTCCCCTTGTCACCCC
	GCCTACCCCCTGGTAAGGAC
	AAGTCTCAGGCCCAGTGGTC
*ATXN1*_forward	GAACAGAAGAGGGCTGTAAG
	CTACCTGCTCGTCTGCACCC
	AGCACACTTGCCTTTCTTTG
	GACAAATCATTGGTTGCGTC
	GACTCTGAACCTGTCACTTG
*ATXN1*_reverse	ACCAGTCCAGTGAGTCTTCA
	TTGTGTCCTTGATTTCTCAT
	CTGTCTGCTCCGTGCTGCAC
	ATCAGTACTGAAAGCTTACG
	AGGAATGTGGTGCCATCCTG
*ATXN2*_forward	CCGCACTCATTGCAACCTCC
	TAACACGGCTGTAGCTATTT
	CCTTAGCTCATGAGGCTGGG
	TGAGCCCCACCTGTTAGATC
	AAACAAGCCTGTTAGGGGGC
*ATXN2*_reverse	AACTGAACGAGACAGGGTCT
	CACCCAACAATTTCGTTTGT
	ATCATTTGGGACCGCCATAG
	AGGATTGCTCGGGACCGCCT
	ATGTTCACTTGTTGGTTGGG

*ATXN3*_forward	AGATGCTATGTCATATGGTC
	GGACTAAATAATGATCTTCC
	ACTACAGTTTGTTTCACTGT
	TACCCACTTTCTCTAACTAG
	CACTCATAGCATCACCTGTT
*ATXN3*_reverse	CACCCTTCCCAAATTGACCC
	AGGTAGTTCGTTGTCTCTCT
	TGGTGGAGGATTGATTGAGG
	TCTGAGGATGTCAGATTATT
	TGCAAATACTGGTGAGAATT
*CACNA1A*_forward	ATCCCCCCGTCTCCTTACCA
	GGGTTCCAGTCCTGGAAGCT
	CATTGGTATCTGTAAGAGAC
	TTGCCCTGAGTTCCTGAGGC
	TAAGAAGGTGACATAAGGCT
*CACNA1A*_reverse	CAGTCTCTGAAGCAGTCTGC
	ACTCTGCACTGGGTAGGGCC
	GAGCACTACCTCCCCATGGA
	TCCGTGGAGATGCGAGAGAT
	ACACCAGGGATCTGGAGGTC
*ATXN7*_forward	CCAATTGAAGACGGTTGTGT
	CAAATGACTTCAGTCCTTTG
	GTGAGAGAGGATTTGAACAA
	GCTCTCTCTGGAGAAGAACG
	CACTAGAGGGAGCATAGTTA
*ATXN7*_reverse	GAATTTCACAAATCACCTTG
	GGGAACTTACTCTCTTAACA
	CGAAAAGTGCTGAAACCTTC
	ATCAAGTGTGGACATCACAC
	CCCCAGGCTCAATCACGTTC
*ATXN8OS*_forward	ATCCTATTTCATTGACCACA
	ACACGTCAGATCTTTAGTTC
	TCTTGGGATGGAAGTGTTTC
	GGAAAAGATATCCCCACACG
	TCTGAAGTACCAAAATCTGA
*ATXN8OS*_reverse	AGTTCTCTCTAATTAAGCAC
	TCCATACTCCCAAACTTAAA
	TTATGCGAAATGTAAACGGA
	GACAATTAGAGCCACATTAC
	GGACTCTATGCCAGGTTATT

*TBP*_forward	CCCCTTTATAGTCACTCTGC
	CCTCAGGTAATATAGCAGGA
	AGTTCCAGCGCAAGGGTTTC
	GAAGATAACCCAAGGAATTG
	TAGCCATAGAACTGGCTTAT
*TBP*_reverse	GGGGAAAATATTTCAACTGT
	AAAAATCACAACTGGTAGGA
	ACCGCAGCAAACCGCTACAA
	AAAATCAGTGCCGTGGTTCG
	AATTTCTTACGGCTACCTCT
*HTT*_forward	CACAAGTGCTCATCTGGAAC
	GGACGTTATCAGGCTCTACA
	ACTGCTATGACTTGGTGACT
	CTTCTCGGCAGGACAGGCAC
	ATCCCACCTGGTCTCGGGTC
*HTT*_reverse	CTAGACTCTTAACTCGCTTG
	TTGGACCTGTTCCCCCCATC
	CAAGTAGTATTGGTCTGATA
	CAGAGTTCAACAAGTGCAGT
	CCAGTTTAAGCTATTGCAGC
*FMR1*_forward	AGGTAGACCAGATGAATAGG
	GTTGTTCCTCAGTATCATGG
	AGGCAATGCCCTCAAATATT
	TTCTGCTGACACTGTAATGG
	TAATAACAGGAGGAACTGTA
*FMR1*_reverse	ACAAAGACAAATTTCCTAGC
	ACATGTCCAGGACTTCATAT
	AACCTGCAGGAGACAGGATA
	CAACAGGAACCACTGCTCAC
	CAGAAAGTCCATCCTTATTT
*C9orf72*_forward	ACAGTGGTGTACAGTGTTCT
	AGCTAGAGACTGACACTTGT
	TTCTAGTATGACTGGAGATT
	GTCCTCTTAAGTCAAAGATG
	GTCACATTATCCAAATGCTC
*C9orf72*_reverse	ATCTACATCCTAGAGATGTC
	GGTACCAGGTTGTTGTCCTC
	ATGCTAGTATTAACCACCAT
	TGGAGTTGTATTCCCCTCCC
	TACCTGTTGGATTAGGTGGG

*RFC1*_forward	TACCACAGCCTAATCTCCCA
	GCCCTCACGTTGCCATCTCC
	GGGCCAGTCAAGGTACCAGC
	ACTCAAGAACATAACACACA
	GTAATTGGTCCTTAATCTAC
*RFC1*_reverse	GTATTAGTCCCACTTTACTG
	GATAGTTCAGAAATGAGGTG
	GGTTCTTAAATCCAGTTCTT
	TGATTACAACCATCAAGGAT
	CTTCAGAGCAGGTGGATTAT
*ATXN10*_forward	TGTGATATCTGAGGGAAAGA
	GTCTCTTGCCGGAATGTGTG
	CTTAATGCCTGTATAGGGTC
	CTTGGACCGCTGGTGAGGAG
	GTAGGAGTGTGTCCTTCCTT
*ATXN10*_reverse	*TCTTCTCATCGAGTTGTAAG*
	GTCCGTTCTATAACACAAAT
	AATGCCTGGGCAGCAACATA
	CATCATGAGACCACAGGAAA
	GTGTACAGTGAGGACTGTAT
*PPP2R2B*_forward	AGACTGCTATAATGGAGATA
	CAGCTATAATGAATTAAGGG
	TTAAAAAGGCTTCTGAGGGA
	GACAGAGACCACGTCTGTAT
	CTTTCCACCTTATGACCCCA
*PPP2R2B*_reverse	GTAAAGCACAAGCCTGAAAT
	CTGAACTGGGTAACATAAGA
	AGGAAGCATTAGCCTAAGGA
	AAGAATCTCCAGACTGGCTT
	GAGATCTCATGAAGTACCGC
*BEAN1*_forward	GAGCAAGGGGGATTCAGCCA
	GGTGCATGCACTAGAGTTCC
	GAAGTGGCCCTTGCTCTGGG
	GCAAGCAGCCCTCACCCACA
	ATGAGCAGGTCCCAATCCCC
*BEAN1*_reverse	GTGCCCTGGGGGATATGCCT
	TTGGCCTAAGTTGCTCTCCC
	ATTTACCCAGATGGGGAATG
	AGAGAGATATATGTTGGTCA
	AGGAGCTAGGAATTGGATGA

*NOP56*_forward	AAGTCTACCTCAGGAACACT
	CTCATCCTGACTTTCATTCA
	CTGCAGGACATTTGCTCAGT
	TGTGGAACCTGGAGCACATC
	GACACAGTTAGCCATAGGCT
*NOP56*_reverse	TTCTCCCCGAATACACTCGT
	GTGCTCTGAGGACTCCTTTC
	CTACTGACCTCACACTCCTC
	CTCCTTCCTATAGAATCAAG
	TGAGCTACAACCAGCTCTCT
*DAB1*_forward	CTGAATCCACACTAGTGAGA
	TCTCCATGCCTCGGTTGTCC
	GGATGTGGGATACTCGACGG
	GTATTGGCTGAACGGCTACC
	AGCCCCAGTAAATATTGAGT
*DAB1*_reverse	CTATCTTTAAACTACACAGC
	TGATTAACCACTGATATGGT
	ACCTTAGACCTTTGAGGAAG
	AGAATCACATAGCCCAGATG
	AATATTCTGTGCTCCATTGT
*GLS*_forward	TATGATGTCACAATGAACAG
	GGAGGTAAAGTCTGCCCAGT
	AGTGAGTTTATGCTCGGCAG
	GGAAAGAAGCGTCCCCTCTC
	GGTAAAGAAGCATCCCCTCA
*GLS*_reverse	TGAAATTCAATGTTCAGACC
	GGATTAGCTGACTTTGAGGT
	TTGCACCTGAGTAGATTTCA
	TGCTTCTACTACGTTGTAGG
	ACACTCCCAAATGGAAGTCA
*FGF14*_forward	TTTGAACTCCCAATCTAGCA
	AATAACCTCTTACTACACCT
	CTGGCTTATATCAGCAAATC
	ACAGCTTGGGTGGAGCTGCT
	CTTACCTTTGTGCAGTGATG
*FGF14*_reverse	CTGCACTTGTCTGTTAATGA
	GCACGCATCTGCTAGACGAC
	AAGCGCTTAAACACTATGCT
	TTTCCCTGTAAGGGACAGCA
	GCTAGGTTCTTGTGAATCTC

*CSTB*_forward	TGCTGCTGTTGATGCTCCTA
	GGTTCGGTTAAACCAGCGGG
	GAGACGTGCCAGCAAAAGCG
	GGACCCTCCCTTAGGGCACT
	TCAAGCCTCAGGTCTGAACT
*CSTB*_reverse	CATTAACTGCAGGACTAACT
	TTGCTGCTGACCGAGTGGGT
	CATTGCAGGCCTGTGGGTTA
	ATGGGAGGTAAAAGTCAGCA
	ATTCTCTAAGTGGGGAGACA
*ZFHX3*_forward	ACTCAAGAAGACAGATTCAG
	GAAGGTCATTAGTCTGTAAC
	GCCCTTCCTTAATGTAACTT
	GCTGCAGAAACTAAGAAAAA
	GATACTGCTGTTGTGCAGAC
*ZFHX3*_reverse	TCTTATAGTCTCACTTAATA
	TGTGGTAGATCCTTAATGTT
	TTACTCCATCCAACACAGGT
	GTTGATGGCTCAACAAGAGT
	CAAAGAGAGTCGTTCAGGTC
*NOTCH2NLC*_forward	GGCTGTCTTTGAAATAAGAT
	CCTTCAGTTGCAGAAACTAA
	TTCTAGAACTTGTCCTGTGT
	GCAAGAGAGGGGAGGGTGTC
	CTTATGTTTCCTAGTTACAG
*NOTCH2NLC*_reverse	TACATGAGATGGAAGCAATG
	CTCAGTTGACTCTGAATTTC
	ATCCGAGTCACCCTGACTGC
	CCTAATAAAGGCCAAACTAT
	ACTGCCTAGCCAGAGGACCC
*PRNP*_forward	TAAGCTGACACATACTGGCT
	GGGTCACCTATATATAGCTG
	CCACCTGCCACTGTCTCATG
	GTCCTACTAAGACCCTTTAA
	ACTCAACCAGCAGGTCTTAT
*PRNP*_reverse	GATCCTCTGATCACCGTAGG
	CAGGTGGATATTATTCACTC
	ATTAAGGCTCCCCTTAGAGT
	TTAGATCAGTCGGCAAGGTC
	TCAGGATGAGCTGAGTCCTA

*ARX*_forward	CGGAAATGCACTCTGGTATG
	TTCACTCAGCTCCTAACTCG
	GTCACATCTCTGTTTGACAA
	CATCTTCTCCAGTGAATAGG
	ATACCTCTGAGGCCCCCCAA
*ARX*_reverse	GTGTAAAAACACCGAACTCT
	TGGAATGGGCTTTCGGGGCC
	CTCCCAACCACCAAGCCGCA
	TCTTGTGGATTTCCAACGCG
	CAGAGAGCTGAAACTCCGTT
*SAMD12*_forward	GTGATAGAGCACTTTGGTGG
	CCTACTAGAAAATTCAAGCC
	AGTCTGTACATTCTCCTTTG
	ATATGACCTAAATGTTCTGG
	GAACTAGTGTGCTGGCCTTC
*SAMD12*_reverse	ACTCACGCTGTCACCTGTCA
	GGGTAGGTCCTCTTAGTTGT
	AATGGCCTCTCTCAGGCTGT
	GGAGCCATCAGTATCATTAG
	TGCCATAAGCACTCACACAT
*STARD7*_forward	AGGAACTCGGCAACAGCCAC
	TAGCTGGATGGAAGGGGCAC
	TTTACGGCTCACTAGGCTGT
	GGAGAGCTAAGTTAAGGGGA
	GCTCCACAGCACTGGTAAGG
*STARD7*_reverse	TACCAGGACATGCTAGGAGC
	GGGGCTGCATTCACCTATTT
	TGGCATTCTTTCCCCCAGAT
	TATTGACAAGGCCAGGGTAC
	TGTAACAAAGAAACTGGTCT
*MARCHF6*_forward	GAGTTGGATCAGAAGGAGAG
	GCCAATACGTGCTCTGCTAC
	GAGGGTCACATACCTGCCCT
	GGAAGGCACCTAAATACAAA
	GTCTGAATGCGAAAAGGTTA
*MARCHF6*_reverse	GTCCCCTGAACAAGAGTTAG
	ACCCAGCTACCTGGATCAAA
	AGTGATCTTCATAGTCTTAC
	GTAGACATGTACCCAATATT
	TGCAGAAAACATTGTTGCAC

*TNRC6A*_forward	GCAAAGGAGGTTACTACAAT
	TACGAGACCTGCAGCATGTT
	GCACCTCAAGAACAGAGATG
	TTGGGCAGAATAGGGGAGTG
	CAGTGCTTAGTATCAATAGT
*TNRC6A*_reverse	CTTACAAGGCACATGACTCT
	CAGGCGTAGCTGAAGAACAG
	ACATCTCCAGAACTGTGCAG
	TAATTACTCCTAACAGAAGG
	CTGAGAGCCTACCAGGAGTA
*RAPGEF2*_forward	ATGACAGCATAGTAGGATTA
	GTGTAACTTTGTTTAATCCC
	CGTGTCATGTATTACATAGC
	CTTAAAGGTTGAGTGCGCGG
	GAAAGGCAGTTCATCATGTA
*RAPGEF2*_reverse	GGAGAGGTCTCGGATTTGAC
	TGAAGGATAAAGGGACACGG
	GTAATCACTTACCAAAAGGT
	TTGCAAACCATGACAGTGAA
	GTTGTACCACTTCCACAAAG
*YEATS2*_forward	TAGTAATATTGAGCACTTCC
	ACATCTCTTAGGTCATGTTA
	TACACTCTTATTAAGATTAG
	TGTCTCTTCTAGAACTTAAG
	GAAATCATTATCAGCATCTT
*YEATS2*_reverse	GCTATAAAATACAACACTGG
	AAGACATTCTATAAAATTAT
	AAAACTGAAACCACTAATAT
	TATAGTCTATGTATATTCCC
	GCATAATGCTAACAATTAGG
*JPH3*_forward	CTGGAAGGCCAGGCAGACTT
	CAAGGCACTTTACGTTGGTG
	CTAGTGCCTGGCTCACATGT
	CTGGGTGGTACTACGTTTAT
	GCCAGCCCTACTTTATCGGG
*JPH3*_reverse	GAAGCTACCAGCGGGGGAAG
	AGAGATGGTTTGGCTTGAAG
	CTGGTGGTTGAGTGTGCTGG
	TGCACCGACTGAGTAGGACC
	TGCCAAGTGGCAGCCCTTCC

*AFF2*_forward	TACTCAGATTCTGTGAGCCT
	ATGATTGACATAAGGTCAGA
	ACTTTAGAAGAGCTCTGAAA
	ATTGCTACCTATACAGAAGC
	GGGAGAACTGAGATTGAAAT
*AFF2*_reverse	GCCGGGTTGCTGCAGTGCAT
	GATTGTAGCTAACGAGGCCT
	TAGTATCTCCAAGTTGGCAA
	GAGGGTTTGGGGAGTTGCGG
	CTACAGGATGCTTAAATGTA
*SOX3*_forward	GAATCAAGCTTTGGTCATTC
	ATTGTGGGAAGGTTAGGATT
	AACCTTCATTTGACCACATC
	CATAGCCCTAACTGTCAAAT
	GTCTCCACAAAATTGTTAGT
*SOX3*_reverse	CGATATGAAGGGGTAGAAGG
	TTGAGCAGAATAATATCCCG
	GACCCTTGAAAAGTCCAGTA
	GTGACATCTCTACTTAGAAA
	TGTGTATCCTGTCTAAATTC
*DIP2B*_forward	GACCCTTCGACTTGGATGCG
	AATGGCAATTACTATAAGAC
	GGGTGTTCCACCTTCCGGCA
*DIP2B*_reverse	GGATAGGTTTACAAAAAAGG
	GTGGCCCCGTTTCCCATGAT
	TGCTAAGTCTTAAACAGGCT
*ZNF713*_forward	TTCCTGCCTAAAAGGCTGTT
	ACACTCTCCAAAAGGGAAAG
	GGCTCTTGGATGTGGTGCTC
*ZNF713*_reverse	TGAGCTTCCCTGGTTCTGTA
	GAGTGGTCTTACCCCACACT
	CAAGCCCACATGACTGGTGT
*CBL*_forward	TCCTCATCTAGAAGCCTGTT
	CGCTTTGGTCTCCAAGTGTC
	AGGAGCTACCTTGCTTGGTC
*CBL*_reverse	GTACACGGTTCAGTACTATT
	GAGTCGTAACAACAACAACC
	AAGGCAAGGTGGATAGGGTA

*AR*_forward	GAACCAACATTGCAGGTAGA
	GACAGTGACAGGACTTAAAC
	ACTGAAAGCTATACAACTTC
	TGCTCTGCATCTTTATTCAT
	GTTCCATTGACATAAAACTC
*AR*_reverse	CAGCTCCATAAAATATCATC
	TAAACCCCAAACTCTAATTC
	CTTGATAACTGAGAGGAGAG
	TGGGCAGAAATAATCTGTCT
	GGTCCTCAGGTGTTGATTGT
*PRDM12*_forward	ACTCTAGGCTGAGTCTCAGG
	CAATGGTCTGAGGGTCTTCC
	AACGCCAGAGTTCTGAGTGT
	TGGTTTCAGACACCTCTAGA
	GGTGCCAAGGCCAGCACTTC
*PRDM12*_reverse	ATCAGAAAGGGCTGAAGGAA
	GATTCAAGAATAGTGGGCAT
	TGCACACAGATTTCAGCCTA
	TTCAATCCTCATCTACCTAC
	CAGAAAAGCTTGCCTGACAG
*WDR7*_forward	GATCTGATCAATTAGAAAGA
	CAACCTGGTATGCTACGTGA
	CTGAAATGTACACTACCTGT
*WDR7*_reverse	GTCTCTACATATACAAGGAC
	ATCTCAGCCTAGCTCCCGAG
	GGGTGGAAGTATTTCCCTCC
*NIPA1*_forward	GTGTACCACGTATCTCAGTT
	GCTAAGGGAGACGTTAAGTT
	TACTGGCCCCTGAATCTTTC
	ATACACTGCAAAGAGATCTT
	GCCTGTTAGGGGCCACAGCA
*NIPA1*_reverse	CTGCTGCTTGCAACAGATGT
	CTGGGTTAACCTGAAGGGAA
	AATCTAGAAATGCTACCACC
	GACGACGCTCCCTGGACTAC
	CTGCTATTCTAATGTTGCCA

*VWA1*_forward	CGGCGGAAGAGAAGCAAGGA
	TCTTCCCCCGGGTTCTTGCC
	CCCCTCCACTTCAGGACCCT
	GCGTTCTTTCCCGGTGGCTG
	CCCACCCCACTGGATGCAGT
*VWA1*_reverse	GCTCCTCCCTGAACAGTCCT
	AAGCCTTTGCGGACCTGACC
	GATTCCCAGTGGCTGCTAGC
	TTCCTGTCAGTGCGGTCCCG
	CAAGAAGATGGTGTGGTTGC
*DMPK*_forward	CCCTGCTGACCAGACAGGCA
	TGCCTAGGGCGTGAGCCTCT
	CCGTCTCTGGCTTCAGTGGC
	TGGGAGACGAGCATGCTGGT
	ACGATGGGGCTGCCAGACAC
*DMPK*_reverse	AAAACCTCAGAGTCACAGAC
	GACTCCTAAGAGGCCAGAGT
	TCCTGCTCCTTGGCAGCCAA
	TTCGGGAAGGTGCGCCGCTA
	CACATCTGTGTGTCTTGCGC
*NUTM2B-AS1*_forward	ACGAGGTCAAATGTCAAAGA
	AGGAAGCTGCTCTGACATTG
	AGGACTGCTGGATCATATAT
	GGCTTATCATGATATCCTCC
	CTCCATACTGTAATCCACAG
*NUTM2B-AS1*_reverse	CTCTAGTGTTATTCATTGTG
	AAATTCTTATGGCCATACCA
	CCCCCATATACTTTATTTCT
	AAGATGCAGGCTTGTTACAT
	TGTATACCTGTTAAGTGGTC
*PABPN1*_forward	AGTAAAGGAGAAAGACTCCA
	ACACTTAGCACATCTCAAGT
	CTCCATGGTCCTTTGTTTAT
	ATTCTCTATGCATCAACAAC
	GAGCACAGAGTGATAGGCAA
*PABPN1*_reverse	TCTGCGATCTAAGAGAGAAT
	GAGCACACAAAAGCGCCATT
	ATCCAAAGCTCTAGGGCTGC
	TTGAGGTAGAAAAGGTAGCT
	TGAAGTCTGAAGGTTCAACC

*CNBP*_forward	ACATGAGGCCCAGAATTTGG
	ACTGACAGAACCCTGCATGA
	ATCACACAGCAGCATGGAGG
	ATTTCGAAGTGGGGGGAAAC
	TATGAAACCCAGGAAAAGGT
*CNBP*_reverse	TGTGGATCCTAAATTCATCT
	GTAGAAGATACTTCTGTTAG
	GTAAATTTCTCAGCCACACG
	AGAGGTTCTGGCCTGGTTGC
	TAAGTGGTGCCTTATATTAG
*LRP12*_forward	AACACAGTTAGCTATGCTTT
	AGAAAGAGTACATATCTGAC
	ACTTAACTCTCTCAAAAAGC
	TAAAGACTTGCAGTAAGAAC
	TGTGGTATCACTGGTAAAGT
*LRP12*_reverse	TTGACTACTTGGTACAATGG
	TTATTGCATGCTCTATGGTC
	CCACTGGACTTAAGTATACA
	AAGGCTGCTGTTATATAGGT
	TAGGGTTCTCTGATCTTCAA
*GIPC1*_forward	TGACGCCAGGCACAAGATGC
	CAAAGTGGTGGTGCGGTGTT
	CTTATGTAAAAGGGACTTCC
	GGGCCATGCTGGATTCAGCT
	CTAGTGTGGTTGCCTCCTGT
*GIPC1*_reverse	TGTGGAAGAGTCAGGTGCGG
	GTAACACCATAGAATGGGCC
	AACCTCAGGTGGACTACGGA
	CTGCTGAACAGCACAGGTTA
	GACCGAGGCACAGAAGAGGA
*PLIN4*_forward	CGGCATCCCGCTCCTGTGGG
	CTCCTCACCATGGCCTTGGT
	CATATGCAGACGCGTCCCCT
*PLIN4*_reverse	AGGTAGCCATGTGACCGTCC
	CAGGACCGTGCCAGGTGCGC
	GGGCTCAGCACGAAGGTCCT

*ZIC2*_forward	CGCAGAAAGACTGGAGCGCT
	AAAGGGGCGGTTTGACCGGG
	ATCGAAATCAACGGAGGCGG
	CACTGCAAGTTTACTACGCG
	AACAATAACCGCCGCGCGCG
*ZIC2*_reverse	AGCTCTGGGACAGCCAGGTA
	TGTGATCTGCCCTTGATCTC
	CCTTCCTTCCTAGCCAACCC
	TGAGCTCACCAGGGACCTAC
	TCGGTGGAGTAGTGTGGGGG
*XYLT1*_forward	ATCATAAGTGGCAGCTCTTA
	TTATGGATCATAAACAGCTC
	TTATGTCAGAGATATGCCTA
	ATAAGAACTTACGCCATGGC
	TAAAGTATGAAAAGACCGGA
*XYLT1*_reverse	CTGGGGCTTCTTCAGAGCTC
	CGATGAAACAAGATTTGGGG
	AGAGAAGAACCCTTTCCCCC
	TTTGCACACTGACTCCGCTG
	TAGCAGAGCTGAGTAAACCT
*FOXL2*_forward	TGTCTGGCTGCATGAAGCCT
	GCTCATCTAAGCCTCAGATC
	TACCCTCTGGAGTAGGGTAC
	CCTTGGTGCATGTACTTCAA
	CCAACCCACTTACTCTGACT
*FOXL2*_reverse	ACTGGAACCGTATCCGCAGG
	AGATTGCTCGCGCCGGCTGT
	TTGCAGGATTCCCGGCCCTG
	GACGCTTCCCTAGTAGAGCT
	AACCTGGTTCAGGCAGCCGG
*HOXA13*_forward	CAACATTATCCATGGGTCAG
	CATGCCCACTTTAGGTTATC
	TTGGGAGAAAGTTGTTACTT
	GTACTGTTTGGAACAAGAGG
	TCGTTCTGTATCCAATCCTG
*HOXA13*_reverse	GATGCAAAGAAATAGTGCCA
	AGTGTCTTTAGAATCAACCA
	GTTAAGATGTTCTGCAAAAG
	CCAAATGATCTGCTCAAGTT
	GGGTACAGCTTCTACCTGAT

*HOXD13*_forward	GGACTCTGCGGAATGGAGAT
	CCGACAGAGTGAGAATTTAC
	AACAAGTTACTCTGGGCCTC
	AGATTAGTGCTTCTCTTCAA
	TTTCTGATTTGGAGGGGGTT
*HOXD13*_reverse	CAATCTCCCCTACCCACGAT
	TCCAAGCCCCCTTAGCCTTC
	CGCGGCGCTAAAAACCTGCC
	CTCTTGAATCACGCGACACG
	AGCCGGAGATCTTGGCGGCA
*PHOX2B*_forward	GAGCTTGTTCACACTGTGCA
	CTAAGGCTCCATCATATTAC
	GATGACCCAAATTAAAACGA
	AATGTAGTCCTCTGGGAAGC
	ACTCAAAGTTGCTCAAGACC
*PHOX2B*_reverse	CAGGGTTTGTTTACACCACA
	ACTTGTTACAGAGCACATAA
	GCAATAAAACACGGTGGCAG
	TCACCCTGCAAAGAAGCACA
	ACCAGGATCCTCGGTAGGTC
*TCF4*_forward	CATTGATATCTGTCATAAAT
	GTTTCCTCAGCTATGAATAC
	ACCAAGGTTATTTCCAATAA
*TCF4*_reverse	ATCATCTAAGCAGAACCTGT
	CCCAAACTTCATCCTATCAT
	GCTCCTGGGGTCCAATCTGC

Table S14. Selected crRNAs for nCATS determined through in vitro cleavage

Gene	crRNA sequence (forward)	crRNA sequence (reverse)	Size (bp)
ATN1	AATGAATACAGCTGGCTATA	CTAAAACTCGTGCTTCTCTT	8641
FXN	CTTGACTACCTCCAAGGAAG	AGGTGTCCCCTTGTCACCCC	8498
ATXN1	CTACCTGCTCGTCTGCACCC	TTGTGTCCTTGATTTCTCAT	8991
ATXN2	TGAGCCCCACCTGTTAGATC	CACCCAACAATTTCGTTTGT	12601
ATXN3	CACTCATAGCATCACCTGTT	AGGTAGTTCGTTGTCTCTCT	11304
CACNA1A	CATTGGTATCTGTAAGAGAC	GAGCACTACCTCCCCATGGA	10504
ATXN7	GTGAGAGAGGATTTGAACAA	ATCAAGTGTGGACATCACAC	9654
ATXN8OS	GGAAAAGATATCCCCACACG	TTATGCGAAATGTAAACGGA	9322
	ACACGTCAGATCTTTAGTTC	GGACTCTATGCCAGGTTATT	9198
TBP	CCCCTTTATAGTCACTCTGC	GGGGAAAATATTTCAACTGT	8371
	GAAGATAACCCAAGGAATTG	ACCGCAGCAAACCGCTACAA	9995
HTT	ACTGCTATGACTTGGTGACT	CAAGTAGTATTGGTCTGATA	9359
FMR1	GTTGTTCCTCAGTATCATGG	CAGAAAGTCCATCCTTATTT	11314
C9orf72	GTCACATTATCCAAATGCTC	TACCTGTTGGATTAGGTGGG	12688
RFC1	TACCACAGCCTAATCTCCCA	CTTCAGAGCAGGTGGATTAT	9801
ATXN10	GTCTCTTGCCGGAATGTGTG	TCTTCTCATCGAGTTGTAAG	9994
PPP2R2B	CTTTCCACCTTATGACCCCA	CTGAACTGGGTAACATAAGA	8770
BEAN1	ATGAGCAGGTCCCAATCCCC	TTGGCCTAAGTTGCTCTCCC	11374
NOP56	CTGCAGGACATTTGCTCAGT	CTACTGACCTCACACTCCTC	9473
DAB1	GTATTGGCTGAACGGCTACC	CTATCTTTAAACTACACAGC	8696
	CTGAATCCACACTAGTGAGA	ACCTTAGACCTTTGAGGAAG	9117
GLS	TATGATGTCACAATGAACAG	TGCTTCTACTACGTTGTAGG	10676
FGF14	CTGGCTTATATCAGCAAATC	GCTAGGTTCTTGTGAATCTC	13422
CSTB	TGCTGCTGTTGATGCTCCTA	ATTCTCTAAGTGGGGAGACA	9485
ZFHX3	GAAGGTCATTAGTCTGTAAC	TGTGGTAGATCCTTAATGTT	11202
NOTCH2NLC	CTTATGTTTCCTAGTTACAG	ATCCGAGTCACCCTGACTGC	11331
PRNP	CCACCTGCCACTGTCTCATG	CAGGTGGATATTATTCACTC	10286
ARX	TTCACTCAGCTCCTAACTCG	GTGTAAAAACACCGAACTCT	8150
SAMD12	ATATGACCTAAATGTTCTGG	GGAGCCATCAGTATCATTAG	11347
STARD7	TAGCTGGATGGAAGGGGCAC	TATTGACAAGGCCAGGGTAC	9389
MARCHF6	GTCTGAATGCGAAAAGGTTA	ACCCAGCTACCTGGATCAAA	8806
TNRC6A	TACGAGACCTGCAGCATGTT	ACATCTCCAGAACTGTGCAG	8723
RAPGEF2	GTGTAACTTTGTTTAATCCC	GTTGTACCACTTCCACAAAG	11512
YEATS2	TACACTCTTATTAAGATTAG	GCATAATGCTAACAATTAGG	12905
JPH3	GCCAGCCCTACTTTATCGGG	TGCCAAGTGGCAGCCCTTCC	8888
AFF2	GGGAGAACTGAGATTGAAAT	CTACAGGATGCTTAAATGTA	8957
SOX3	GTCTCCACAAAATTGTTAGT	TGTGTATCCTGTCTAAATTC	9292
DIP2B	GACCCTTCGACTTGGATGCG	GGATAGGTTTACAAAAAAGG	12954
	AATGGCAATTACTATAAGAC	GTGGCCCCGTTTCCCATGAT	9713
ZNF713	TTCCTGCCTAAAAGGCTGTT	TGAGCTTCCCTGGTTCTGTA	11467
CBL	CGCTTTGGTCTCCAAGTGTC	GTACACGGTTCAGTACTATT	9670

AR	ACTGAAAGCTATACAACTTC	CAGCTCCATAAAATATCATC	9239
PRDM12	AACGCCAGAGTTCTGAGTGT	CAGAAAAGCTTGCCTGACAG	10313
WDR7	CTGAAATGTACACTACCTGT	GTCTCTACATATACAAGGAC	9856
NIPA1	TACTGGCCCCTGAATCTTTC	CTGCTATTCTAATGTTGCCA	13014
VWA1	CCCACCCCACTGGATGCAGT	GCTCCTCCCTGAACAGTCCT	8290
DMPK	CCCTGCTGACCAGACAGGCA	CACATCTGTGTGTCTTGCGC	9306
NUTM2B-AS1	CTCCATACTGTAATCCACAG	TGTATACCTGTTAAGTGGTC	16227
	AGGACTGCTGGATCATATAT	AAGATGCAGGCTTGTTACAT	15116
PABPN1	GAGCACAGAGTGATAGGCAA	TGAAGTCTGAAGGTTCAACC	10948
CNBP	ACATGAGGCCCAGAATTTGG	GTAAATTTCTCAGCCACACG	9301
LRP12	AACACAGTTAGCTATGCTTT	TTGACTACTTGGTACAATGG	9613
GIPC1	CTTATGTAAAAGGGACTTCC	TGTGGAAGAGTCAGGTGCGG	9241
PLIN4	CGGCATCCCGCTCCTGTGGG	CAGGACCGTGCCAGGTGCGC	14136
ZIC2	CGCAGAAAGACTGGAGCGCT	CCTTCCTTCCTAGCCAACCC	9340
XYLT1	TAAAGTATGAAAAGACCGGA	AGAGAAGAACCCTTTCCCCC	8547
FOXL2	CCAACCCACTTACTCTGACT	ACTGGAACCGTATCCGCAGG	8330
HOXA13	TTGGGAGAAAGTTGTTACTT	GGGTACAGCTTCTACCTGAT	9327
HOXD13	AACAAGTTACTCTGGGCCTC	CAATCTCCCCTACCCACGAT	8255
PHOX2B	ACTCAAAGTTGCTCAAGACC	CAGGGTTTGTTTACACCACA	8171
TCF4	CATTGATATCTGTCATAAAT	ATCATCTAAGCAGAACCTGT	10746

Table S15. PCR primers targeting DNA fragments for *in vitro* cleavage assays

Gene		PCR primers (5'→3')		PCR product size (bp)
ATN1	front	forward	GGTGGGTGTTGTTCTTCGGA	2573
		reverse	TGTGGGCACCTGGAACTTTT	
	back	forward	GGTTTGGGTGTCATAGGCGA	2418
		reverse	CCCCCATATCCCGCAAAGAA	
FXN	front	forward	CAGGCAAAGGGATGGAAGACT	1757
		reverse	TGGAAGAAGTGGTGTTGGCA	
	back	forward	GCCTTGGGCAGCTTTTAGAC	2257
		reverse	AAACCTCACAACCGTGAGCA	
ATXN1	front	forward	AGTCTTTGGAAGGGCCCAAG	3573
		reverse	GCGACAGACGGCCCTAATAA	
	back	forward	TCTCCGAGGCTCCATCAACT	2220
		reverse	CGCTTAAAGACTGGGACGGA	
ATXN2	front	forward	CTCTTGCTAGCGGTGCTCTA	5791
		reverse	GCCAAGGAGGGCAAATTGTTT	
	back	forward	TGTCATCCCCAAATCCCGAG	3935
		reverse	GCCTGAAGCTGACATAGCTGA	
ATXN3	front	forward	CCTTGGAACCAGAGGAGCAG	5059
		reverse	ATAAGGCGCAGGAAGAAGGG	
	back	forward	AGGCAGGCAGATTACGAAGT	4497
		reverse	CTGCCCCTCCACTCAAGAAG	
CACNA1A	front	forward	CCGAGTGCCAAGAGGTAGAC	5677
		reverse	GACAGGAGCCCAGATGGAAC	
	back	forward	CTACACCCCCATTGCAGGAG	5521
		reverse	CCTAGAGATCCCCTGAACCG	
ATXN7	front	forward	GCTGAGCCCCCAAAAATGGT	2362
		reverse	AGCAAGCTGACACACTGCAT	
	back	forward	TACACGTCCCCACCCCTAAA	2652
		reverse	AACTGGGGAAAAGTCCCTGC	
ATXN8OS	front	forward	TCCTTCCAGAAATTAACAAGCTG	2429
		reverse	TGGATCTTGGAAGACTGTTCTTG	
	back	forward	ATGTGGCAACCCCTACAGATT	5698
		reverse	CAGTCATTCCCATTTGCAAAAACAC	
TBP	front	forward	TCCAGGGGTGTATATGGGCT	3661
		reverse	GGTGCAAGAGGCCTAGCTTT	
	back	forward	AGAATCCCTGGGAGTACAGCA	2732
		reverse	CCACAGCCCCCTTCTATGTG	
HTT	front	forward	ATGGAAAATTCGGGGGCCAAT	3457
		reverse	ACAGTGCTGGGGTGATTTGT	
	back	forward	CTAGCATGCTTGGGAGGGTC	3397
		reverse	ACCTAGGAGTGGGAAAACTATAACC	
FMR1	front	forward	GTCTGCAAGAAGCCCAATTT	4913
		reverse	GATCATGAGCCTGCTCTTCC	

	back	forward	GAGTTGCTTGTCCAGTGGGA	4527
		reverse	CGGGTCATGTGCGACTACTT	
C9orf72	front	forward	TTTTGCCAGGCTCTGTATGC	4292
		reverse	GCAGGGGTAGGAAATTAACCCA	
	back	forward	AAGCCATCTTTCATGCTGCT	1591
		reverse	TCCTCAGCCTGGACTTCACT	
RFC1	front	forward	TCCAGTTGAGGTTGTTGGAAG	3299
		reverse	ACAGCCCTGATCCACAGTTG	
	back	forward	GAGTGTGGGGGAGTTTAGGG	2097
		reverse	AGGCCAAAAACAGGCCAAAAT	
ATXN10	front	forward	CTCACCTGACCACCCCTCTA	2224
		reverse	GATACTGTCTCCTGGCACTGA	
	back	forward	TGCTATACCAGTCAGAATGCCAG	3640
		reverse	TGTCAACTGAGGGTATGCGT	
PPP2R2B	front	forward	TGACTGCAGGCAAGTTCATC	2124
		reverse	CATCCTACACAGCAGCCAGA	
	back	forward	TTGCTCTAGGTGCCAAGGATA	1925
		reverse	GAGGGGACTTTGCACCGATA	
BEAN1	front	forward	GCCCAGCAGAGTCTCATAGAA	1906
		reverse	CCTCGGAGAGGCAGTAACAG	
	back	forward	ACTTGGAACCAAGCCAAATG	1939
		reverse	ATTGCAGGAATGTGGTGACA	
NOP56	front	forward	TAGCCAGTGCATGCCTGTAG	3446
		reverse	TGGGACAGGGTCTCCTGTAG	
	back	forward	TCAGCGACATTGGATGCCTT	2226
		reverse	GCCCTGTCCATACCCATGTA	
DAB1	front	forward	CCTCAGGTCCCATGTGTCTT	1383
		reverse	GCCATCGCCCTAATTTTGGT	
	back	forward	TATGTCACGGGTGTCCCTCA	2293
		reverse	AATGGCATCCCTCCTAGCCT	
GLS	front	forward	CGGTCACAAACCAACCCTTG	2478
		reverse	GTTTCCTGCTTTAACTGGCCAAA	
	back	forward	GCTGTTTCCATCACGCAACT	5736
		reverse	TCGGGTGGGAAGACTAGGAT	
FGF14	front	forward	CGTGTCTGCATGTGAGATGG	2366
		reverse	CCTGGGTCTTCACATGGTCT	
	back	forward	AGACGCAACCTGAGGTAACAA	2356
		reverse	TCAGCAACCAATGAGTGACCTA	
CSTB	front	forward	TGGGGGAAGCTGGATTTAGG	2303
		reverse	CTTCAGAGTGAGGGTCTGCG	
	back	forward	TGCTGAGGAAGAAGGCACAT	3160
		reverse	TGGCTAGCCGTGAAAGTTGA	
ZFHX3	front	forward	ACTACTGGTGGGCATTGAAATACT	3951
		reverse	GGTTTTCTCCCTATAGTGTGGGTT	
	back	forward	GACGAAAAGAACAAGACTGCTTCA	5412

		reverse	GGTCTTCAAGAACAAGGTTTACGG	
NOTCH2NLC	front	forward	CATCCAACTTTGAGGGGGCA	5053
		reverse	CAGTGATCCAGTCCCATCCG	
	front	forward	AGGGCAAAACTATGTCAACAAGC	1523
		reverse	AACTGGGGAGCAATGCAAGA	
	back	forward	TGGGTGCAGTATGTCTGCATT	2234
		reverse	TGTGATTCTCCCACCAGTGC	
	back	forward	GTGGTGTATTGGGGGAGCAT	1712
		reverse	TGTGTCTTGACAGCGTCTGA	
PRNP	front	forward	ACATCTCAGGTGAAGACGCC	4006
		reverse	GCGAGGACATGGCATAGTGA	
	back	forward	TTCCAACCCGTGGTGTTCAA	2225
		reverse	AGGATCCCCAGGCTCTCATT	
ARX	front	forward	CCCTAGCCCCAAAGCTCCTA	1677
		reverse	GCGCATAGAAATCAGAGGGC	
	back	forward	CTTCTCCCCGAGGTGAATGG	1675
		reverse	TCTTCCCTCTTTCCCCCACA	
SAMD12	front	forward	CTCAAGGGACCAATCGACTCC	2078
		reverse	CAACACCACAGAAACGAGGAAGA	
	back	forward	GAACCCATGGACACAGGGAG	2565
		reverse	ATTTGGGCACCAAGACCCAA	
STARD7	front	forward	AAGGCAGACAGCATGAGTCC	2032
		reverse	TGGGATCGCCCAGAGTTCTA	
	back	forward	GACTCACCGGCTGCAAAAAG	2577
		reverse	AAGCAGTGGGTCAGATGCTC	
MARCHF6	front	forward	CAGGCAACTAGCTGGACCAA	1434
		reverse	AGAATTGCCTGAGTGGGAGC	
	back	forward	AGTGATGCTGGAAGTGCTCC	5229
		reverse	ACACCTTCATACAGCCCTGC	
TNRC6A	front	forward	CAGGGTCAGTTTGGCAACTC	2320
		reverse	TTTGAACCCACGTAGCCCTG	
	back	forward	GAACTCCTGGGCAATCCTCC	1137
		reverse	ACTTGGGGCCTCAGTTGTTT	
RAPGEF2	front	forward	GGTAGGCACAATCGGGTGAT	3291
		reverse	ACTCACCAGGAGTAGCACTGA	
	back	forward	TAGCCCAGACAACACCGAAC	2638
		reverse	GCTGTAACTGTGCAGGGACT	
YEATS2	front	forward	TTCCAGGTGCATTAATGGAGAGAA	7660
		reverse	GCATGTGGGCATATATTTGAGCTT	
	back	forward	AAGTCTGGTTCCTTTCCTTTCCTT	7206
		reverse	CAGGAGATGATGACCTAGATGGTG	
JPH3	front	forward	GATGCAGGGGGAGCTTTCTT	533
		reverse	TGCAGTCAGTTACTCCAGCG	
	back	forward	TCCACATTGGTCCGTGTCTC	793
		reverse	CTCCCAGTTGACCAGCAGAG	

AFF2	front	forward	CCATGTGCCCTTGGTGAAAT	1597
		reverse	GTCATGCAGGTTCGGGTAGT	
	back	forward	CTGTCAAGGCTGAGAAGGGG	1147
		reverse	GGCAGTTTTCCGCTTTGAGG	
SOX3	front	forward	CAGACGGGCAGATAAGCACT	5991
		reverse	CCTGGCATTTTCCCCGTAGA	
	back	forward	CATGGGTTCGGTGGTCAAGT	6248
		reverse	GTACACGCAGGCAGACTAGG	
DIP2B	front	forward	ATTGGACCCTTCACAATGCAAAAT	5942
		reverse	GCACCTTTACCAGTGCTGTTTATT	
	back	forward	CTGCTTCCAGCTGATTGTTGTTTA	6322
		reverse	GTATAGGGTATAGGTGCCAACAGG	
ZNF713	front	forward	CATGCTTGTGGATGACTCTGTAAC	6498
		reverse	ACAAGAGAAAACTCCATCTTCAAAA	
	back	forward	GTTCTTGTAACACGTTGGTGTCAT	6372
		reverse	CTCATTGTCTTGGTTTCACTGCTC	
CBL	front	forward	GGAGCAGATCTGTAGAAATAGCCA	6193
		reverse	CCCTTATTCCATCTGCCTGAAGTA	
	back	forward	CCTCCCTTAAGTCTTACTCCTTCA	6495
		reverse	CCAGGACCAATTTGACCATGATTT	
AR	front	forward	GGCCACATTCCTGACTGACA	2695
		reverse	GGTCATCCTCCCTCCACTCT	
	back	forward	GGGACAACTGGTGTTCCTGT	2586
		reverse	TCTTCCGTACGACAAAGGGC	
PRDM12	front	forward	CTCCAGACTGAGCAGGAACC	5714
		reverse	CTTTTCGGGCGTACGATTGC	
	back	forward	GACACCCCACCATTAGGCTC	6802
		reverse	GGCTGATCAACGGCATCTCT	
WDR7	front	forward	AGATTTTCATCTGTCTGACCTGCT	5739
		reverse	AGCATCTACTCTTACCTGCCAAAA	
	back	forward	CTGTGTTGTGTTCAGCGCTATTAT	6432
		reverse	GAATACAAACAGAACCACAGGAGC	
NIPA1	front	forward	GTACCAACACCAGCAATGTTCC	7869
		reverse	TCCTTTTATGGTCAGTGACTCTGG	
	back	forward	TTGTGGTTCTTGGCCATACTCTTA	5650
		reverse	TCTCACTCCGTAGCCTGG	
VWA1	front	forward	ATTCCCCTTTTCCAGGCGAG	3040
		reverse	TCTGTCCATCCATCCCTCGT	
	back	forward	AGGCACGTTCTGAGAATCCC	5317
		reverse	CCATAGGGCTCCCTGAAAGC	
DMPK	front	forward	CCCTGGTGGTGGTGTAATCC	1411
		reverse	GCTGCACAGTCTCCACTTCT	
	back	forward	AGTCCTGTGGCTCTGTGTACTA	491
		reverse	CTGGAGGGGCCACTTTAGATA	
NUTM2B-AS1	front	forward	TCCTTCAATGTAACAGAAGTAACCA	6600

		reverse	TTTGACTGGGTCCCCCACTA	
	back	forward	ACTACTCCCCACCTCAGTGATCC	6032
		reverse	AACTGATCAAGCCAGAGACCAG	
PABPN1	front	forward	CCAGCCAAATGGTCAAAGCC	6127
		reverse	ACTCCATAGCCCAACCAAGC	
	back	forward	GTGGCCATCCCAAAGGGTAA	6458
		reverse	GCCGATCAGGAGTGACTGAC	
CNBP	front	forward	CAGCACCCCGTCCTTTATCC	6892
		reverse	CGTGCCACTGGTGAATAGGG	
	back	forward	TTCTAGGGGGACAGGTGTCTTT	961
		reverse	AACAATTCTTACCACACACATCAG	
LRP12	front	forward	CTCGGGCCCAAAGCTTCTTA	6813
		reverse	GCTACGCATTGTCCTAGGCT	
	back	forward	CGGAGTGCCCCTCTTTCAAT	6964
		reverse	ACTGGCCGTCTCCTACTCAT	
GIPC1	front	forward	TGACTACGGTGAATGGTGCC	6680
		reverse	CAGCCTGAAGCTGGAAGACA	
	back	forward	CCTCCTTCCCGATTTTATAGCTCA	4421
		reverse	GCAAGACAGAGAAATACCTCAGGA	
PLIN4	front	forward	GAGAAGCGACTAAAAGGCACTCT	5928
		reverse	GGACACAAAGACAGCAAAACAATC	
	back	forward	GAAGCCCGGAAATTGTATTCTCAG	6171
		reverse	GAAAAGATGGGGAAGGAGTCAGAT	
ZIC2	front	forward	GGAGACAGTTGTGGGTCCTG	4459
		reverse	CGTTGAGCACATTCTGCGAG	
	back	forward	TCCCGGAGGTCACAATACCT	2439
		reverse	TGAGGGTTTTTGCCCGATCA	
XYLT1	front	forward	GATGAGACACCCTCGTCCAC	5800
		reverse	GACCAGACCTTTTCGCAAGC	
	back	forward	TGTTGCCAGAACGCTCTCTT	4708
		reverse	GTGATTTGGGGACTACGGGG	
FOXL2	front	forward	GTTTCCTGGCCTTAGACCCC	979
		reverse	GCCCTCGGGATTTCTCCAAA	
	back	forward	CAGCCCTGGTGAAGAAGAGG	1262
		reverse	ACGTGTAGCTGGTGAGTGTG	
HOXA13	front	forward	TCTGTGTCAACTCTCCGCAC	2535
		reverse	CCAGGCTCCCTGTGGAAAAT	
	back	forward	CAGGCAGCCAACAAACTGAC	2233
		reverse	TGTTTTGTTGCGGGCGTAAG	
HOXD13	front	forward	GAGTTCCTGTGCCCTCAGAC	1807
		reverse	TTCCAGCATAGCCTTTGGGG	
	back	forward	CACCATCCCCCACCTTACAC	1428
		reverse	AGCCCAGGCATAGAGACTCA	
PHOX2B	front	forward	GGCCTTGGGGTAATCCAACA	5798
		reverse	TAGAGAGGCCACGGTTCAGA	

	back	forward	GTGAGGTCGATCTTCAGGGC	3981
		reverse	CTCGACTAGGGCGTAGGGAT	
TCF4	front	forward	AAGCCAAATGGTTTATGAACAGCA	5741
		reverse	TGGTGCACATTGATTAAAAGGACC	
	back	forward	ATCCCTGACTCTTAACACCAACTC	7179
		reverse	GTACGATGACAGCACCTTGTATTG	

Table S16. Adaptive sampling BED file

Chr	Start	End	Target gene
12	6932419	6941271	*ATN1*
9	69033039	69041748	*FXN*
6	16322944	16332146	*ATXN1*
12	111590652	111603464	*ATXN2*
14	92064299	92075814	*ATXN3*
19	13201533	13212248	*CACNA1A*
3	63908291	63918156	*ATXN7*
13	70134809	70144568	*ATXN8OS*
6	170556802	170567008	*TBP*
4	3070150	3079720	*HTT*
X	147905849	147917374	*FMR1*
9	27567035	27579934	*C9orf72*
4	39343362	39353374	*RFC1*
22	45790160	45800365	*ATXN10*
5	146874004	146882985	*PPP2R2B*
16	66490940	66502525	*BEAN1*
20	2647619	2657303	*NOP56*
1	57362564	57371471	*DAB1*
2	190876543	190887430	*GLS*
13	102153291	102166924	*FGF14*
21	43771993	43781689	*CSTB*
16	72781103	72792516	*ZFHX3*
1	149386630	149398172	*NOTCH2NLC*
20	4693389	4704003	*PRNP*
X	25009435	25018182	*ARX*
8	118360904	118372462	*SAMD12*
2	96192357	96202438	*STARD7*
5	10351801	10360818	*MARCHF6*
16	24608987	24617921	*TNRC6A*
4	159335854	159347916	*RAPGEF2*
3	183706443	183719559	*YEATS2*
16	87600093	87609192	*JPH3*
X	148496407	148505575	*AFF2*
3	181708220	181717723	*SOX3*
12	50498184	50511349	*DIP2B*
7	55880761	55892439	*ZNF713*
11	119201125	119211006	*CBL*
X	67540174	67549624	*AR*
9	130677215	130687739	*PRDM12*
18	57019595	57029662	*WDR7*
15	22779040	22792265	*NIPA1*
1	1431673	1440174	*VWA1*

19	45765616	45775133	*DMPK*
10	79819073	79834400	*NUTM2B-AS1*
14	23316393	23327552	*PABPN1*
3	129167564	129177076	*CNBP*
8	104583324	104593148	*LRP12*
19	14491078	14500530	*GIPC1*
19	4504672	4519019	*PLIN4*
13	99980276	99989827	*ZIC2*
16	17466557	17475315	*XYLT1*
3	138941676	138950217	*FOXL2*
7	27194894	27204432	*HOXA13*
2	176088736	176097202	*HOXD13*
4	41741799	41750181	*PHOX2B*
18	55217865	55643677	*TCF4*

Table S17. Known STR disease repeat expansion information

Gene	Chr	Start	End	Known motif	Pathogenic expansion repeat number
ATN1	chr12	6936717	6936775	CAG	48
FXN	chr9	69037275	69037314	GAA	66
ATXN1	chr6	16327636	16327723	CAG	38
ATXN2	chr12	111598950	111599019	CAG	32
ATXN3	chr14	92071011	92071052	CAG	52
CACNA1A	chr19	13207858	13207897	CAG	18
ATXN7	chr3	63912685	63912716	CAG	33
ATXN8OS	chr13	70139383	70139428	CAG/TAG	73
TBP	chr6	170561907	170562017	CAG	42
HTT	chr4	3074876	3074941	CAG	35
FMR1	chrX	147911979	147912111	CGG	54
C9orf72	chr9	27573485	27573546	GGGGCC	23
RFC1	chr4	39348425	39348483	AAGGG/ACAGG	399
ATXN10	chr22	45795355	45795424	ATTCT	279
PPP2R2B	chr5	146878729	146878758	CAG	50
BEAN1	chr16	66495475	66495509	TGGAA	109
NOP56	chr20	2652733	2652775	GGCCTG	649
DAB1	chr1	57367044	57367125	ATTTC	30
GLS	chr2	190880873	190880920	GCA	300
FGF14	chr13	102160577	102162726	GAA	250
CSTB	chr21	43776429	43776470	CCCCGCCCCGCG	30
ZFHX3	chr16	72787693	72787757	GCC	40
NOTCH2NLC	chr1	149390803	149390842	CGG	65
PRNP	chr20	4699379	4699380	CCTCAGGGCGGTGGTGGCTGG	7
ARX	chrX	25013654	25013697	GCC	16
SAMD12	chr8	118366813	118366918	TTTCA	104
STARD7	chr2	96197067	96197124	ATTTC	149
MARCHF6	chr5	10356339	10356411	TTTCA	699
TNRC6A	chr16	24613439	24613532	TTTTA	18
RAPGEF2	chr4	159342527	159342618	TTTTA	17
YEATS2	chr3	183712113	183712552	TTTCA	0
JPH3	chr16	87604283	87604329	CTG	39
AFF2	chrX	148500605	148500753	CCG	200
SOX3	chr3	181712415	181712456	GCG	14
DIP2B	chr12	50505001	50505022	CGG	350
ZNF713	chr7	55887537	55887689	CGG	22
CBL	chr11	119206289	119206322	CCG	3
AR	chrX	67545317	67545419	CAG	37
PRDM12	chr9	130681606	130681641	GCG	17
WDR7	chr18	57024413	57025039	NNN	1
NIPA1	chr15	22786677	22786701	GCG	10

VWA1	chr1	1435799	1435820	GGCGCGGAGC	2
DMPK	chr19	45770205	45770266	CTG	49
NUTM2B-AS1	chr10	79826364	79826403	CGG	15
PABPN1	chr14	23321472	23321511	GCG	6
CNBP	chr3	129172577	129172656	CCTG	49
LRP12	chr8	104588965	104588999	CGG	89
GIPC1	chr19	14496029	14496104	CGG	69
PLIN4	chr19	4510488	4513581	NNN	31
ZIC2	chr13	99985449	99985494	GCG	24
XYLT1	chr16	17470869	17470967	GGC	99
FOXL2	chr3	138946022	138946062	GCG	21
HOXA13	chr7	27199827	27199967	GCG	23
HOXD13	chr2	176093058	176093099	GCG	21
PHOX2B	chr4	41745976	41746022	GCG	23
TCF4	chr18	55586155	55586227	TGC	50

Table S18. STRiker configuration parameters used in benchmark analysis

**Program running paramter**	**Value**
MINIMUM_MOTIF_LENGTH	3
MAXIMUM_MOTIF_LENGTH	30
CONSECUTIVE_THRESHOLD	10
MINIMUM_MOTIF_COVERAGE	5
COVERAGE_THRESHOLD	30

Table S19. STRiker computational performance benchmarking

Sequencing date	File name	BAM line number	Real time (seconds) 1st	Real time (seconds) 2nd	Real time (seconds) 3rd	Average time (s)
20240512	240512_LYJ-M_Cas9_multi_sorted.bam	479526	14.111	14.409	14.078	14.199
20241119	241119_OMS_Cas9_nonAS_sorted.bam	2357895	31.356	30.909	30.952	31.072
20240511	240511_LYJ-F_Cas9_multi_sorted.bam	792831	32.006	32.045	32.208	32.086
20250415	250415_G1622_Cas9_tier-all_adaptive.bam	1699930	32.908	32.958	32.7	32.855
20231218	231218_MJS_G1254_bae_sorted.bam	1711356	24.027	23.986	24.006	24.006
