# Supplementary material for: Optimized Cas9‐Enriched Nanopore Sequencing and Analysis Workflow for Clinical Diagnosis of Repeat Expansion Disorders
Source: Adv Sci (Weinh). 2026 Aug 29:e77475. Online ahead of print. doi: 10.1002/advs.77475 (PMC13525614; doi:10.1002/advs.77475)
Supplement: Supplementary file 2 [file ADVS-9999-e77475-s003.docx]

**Supplementary Materials**

**Optimized Cas9-enriched nanopore sequencing and analysis workflow for clinical diagnosis of repeat expansion disorders**

Seungbok Lee, Chanju Jung, Minjeong Kim, Narae Kim, Gue-Ho Hwang, Soon-Tae Lee, Kon Chu, Sang Kun Lee, Han-Joon Kim, Jong Hee Chae, Sangsu Bae, Jangsup Moon

**List of Supplementary Materials:**

Figure S1 to S12 for multiple supplementary figures

Table S1 to S19 for multiple supplementary tables

**Figure S1. Overview of STR loci included in the 56-gene panel.**

Schematic representation of disease-associated short tandem repeat (STR) loci categorized by their genomic location (5′ untranslated region (UTR), exon, intron, or 3′ UTR) and repeat motif type. Each box lists representative disorders, associated genes, and repeat motifs. Symbols indicate the major clinical categories. This panel comprises 56 known pathogenic or candidate STR loci encompassing a wide range of repeat motifs implicated in neurological, muscular, and developmental disorders. Created with BioRender.com

**Figure S2. sgRNA screening for high cleavage efficiency via *in vitro* cleavage assay.**

Cleavage efficiency of sgRNAs targeting upstream and downstream regions of repeat expansion disease-associated gene regions of interest (ROIs), assessed by *in vitro* cleavage assay (*n*=1). Three or five different sgRNAs were tested for each region.

**Figure S3. Impact of guide RNA selection on nCATS sequencing coverage and guide RNA optimization.**

(A) Impact of guide RNA selection on nCATS sequencing coverage. Raw coverage depth obtained using selected and non-selected sgRNA pairs for representative STR loci. The dashed line indicates 30× coverage. (B) Schematic illustration of sgRNA and crRNA:tracrRNA guide RNA formats (left). Comparison of in vitro cleavage efficiencies between sgRNA and crRNA:tracrRNA for representative guide RNAs targeting NOTCH2NLC and NOP56 (right). Each dot represents an independent experiment. (C) Cost comparison between sgRNA and crRNA:tracrRNA for constructing the 56-locus STR panel. (D) In vitro cleavage efficiencies measured across different crRNA:tracrRNA ratios for representative guide RNAs targeting the upstream and downstream regions of ATXN1.

**Figure S4. Schematic diagram with a comparison of the optimized nCATS protocol with the conventional protocol.**

The optimized protocol employs sequential Cas9 cleavage at both ROI termini, followed by bead purification, rather than simultaneous Taq polymerase and Cas9 treatment. Further optimization includes dA-tailing using a Klenow fragment (exo-) (NEB) and an extended nanopore adapter ligation time to 60 minutes.

**Figure S5. Optimization of ligation time and evaluation of adaptive sampling in the multiplexed nCATS workflow.**

(A) Comparison of raw coverage depth across all 56 STR loci under 10-, 20-, and 60-min ligation conditions without adaptive sampling (−AS). Each box represents the distribution of raw coverage obtained from independent sequencing runs for each STR locus. The dashed line indicates 30× coverage. STR loci are grouped into the Ataxia STR panel (orange) and the Extended STR panel (blue). The 60-min ligation condition showed slightly more uniform coverage across the STR panel. (B) Representative IGV visualization comparing sequencing reads generated without adaptive sampling (−AS) and with adaptive sampling (+AS). Adaptive sampling reduced reads originating from non-target regions while increasing the proportion of reads mapped to target regions. Red and blue boxes indicate representative target and non-target regions, respectively.

**Figure S6. Coverage distribution across the 62-locus STR panel in patient-derived samples.**

(A) Heatmap showing the average raw coverage depth across the 62 STR loci in patient-derived samples analyzed using the optimized nCATS workflow. Color intensity represents the average raw coverage depth.(B) Distribution of raw coverage depth across the 62 STR loci in patient-derived samples. Each box represents the distribution of raw coverage depth obtained from independent patient samples for each STR locus. The dashed line indicates 30× coverage. STR loci are grouped into the Ataxia STR panel (orange) and the Extended STR panel (blue).

**Figure S7. STRiker identifies a pathogenic length repeat expansion at *ATXN8OS* and *RFC1* that was not detected by HMMSTR.**

Heatmaps show per-read motif composition in patient P15, with each row representing a single read and colors indicating repeat motif identity (legend, right). STRiker resolved pathogenic-length expansions at both loci that HMMSTR was unable to call.

**Figure S8. KDE read length distributions at expansion sites for newly diagnosed patients using the STRiker pipeline.**

Read lengths span the repeat locus plus 500 bp flanking regions on each side. Gaussian-fitted peaks are shown in blue (primary) and green (secondary). The red line indicates the threshold for pathogenic expansion.

**Figure S9. Repeat motif composition analysis in family members identified through cascade screening.**

Heatmaps of the repeat motif composition for newly diagnosed patients and their relatives identified by cascade screening. Each panel represents long-read alignments at disease-associated loci, with distinct colors indicating different motif types for each gene. *PRNP* repeat expansions consist of an octapeptide motif of 24 bp (PHGGGWGQ), for which no distinct motif colors are applied.

**Figure S10. Case of contraction during paternal inheritance in a family with *NOTCH2NLC*-related neuronal intranuclear inclusion disease (NIID).**

Long-read alignment and methylation profiles of each family member (N1_F, N1_M, N1, and N1_S). The father (N1_M) carried a long GGC repeat with hypermethylation and was asymptomatic, whereas the patient (N1) inherited a shorter allele with hypomethylation, which is likely associated with the development of neuronal intranuclear inclusion disease (NIID). The older sister (N1_S) of the patient also carried a hypomethylated expanded allele, was clinically affected, and died. Created with BioRender.

**Figure S11. Concordance between STRiker and existing tools. (CHM13, 439 haploid loci).**

Scatter plots comparing STR repeat-count calls from STRiker (y-axis) against RepeatHMM, Straglr, and HMMSTR (x-axis) across 439 benchmarked loci. The dashed line indicates the line of identity (y = x). Pearson correlation coefficient (R), mean absolute difference (MAD), and mean bias (STRiker minus comparator tool) are shown for each comparison.

**Figure S12. Bland-Altman analysis of agreement between STRiker and existing tools.**

For each pairwise comparison, the difference between STRiker and the comparator tool’s repeat-count call is plotted against the mean of the two calls. The solid black line indicates the mean bias; red dashed lines indicate the 95% limits of agreement (bias ± 1.96 SD). Narrower limits of agreement indicate closer concordance between tools.

**Fig. S13. Genotyping accuracy of STRiker, HMMSTR, Straglr, and RepeatHMM on homozygous NA12878 loci.**

Genotyping accuracy statistics for the same four tools against a haplotype-resolved homozygous NA12878 benchmark set (n = 4,731 loci). Miscall rate was counted over the intersection of loci genotyped by all four tools and expressed as a percentage of each tool's own total calls.

**Fig. S14. Correctly-genotyped locus overlap among tools on homozygous NA12878 loci**

Venn diagram of loci genotyped correctly by each tool against a homozygous NA12878 benchmark set (n = 13,860 chromosome 1 loci). Correct genotyping was defined as loci where both the H1 and H2 calls fell within a data-derived tolerance of ±4 copies of the truth. Bars indicate the number of loci in each intersection; filled circles below each bar denote which tool(s) contributed to that intersection, with connecting lines linking co-occurring tools.
